# Novel Convenient Approach to 6-, 7-, and 8-Numbered Nitrogen Heterocycles Incorporating Endocyclic Sulfonamide Fragment

**DOI:** 10.3390/molecules25122887

**Published:** 2020-06-23

**Authors:** Oleksandr Shalimov, Eduard Rusanov, Oksana Muzychka, Petro Onys’ko

**Affiliations:** 1 Department of Heteroatom Chemistry, Institute of Organic Chemistry, National Academy of Sciences of Ukraine, 5 Murmans’ka, 02660 Kyiv, Ukraine; ashal@ukr.net (O.S.); xray@ioch.kiev.ua (E.R.); 2 Department of Mechanisms of Bioorganic Reactions, V.P. Kukhar Institute of Bioorganic Chemistry and Petrochemistry, National Academy of Sciences of Ukraine, 1 Murmans’ka, 02094 Kyiv, Ukraine; oksana@bpci.kiev.ua

**Keywords:** electrophilic heterocyclization, 1,2,4-thiadiazines, 1,2,4-thiadiazepines, 1,2,4-thiadiazocines, *N*,*S*-heterocyclic *S*,*S*-dioxides, imidoyl chlorides

## Abstract

A new effective method for the construction of nitrogen heterocycles incorporating endocyclic pharmacophore sulfonamide fragment, based on the use of easy accessible *N*-(chlorosulfonyl)imidoyl chloride, CCl_3_C(Cl)=NSO_2_Cl (**1**), has been developed. Thus, a reaction of **1** as bielectrophilic 1,3-C–N–S reagent with benzylamines that act as 1,4-N–C–C-C binucleophiles, affords respective 1,2,4-benzothiadiazepine-1,1-dioxides. On the other hand, **1** reacts with alkenyl amines with the formation of respective *N*-alkenyl amidines undergoing Lewis acids initiated intramolecular cyclization to afford derivatives of 1,2,4-thiadiazines and 1,2,4-thiadiazocines bearing a halomethyl group able for further functionalization. The first examples of electrophilic heterocyclization of the chlorosulfonyl group onto an alkenyl or alkynyl group have been revealed.

## 1. Introduction

**Figure 1:**
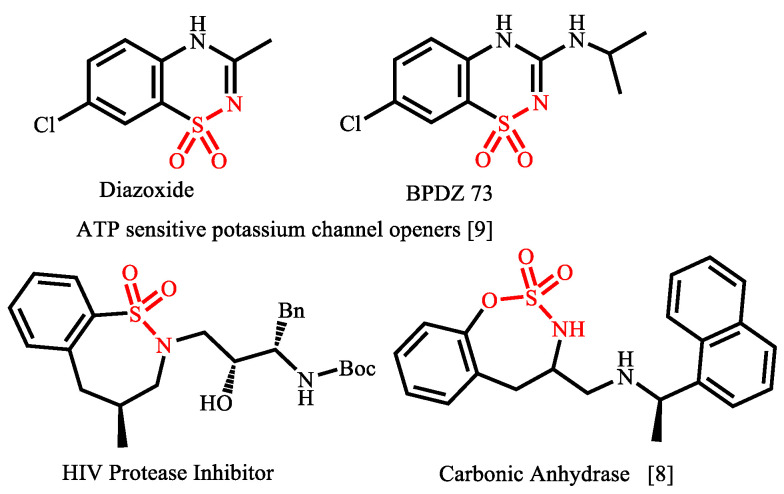
Examples of biorelevant compounds incorporating endocyclic sulfonamide fragment.

Nitrogen heterocycles are part of a huge number of marketed drugs. They are widely used in modern drugs design [1]. In particular, heterocyclic compounds of different size bearing an endocyclic pharmacophore sulfonamide fragment reveal a wide range of biological effects, including enzymes inhibition, antihypertensive [1,2,3,4], antimicrobial [5], and antiviral activity [6]. Benzothiadiazine-*S*,*S*-dioxides were used in the design of phosphodiesterase 7 inhibitors [7], HIV protease inhibitors [8], ATP-sensitive potassium channel openers exhibiting different tissue selectivity profiles [9], potent antiviral agents against HCMV, antagonists of the human CCR5 receptor [6], nonnucleoside human cytomegalovirus selective inhibitors [10], α_1_-adrenoceptor antagonists [11], drugs for treatment of cancer and the early stages of Alzheimer disease [12], human herpes virus (HHV-6) and Varicella-Zoster virus [10], and ligands for complexation TACe inhibitors [13] and others. Bridged sultams (bridged sulfonamides) besides applications in medicinal chemistry have been used as a template in stereoselective synthesis enabled by the rapid scission of the N-SO_2_ bond [14,15]. Some examples of biorelevant cyclic sulfonamides are shown in Figure 1.

Furthermore, cyclic *N*-sulfonylamides have been used as key building blocks in the synthesis of chiral auxiliaries, chiral ligands for the preparation of chiral ruthenium(II)-catalysts ([8] and references therein).

Many methods have been developed for the synthesis of fused six-membered cyclic sulfonamides, although each of them has some drawbacks and limitations (see e.g., [2,12] and references therein). The main method for the preparation of monocyclic 1,2,4-thiadiazine 1,1-dioxides is the condensation of 2-chloroethyl sulfonyl chloride with amidines [16]. The sulfa-Staudinger cycloaddition between methanesulfonyl sulfene and *N*-methyl imines produces mixtures of respective 1,2,4-thiadiazine 1,1-dioxides and 4-aza-δ-sultams in low yields [17]. The only example of the intramolecular addition of a sulfonyl chloride group to a cyanamide fragment producing monocyclic 1,2,4-thiadiazine dioxides derivative was also described [18]. On the other hand, the synthetic methods for medium sized heterocycles are less common as their formation is often hampered by enthalpic and entropic factors [19]. There existed only few approaches to 1,2,4-benzothiadiazepine-1,1-dioxides. In 1988, Palmisano et al. described electrochemical heterocyclization of *o*-tolenesulfonamides with nitriles, leading to 3-alkyl-4,5-dihydro-1,2,4-benzothiadiazepine dioxides [20]. The method requires special equipment and is limited to *N*-4 unsubstituted derivatives. Interesting route to benzodiazepines, based on carbodiimide insertion into saccharin via a two atom ring expansion, was reported recently [21]. A few benzothiadiazepines also were synthetized by cyclisation of 2-chlorosulfonylbenzoyl chloride with urea and thiourea [22]. These methods are limited by set of reagents, scope of reactions, and the use of column chromatography for purification of final products. Synthesis of medium sized *N*,*S*-heterocycles have been discussed in recent experimental and review papers [13,19,23]. It can be concluded that synthetic methods for the monocyclic thiadiazines and especially for seven- and eight numbered heterocycles with the endocyclic sulfonamide fragment are limited and rather specific for each particular type of compounds. To the best of our knowledge, 1,2,4-thiadiazocine ring system was not known so far. 

Therefore, elaboration of new effective synthetic strategies for construction of mono- and polycyclic systems bearing pharmacophore sulfonamide moiety remains a challenging task.

**Scheme 1:**
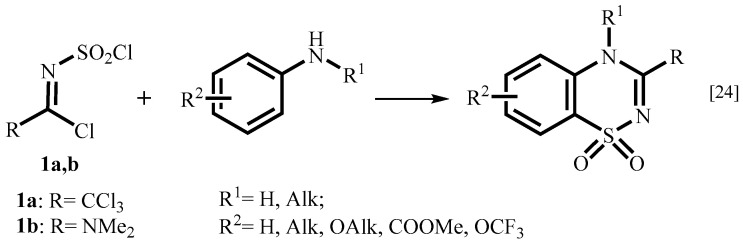
Synthesis of 1,2,4-benzothiadiazine 1,1-dioxides.

Recently, we have developed a convenient method for the preparation of novel C-N-S-bielectrophilic reagent, *N*-chlorosulfonyltrichloroacetimidoyl chloride **1a**, and demonstrated its utility for the synthesis of 1,2,4-benzothiadiazine 1,1-dioxides derivatives [24] (Scheme 1). The important advantage of C-N-S bielectrophilic reagent 1a is that the presence of strongly electron withdrawing trichloromethyl group at the C=N bond leads to a large difference between the electrophilicity of C- and S-electrophilic sites predetermining high regioselectivity in reactions with nucleophilic agents. Noterworthy, for analogous *S*,*N*-bielectrophilic *N*-chlorosulfonylchloroformamidine **1b** (R = NMe_2_) (Scheme 1) regioselectivity of reactions with nucleophiles depends on the nature of nucleophile ([24] and references therein).

In the present work, we describe the synthesis of monocyclic and fused six-, seven-, and eight membered heterocycles bearing sulfonamide moiety, based on the use of *N*-(chlorosulfonyl)acetimidoyl chloride **1a**.

## 2. Results and Discussion

Electrophilic intramolecular cyclization of unsaturated compounds is a powerful tool for the construction of various heterocyclic systems. Much to our surprise, this methodology was never applied to unsaturated compounds with double or triple carbon-carbon bond as nucleophilic site and sulfonyl chloride as electrophile. Although intermolecular addition of sulfonyl chlorides to olefins under free-radical conditions (atom transfer radical addition) is well documented and widely used for the preparation of α-chloro sulfones and vinyl sulfones (see e.g., [25,26,27,28,29] and references therein), there is only one publication reporting small-scale homolytic intramolecular heterocyclization of pent-4-sulfonyl chloride proceeding at high temperature (AIBN-CuCl_2_, MeCN, 170 °C) and resulting in 3-chlorotetrahydrothiopyran 1,1-dioxide in a low yield (17%) [30]. Obviously, this heterocyclization is of low preparative value and has rather theoretical meaning.

**Scheme 2:**
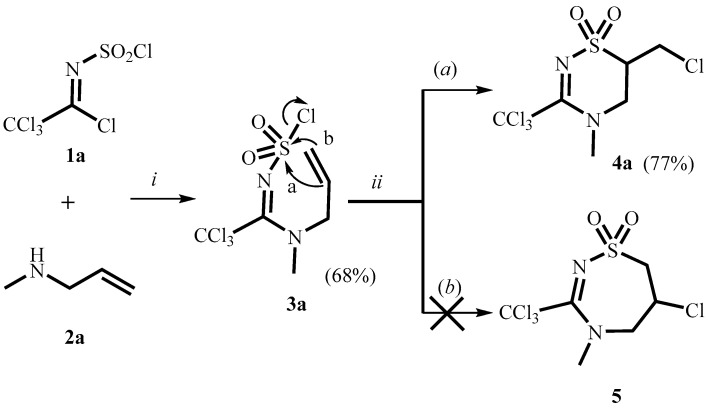
Intramolecular heterocyclization onto internal alkenyl carbon atom of **3a** (route a); cylization onto terminal alkenyl carbon (route b) was not detected. Reagents and conditions: (i) Et_3_N, CH_2_Cl_2_, 0–20 °C, 2 h; (ii) AlCl_3_, CH_2_Cl_2_, r.t., 24 h.

We believed that the reaction of *N*-chlorosulfonyl imidoyl chloride **1** with one equivalent of an unsaturated amine would result in regioselective substitution of the chlorine atom at the C=N bond and the formation of respective unsaturated amidine, in which the location and polarity of sulfonyl chloride and olefinic fragment are favorable for intramolecular electrophilic heterocyclization. Indeed, it was found that **1a** reacted with *N*-methyl allyl amine **2a** in the presence of triethylamine to form amidine **3a**, bearing C=C bond and sulfonyl chloride moiety in the same molecule (Scheme 1). To our delight, addition of anhydrous aluminum chloride initiated clean intramolecular cyclization affording 1,2,4-thiadiazine 1,1-dioxide derivative **4a** in 77% yield (Scheme 2, route a). Alternative direction involving cyclization on terminal carbon atom of the C=C bond (route b) was not observed. It is worth of noting that Cu catalyzed photoredox chlorosulfonation of terminal alkenes and alkynes proceeds in accordance with path b, i.e., the addition of the sulfonyl group occurs on the terminal carbon atom [30].

Next, we tested some other conditions for the intramolecular heterocyclization of **3a**. In the absence of Lewis acid, **3a** remains unchanged upon heating in dichloroethane (Table 1, entry 1). Aluminum and titanium chlorides gave similar results, whereas boron trifluoride etherate or zinc chloride are non-effective for the heterocyclization (entries 2–5). From the preparative point of view, aluminum trichloride is the most convenient reagent for the heterocyclization.

**Scheme 3:**
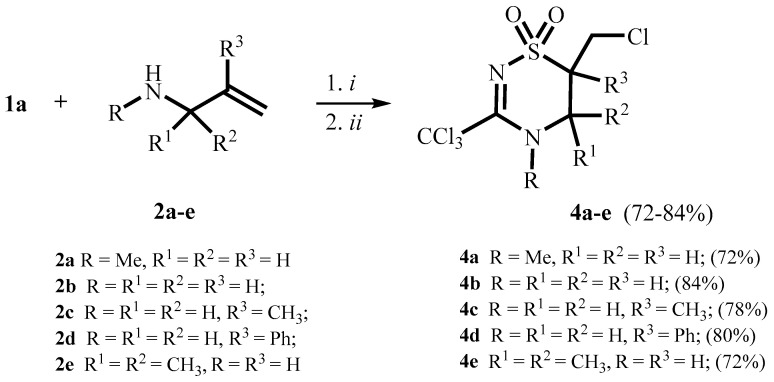
One-pot synthesis of 5,6-dihydro-4H-1,2,4-thiadiazine 1,1-dioxides **4a**–**e**. Reagents and conditions: (i) Et_3_N, CH_2_Cl_2_, 0–20 °C, 2 h; (ii) AlCl_3_, CH_2_Cl_2_, r.t., 24 h.

The electrophilic heterocyclization found can be extended to other 2-alkenyl amines. As is seen from Scheme 3, the reaction proceeds by the same manner for primary and secondary amines **2** (R = H, Me). Noteworthy, 2-alkenylamines substituted at α- or β-position to nitrogen atom (R^1^, R^2^ = H, Me, Ph) can also be successfully used in this novel heterocyclization. Moreover, the preparation of hydrogenated 1,2,4-thiadiazine 1,1-dioxides **4a**–**e** from dichloride **1a** and respective alkenylamines **2** can be realized in “one-pot” procedure, without isolation of intermediate amidines of type **3a**.

**Figure 2:**
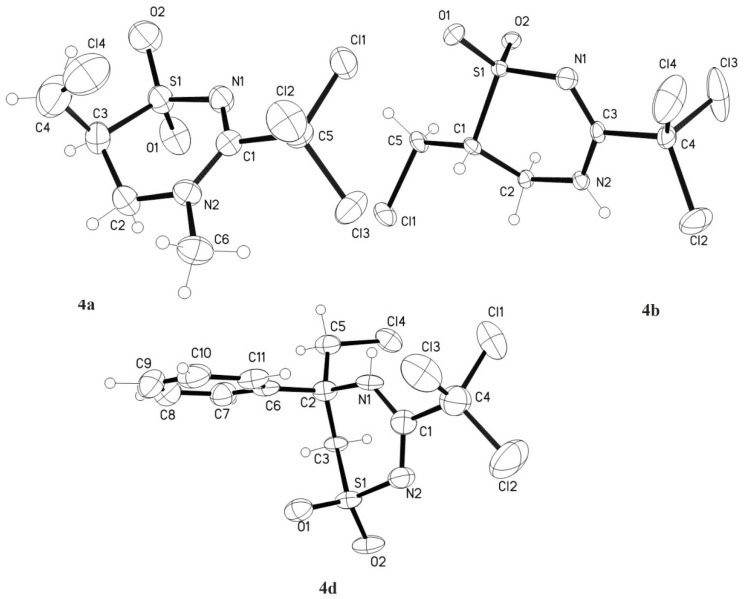
Molecular structure of compounds **4a**,**b**,**d** including thermal displacement ellipses with 50% probability.

Molecular structure of compounds **4a**,**b**,**d** was unambiguously proved by X-ray crystallographic analysis (Figure 2). Noteworthy, in compounds **4a**,**b**,**d** C=N bond lengths (1.289–1.299 Å) are close to standard mean value (1.28 Å) for C=N double bond. Mean value for formally single C-N bonds (1.319 A) are only a little longer and typical for conjugated C-N bonds, indicating on strong delocalization of the electron density in N=C(CCl3)-N system for compounds **4**.

**Scheme 4:**
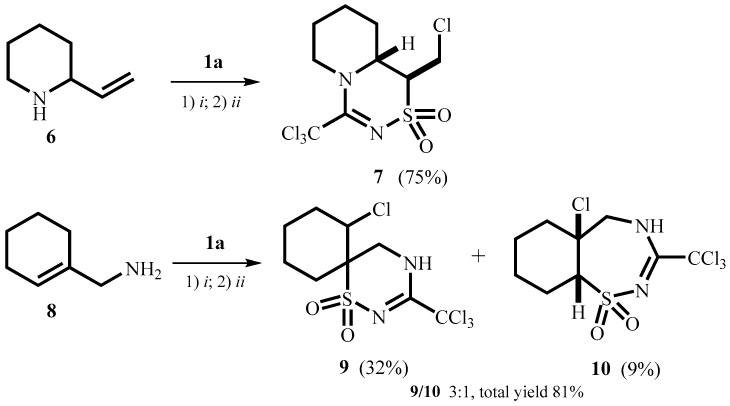
One-pot synthesis of **7**, **9**, **10** via heterocyclisation of isomeric alkenylamines **6** and **8** with 2,2,2-trichloro-*N*-(chlorosulfonyl)acetimidoyl chloride **1a**. Reagents and conditions: (i) Et_3_N, CH_2_Cl_2_, 0–20 °C, 2 h. (ii) AlCl_3_, CH_2_Cl_2_, r.t., 24 h.

**Figure 3:**
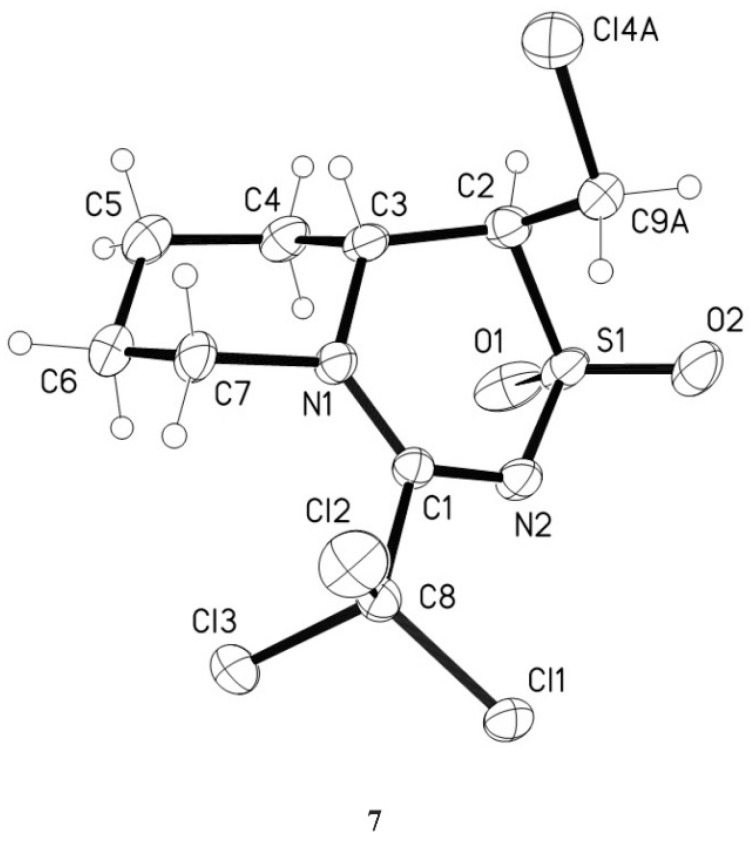
Molecular structure of 1-chloromethyl-4-trichloromethyl-1,6,7,8,9,9a- hexahydropyrido[1,2-*d*][1,2,4]thiadiazine-2,2-dioxide **7**: Including thermal displacement ellipses with 50% probability.

**Figure 4:**
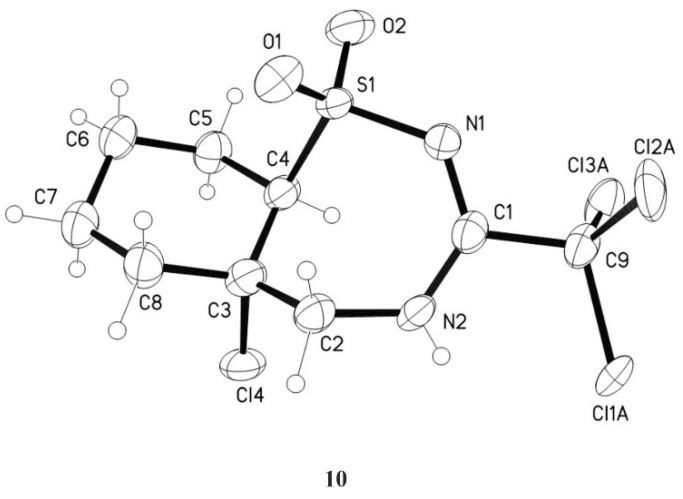
Molecular structure of 5a-chloro--3-trichloromethyl-4,5,5a,6,7,8,9,9a- octahydro-1,2,4-benzothiadiazepine-1,1-dioxide **10** including thermal displacement ellipses with 50% probability.

Incorporation of the amino group or C=C bond into the cyclic structure allows the preparation of fused heterocycles bearing 1,2,4-thiadiazine fragment. Thus, the reaction of readily accessible 2-vinylpyperidine **6** with the dichloride **1a** under the same conditions proceeds regioselectively to afford hydrogenated pyrido[1,2-*d*][1,2,4]thiadiazine 2,2-dioxide **7** in 75% yield (Scheme 4). The structure of novel pyrido[1,2-*d*][1,2,4]thiadiazine heterocyclic system **7** was unambiguously proved by X-ray crystallographic analysis (Figure 3). At the same time, the heterocyclization with isomeric cyclohexenylmethyl amine **8** proceeds by two competitive pathways to form regioisomeric spirocyclic thiadiazine **9** and fused thiadiazepine (**10**) in a ratio of about 3:1. The formation of a seven-numbered heterocycle **10** was confirmed by X-ray crystallographic analysis (Figure 4). The creation of six or seven membered heterocycles obviously resulted from involvement in the cyclization of C-1 or C-2 atom of the double bond. Compounds **9** and **10** were separated by chromatography and isolated in 32% and 10% yields, respectively.

**Scheme 5:**
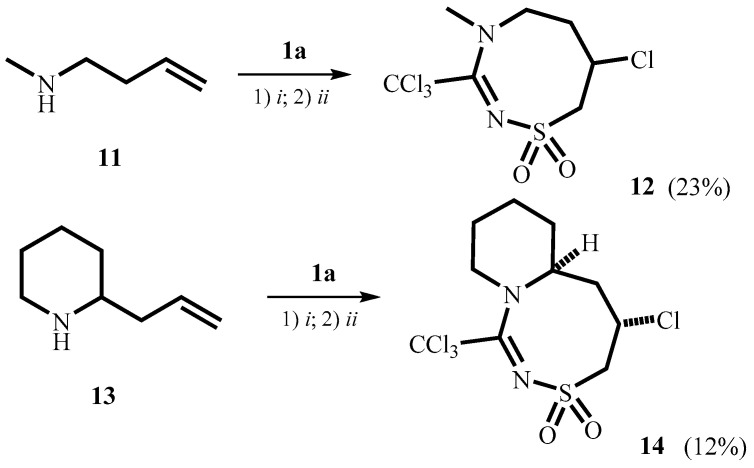
One-pot synthesis of **12** and **14** via heterocyclisation of alkenylamines **11** and **13** with 2,2,2-trichloro-*N*-(chlorosulfonyl)acetimidoyl chloride **1a** on the terminal atom of the C=C bond. Reagents and conditions: (i) Et_3_N, CH_2_Cl_2_, 0–20 °C, 2 h; (ii) AlCl_3_, CH_2_Cl_2_, r.t., 24 h.

**Figure 5:**
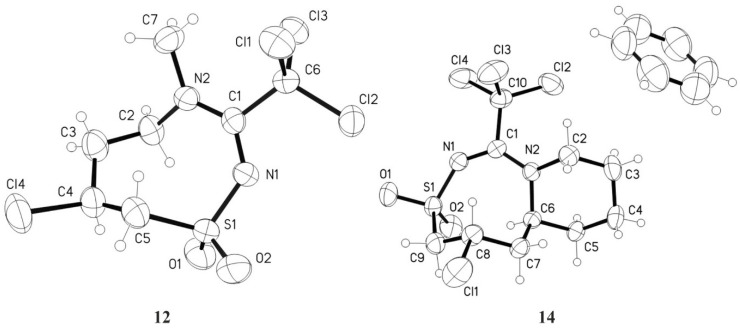
Molecular structure of thiadiazocine dioxides **12**, **14** including thermal displacement ellipses with 50% probability.

Next, we elongated chain between the amino nitrogen atom and double bond. Surprisingly, the heterocyclization of imidoyl chloride **1a** with acyclic **11**, or cyclic alkenylamine **13** afforded, though in low yields, difficultly accessible thiadiazocine dioxides **12**, **14**, rather than expected seven-membered heterocyles, diazepines (Scheme 5). In contrast to Scheme 2 and Scheme 3, cyclization in this case proceeds on the terminal *sp*^2^ C atom of the alkenylamines **11**, **13**. Formation of novel 1,2,4-thiadiazocine and pyrido[1,2-*d*][1,2,4]thiadiazocine heterocyclic systems **12**, **14** in Scheme 5 was unambiguously proved by X-ray crystallographic analysis (Figure 5). Notably, cyclization of **1a** with **13** proceeds diastereoselectively to form diastereomer **14** with (S*,S*)-relative stereochemistry of C-5 and C-6a stereogenic centers.

**Scheme 6:**
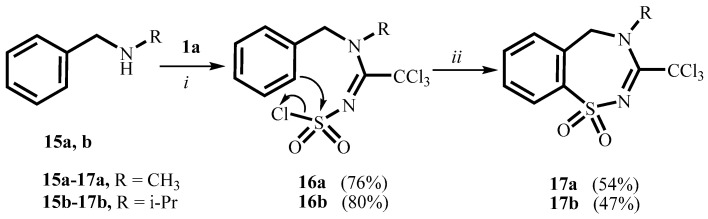
Synthesis of 4,5-dihydro-1,2,4-benzothiadiazepine-1,1-dioxides **17a**,**b**. Reagents and conditions: (i) Et_3_N, CH_2_Cl_2_, 0–5 °C, 2 h; (ii) AlCl_3_, CH_2_Cl_2_, reflux, 16 h.

For the synthesis of benzothiadiazepines we developed another approach based on the use of benzylamines. It was found that *N*-alkyl benzylamines **15a**,**b** regioselectively reacted with imidoyl chloride **1a** to afford amidines **16a**,**b**. In the presence of AlCl_3_ the latter undergo intramolecular sulfonation of benzene ring with the formation of 4,5-dihydro-1,2,4-benzothiadiazepine-1,1-dioxides **17a**,**b** (Scheme 6). The heterocyclization in this case requires more hard conditions (CH_2_Cl_2_, reflux) than for alkenyl amines (CH_2_Cl_2_, r.t.), due to less reactivity of the double bond, incorporated in the benzene ring in comparison with the C=C bond of alkenyl amines.

**Scheme 7:**
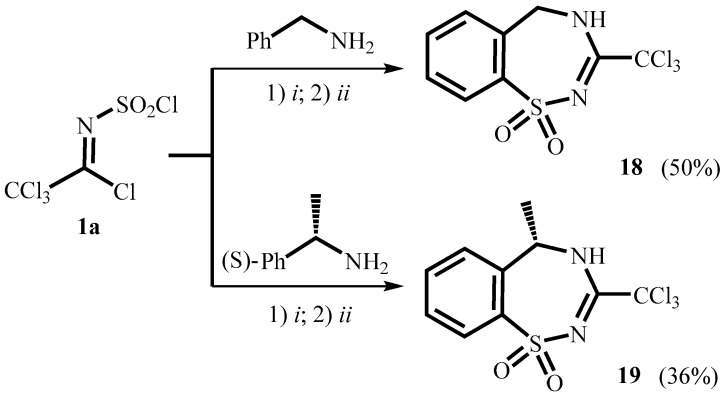
One-pot synthesis of *N*-unsubstituted **18** and optically active **19** 4,5-dihydro-1,2,4-benzothiadiazepine-1,1-dioxides. Reagents and conditions: (i) Et_3_N, CH_2_Cl_2_, 0–5 °C, 40 min; (ii) AlCl_3_, CH_2_Cl_2_, reflux, 16 h.

Reaction can be extended to primary benzylamines. In this case, it is more convenient to carry out the synthesis as “one-pot” procedure, without isolation of intermediate unstable NH-amidines. The use of chiral (*S*)-phenylethyl amine provides enantiomerically pure benzothiadiazepine **19** (Scheme 7).

**Figure 6:**
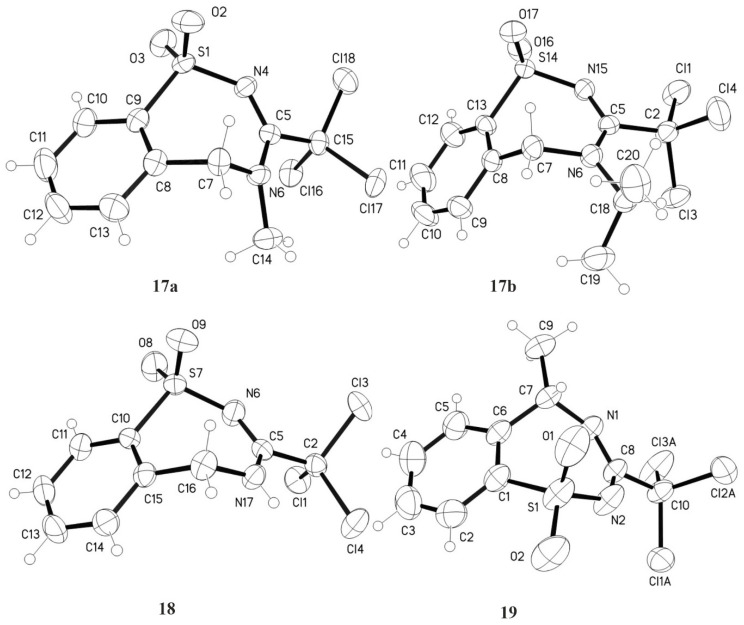
Molecular structure of 4,5-dihydro-1,2,4-benzothiadiazepine-1,1-dioxides **17a**,**b**, **18**, **19** including thermal displacement ellipses with 50% probability.

Molecular structure of benzothiadiazepine-1,1-dioxides **17b, 18,** and **19** was proved by X-ray crystallographic analysis (Figure 6).

**Scheme 8:**
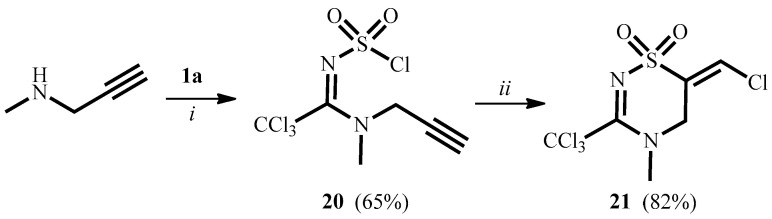
Electrophilic heterocyclization of imidoyl chloride **1a** with *N*-methyl propargylamine. Reagents and conditions: (i) Et_3_N, CH_2_Cl_2_, 0–20 °C, 2 h; (ii) AlCl_3_, CH_2_Cl_2_, r.t., 24 h.

**Figure 7:**
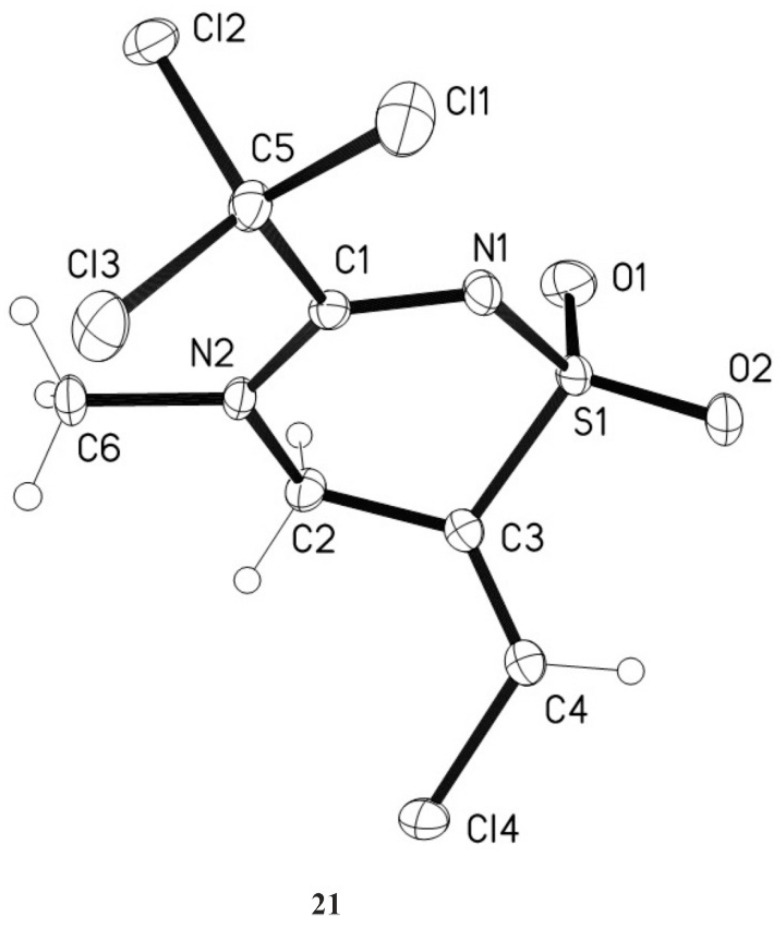
Molecular structure of (*E*)-6-chloromethylene-4-methyl-3-trichloromethyl-5,6-dihydro-4*H*-1,2,4-thiadiazine-1,1-dioxide **21** including thermal displacement ellipses with 50% probability.

Diversity of heterocyclic systems incorporating endocylic sulfonamide fragment, accessible from electrophilic heterocyclizations of imidoyl chloride **1** with alkenylamines, can be enlarged by the use of unsaturated amines with triple carbon-carbon bond. Thus, propargylamine reacts with imidoyl chloride **1a** similarly to allylamine (Scheme 1) with the formation of comparatively stable ynamidine **20** (Scheme 8). Addition of AlCl_3_ initiated clean regio- and stereoselective cyclization to 1,2,4-thiadizine 1,1-dioxides **21** bearing sterically less hindered exocyclic C=C bond of the *E*-configuration. It should be noted that, to the best of our knowledge, there has been no example of sulfonyl group addition to the non-terminal carbon atom of monosubstituted alkynes. Reported in the literature copper [30], iron [31], and iridium [32,33] catalyzed radical sulfonation of alkynes with sulfonyl chlorides takes place at the terminal carbon atom. Regiospecific intramolecular addition to the non-terminal atom of alkyne **20** in Scheme 7 results most likely from beneficial six-member ring formation. The structure of (*E*)-4-chloromethylene 1,2,4-thiadiazine-1,1-dioxide **21** was confirmed by X-ray crystallographic analysis (Figure 7).

**Scheme 9:**
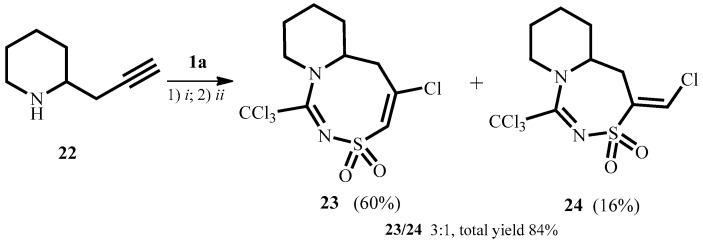
Formation of seven- and eight-membered heterocycles in one-pot electrophilic heterocyclization of imidoyl chloride **1a** with ynamine **22**. Reagents and conditions: (i) Et_3_N, CH_2_Cl_2_, 0–5 °C, 1 h; (ii) AlCl_3_, CH_2_Cl_2_, r.t., 24 h.

**Figure 8:**
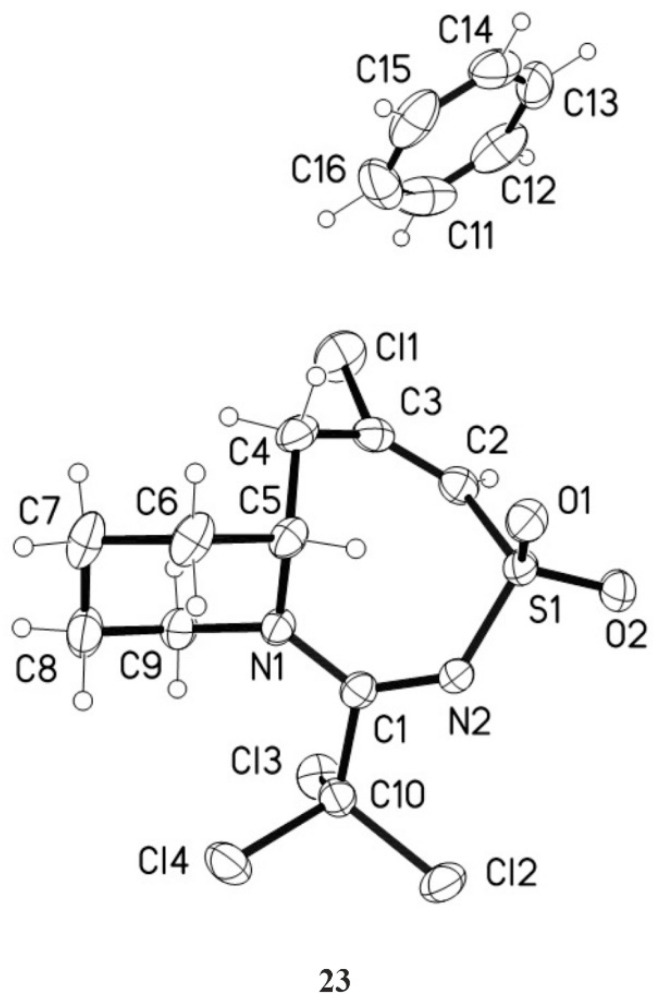
Molecular structure of novel pyrido[1,2-*d*][1,2,4]thiadiazocine heterocyclic system **23** including thermal displacement ellipses with 50% probability.

The introduction of an additional group between C≡C bond and amine nitrogen atom allows preparation of larger sized heterocycles. Thus, the reaction of imidoyl chloride **1a** with ynamine **22** leads to a mixture of pyridothiadiazocine-(**23**) and pyridothiadiazepine 1,1-oxides **24** in a ratio of 3:1 in 84% total yield (Scheme 9). Noteworthy, as in the case of analogous alkenylamines (Scheme 5), formation of eight-membered heterocycle is preferable over seven-membered. (*E*)-Configuration of exocyclic C=C bond in pyridothiadiazepine dioxide **24** was confirmed by NOE experiment: the signal of =CH proton did not show NOE effect with the two multiplets of the 5-CH_2_ group. The similar NOE data were obtained for compound **21**, (*E*)-configuration of which was proved by XRD analysis. Compounds **23** and **24** were separated by chromatography and isolated in 60% and 16% yields, respectively. The structure of the major isomer **23** was proved by X-ray crystallographic analysis (Figure 8).

**Scheme 10:**
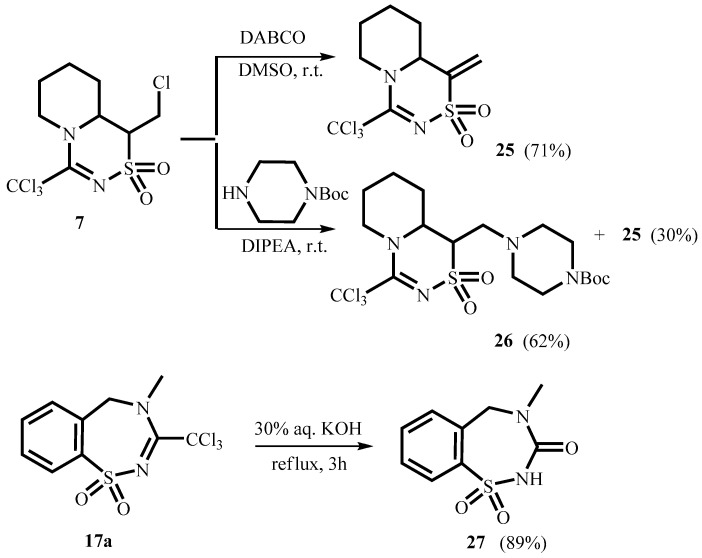
Functionalization of heterocyclic derivatives **7**, **17a**.

**Figure 9:**
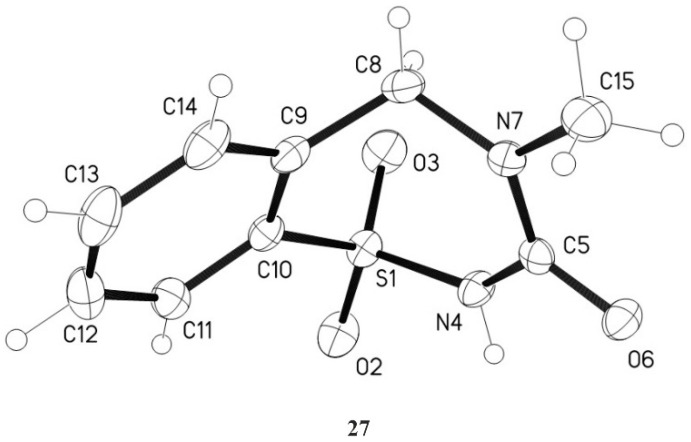
Molecular structure of 4-methyl-4,5-dihydro-1,2,4-benzothiadiazepin-3(2*H*)-one-1,1-dioxide **27** including thermal displacement ellipses with 50% probability.

Synthesized above heterocyclic systems can be further functionalized. Some examples are shown in Scheme 10. Thus, 1-chloromethyl derivative **7** in the presence of base affords methylene derivative **25**. Mild dehydrochlorination is obviously associated with a high acidity of C-H proton in α-position to sulfonyl group. Reaction of **7** with Boc protected piperazine allows preparation of hybrid heterocyclic system **26** combining two potentially bioactive heterocyclic moieties in a single molecular platform. In this case, nucleophilic substitution is accompanied by dehydrochlorination and formation of **25** (30%). On the other hand, CCl_3_C=N moiety of the thiadiazepine 1,1-dioxide **17a** can be readily converted into amide group to afford benzothiadiazepinone-1,1-dioxide **27**. Earlier we have reported the similar haloform cleavage of the benzothiadiazine 1,1-dioxides [24]. The molecular structure of benzothiadiazepinone **27** was unambiguously proven by X-ray crystallographic analysis (Figure 9).

All heterocyclic compounds synthesized are quite stable and can be stored for a long time.

## 3. Materials and Methods

### 3.1. General

^1^H and ^13^C-NMR spectra were acquired on a Varian VXR 400 (Agilent Technologies, Santa Clara, CA, USA), Bruker Avance DRX 500 (Bruker, Ettlingen, Germany) and Agilent 600 (Agilent Technologies, Santa Clara, CA, USA) spectrometers. LCMS analyses were carried out on an Agilent 1200 LC (Agilent Technologies, Santa Clara, CA, USA) system equipped with a G6140 MSD detector (ESI mode). Zorbax C18 RR column (Agilent Technologies, Santa Clara, CA, USA) was used, and gradient elution with 0.1% HCOOH in H2O–MeCN was applied. Preparative HPLC was performed on a Shimadzu LC-8A (Shimadzu Corporation, Tokyo, Japan) equipped with a Phenomenex C18 column (30 × 150 mm) (Phenomenex, Torrance, CA, USA), compounds **23**, **24**, and **26**, or Combiflash RF200 (Teledyne Isco, Lincoln, NE, USA) equipped with a RediSep column (Teledyne Isco, Lincoln, NE, USA),compounds **9** and **10**. Elemental analysis was carried out in the analytical laboratory of Institute of organic chemistry, NAS of Ukraine. Melting points were determined by capillary method. All crystallographic measurements were performed on a Bruker Smart Apex II diffractometer (Bruker, Madison, WI, USA) operating in the ω scans mode. The structures were solved by direct methods and refined by the full-matrix least-squares technique in the anisotropic approximation for non-hydrogen atoms using the Bruker SHELXTL [34] and Crystals [35] program packages. The solvate CHCl_3_ molecule of **4a** could not be modeled satisfactorily thus SQEESE [36] routine in the PlLATON [37,38] software were applied for correction of the data (see Supplementary Materials). 

### 3.2. Typical Procedure for the Synthesis of Compounds ***3a**, **16a**,**b**, **20***

A solution of the appropriate amine **2** (10 mmol) in anhydrous CH_2_Cl_2_ (50 mL) was added dropwise at 0–5 °C to a solution of 2,2,2-trichloro-*N*-(chlorosulfonyl)acetimidoyl chloride **1a** (2.8 g, 10 mmol) in anhydrous CH_2_Cl_2_ (50 mL) for 30 min. Subsequently, a solution of Et_3_N (1.4 mL, 10 mmol) in anhydrous CH_2_Cl_2_ (20 mL) was added dropwise for 30 min and the reaction mixture was stirred at room temperature for 1 h. The solvent was removed under vacuum, the residue was treated with cold water (50 mL), the precipitate was filtered off, washed with water (4 × 30 mL), and air-dried. The crude material was recrystallized from hexane.

#### 3.2.1. 1-(Allyl(methyl)amino)-2,2,2-trichloroethylidenesulfamoyl Chloride **3a**

The title compound was prepared from *N*-methylallylamine **2a** (1.37 g, 19.3 mmol), imidoyl chloride **1a** (5.39 g, 19.3 mmol), and triethylamine (2.7 mL, 19.4 mmol). White solid; Yield 4.12 g (68%); mp 72–73 °C. ^1^H-NMR (CDCl_3_, 500 MHz) δ 3.51 (s, 3H, CH_3_), 4.56 (d, 2H, J 5.5 Hz, CH_2_), 5.46–5.51 (m, 2H, CH_2_), 5.83–5.91 (m, 1H, CH). ^13^C-NMR (CDCl_3_, 125 MHz) δ 42.8, 59.4, 92.4, 122.3, 128.6, 167.8. Anal. calcd for C_6_H_8_Cl_4_N_2_O_2_S, %: C 22.95; H 2.57; Cl 45.16; S 10.21. Found, %: C 22.73; H 2.42; Cl 45.01; S 10.04.

#### 3.2.2. 1-(Benzyl(methyl)amino)-2,2,2-trichloroethylidenesulfamoyl Chloride **16a**

The title compound was prepared from *N*-methylbenzylamine (2.13 g, 17.6 mmol), imidoyl chloride **1a** (4.92 g, 17.6 mmol), and triethylamine (2.5 mL, 17.9 mmol). White solid; Yield 4.87 g (76%); mp 71–72 °C; ^1^H-NMR (CDCl_3_, 500 MHz) δ 3.41 (s, 3H, CH_3_), 5.17 (s, 2H, CH_2_), 7.29–7.36 (m, 2H, Ph), 7.40–7.47 (m, 3H, Ph). ^13^C-NMR (CDCl_3_, 125 MHz) δ 43.4, 61.2, 128.1, 129.3, 129.4, 132.4, 158.7. Anal. calcd for C_10_H_10_Cl_4_N_2_O_2_S, %: C 32.99; H 2.77; Cl 38.95; S 8.81. Found, %: C 32.84; H 2.61; Cl 38.68; S 8.66.

#### 3.2.3. 1-(Benzyl(isopropyl)amino)-2,2,2-trichloroethylidenesulfamoyl Chloride **16b**

The title compound was prepared from *N*-isopropylbenzylamine (1.79 g, 12 mmol), imidoyl chloride **1a** (3.35 g, 12 mmol), triethylamine (1.7 mL, 12.2 mmol). White solid; Yield 3.77 g (80%); mp 93–94 °C; ^1^H-NMR (CDCl_3_, 600 MHz) δ 1.27 (d, 6H, J 6.6 Hz, (CH_3_)_2_), 4.56 (br, 2H, CH_2_), 4.95 (br, 1H, CH), 7.19–7.23 (m, 2H, arom.), 7.29–7.35 (m, 3H, arom.). Some signals of minor conformational isomer were also observed: 1.39 (d, J 6.7 Hz, (CH_3_)_2_), 3.66 (br, CH,), 4.90 (br, CH_2_). ^13^C-NMR (CDCl_3_+DMSO-d_6_, 125 MHz); Major conformational isomer: δ 19.2, 48.2, 55.2, 87.8, 125.7, 127.7, 128.5, 131.8, 159.0; Minor conformational isomer: δ 19.7, 51.5, 51.7, 92.2, 125.8, 127.5, 128.4, 133.6, 151.7. Anal. calcd for C_12_H_14_Cl_4_N_2_O_2_S, %: C 36.76; H 3.60; Cl 36.16; S 8.18. Found, %: C 36.68; H 3.54; Cl 35.85; S 8.06.

#### 3.2.4. 1-(Propargyl(methyl)amino)-2,2,2-trichloroethylidenesulfamoyl Chloride **20**

The title compound was prepared from *N*-methylpropargylamine (1.08 g, 15.7 mmol), imidoyl chloride **1a** (4.39 g, 15.7 mmol), and triethylamine (2.2 mL, 15.8 mmol). White solid; Yield 3.14 g (64%); mp 85–86 °C; ^1^H-NMR (CDCl_3_, 400 MHz) δ 2.57 (t, 1H, *J* 2.4 Hz, CH), 3.66 (s, 3H, CH_3_), 4.71 (d, 2H, *J* 2.4 Hz, CH_2_), ^13^C-NMR (CDCl_3_, 100 MHz) δ 43.5, 47.5, 74.9, 77.5, 93.6, 157.8. Anal. calcd for C_6_H_6_Cl_4_N_2_O_2_S, %: C 23.10; H 1.91; Cl 45.45; S 10.28. Found, %: C 23.15; H 2.12; Cl 45.17; S 10.24.

### 3.3. 6-Chloromethyl-4-methyl-3-trichloromethyl-5,6-dihydro-4H-1,2,4-thiadiazine-1,1-dioxide ***4a***

Anhydrous AlCl_3_ (0.99 g, 7.4 mmol) was slowly added to a solution of the amidine **3a** (2.32 g, 7.4 mmol) in CH_2_Cl_2_ (50 mL), the mixture was stirred at room temperature for 24 h. The solvent was removed under vacuum, the residue was treated with cold water (50 mL), the precipitate was filtered off and dissolved in 10 mL of methanol, the obtained solution was poured into water (200 mL), the precipitate was filtered off, washed with 5% HCl (2 × 20 mL), water (3 × 20 mL) and air-dried. Yield 1.79 g (77%); mp 164–165°C (acetone-toluene 1:4); ^1^H-NMR (acetone-*d*_6_, 600 MHz) δ 3.68 (s, 3H, CH_3_), 3.69 (dd, 1H, *J* 11.5 Hz, 9.8 Hz), 3.64–3.89 (m 1H), 4.10 (dd, 1H, *J* 11.5 Hz, 4.5 Hz), 4.15 (dd, 1H, *J* 15 Hz, 7.9 Hz), 4.33 (dd, 1H, *J* 15 Hz, 4.1 Hz), ^13^C-NMR (DMSO-*d*_6_, 125 MHz) δ 38.6, 42.9, 52.4, 53.3, 92.5, 154.1. Anal. calcd for C_6_H_8_Cl_4_N_2_O_2_S, %: C 22.95; H 2.57; Cl 45.16; S 10.21. Found, %: C 22.73; H 2.69; Cl 45.02; S 9.98.

### 3.4. Typical Procedure for One-Pot Synthesis of Compounds ***4a**–**e**, **7**, **12**, **14***

A solution of the appropriate amine (5 mmol) in anhydrous CH_2_Cl_2_ (40 mL) was added dropwise at 0–5°C to a solution of imidoyl chloride **1a** (1.4 g, 5 mmol) in anhydrous CH_2_Cl_2_ (20 mL) for 30 min. Subsequently, a solution of Et_3_N (0.7 mL, 5 mmol) in anhydrous CH_2_Cl_2_ (10 mL) was added dropwise for 30 min, the mixture was stirred at room temperature for 1 h, then AlCl_3_ (0.67 g, 5 mmol) was added and the obtained mixture was stirred for 24 h at room temperature. The solvent was removed under vacuum, the residue was treated with cold water (50 mL), the precipitate was filtered off and dissolved in 10 mL of methanol, the obtained solution was poured into water (200 mL), the precipitate was filtered off, washed with 5% HCl (2 × 20 mL), water (3 × 20 mL), and air-dried.

#### 3.4.1. Compound **4a**

The title compound was prepared from *N*-methylallylamine **2a** (0.185 g, 2.6 mmol), 2,2,2-trichloro-*N*-(chlorosulfonyl)acetimidoyl chloride **1a** (0.73 g, 2.6 mmol), triethylamine (0.4 mL, 2.9 mmol) and AlCl_3_ (0.35 g, 2.6 mmol). Yield 0.59 g (72%). Physico-chemical characteristics of the obtained compound are identical with the sample obtained from amidine **3a** (Section 3.3).

#### 3.4.2. 6-Chloromethyl-3-trichloromethyl-5,6-dihydro-4H-1,2,4-thiadiazine-1,1-dioxide **4b**

The title compound was prepared from allylamine **2b** (0.28 g, 5 mmol), 2,2,2-trichloro-*N*-(chlorosulfonyl)acetimidoyl chloride **1a** (1.4 g, 5 mmol), triethylamine (0.7 mL, 5 mmol) and AlCl_3_ (0.67 g, 5 mmol). Colorless crystals (CH_3_CN); Yield 1.26 g (84%); mp 200–202°C; ^1^H-NMR (CD_3_CN, 600 MHz) δ 3.62–3.67 (m, 1H), 3.95–3.98 (m, 1H), 4.06–4.10 (m, 2H), 8.14 (br, 1H, NH); ^13^C-NMR (DMSO-d_6_, 125 MHz) δ 38.6, 41.9, 51.2, 91.9, 157.8. Anal. calcd for C_5_H_6_Cl_4_N_2_O_2_S, %: C 20.02; H 2.02; Cl 47.27; S 10.69. Found, %: C 19.90; H 2.08; Cl 46.99; S 10.56.

#### 3.4.3. 6-Chloromethyl-6-methyl-3-trichloromethyl-5,6-dihydro-4H-1,2,4-thiadiazine-1,1-dioxide **4c**

The title compound was prepared from 2-methylprop-2-en-1-yl-amine **2c** (0.41 g, 5.75 mmol), 2,2,2-trichloro-*N*-(chlorosulfonyl)acetimidoyl chloride **1a**, (1.61 g, 5.75 mmol), triethylamine (0.8 mL, 5.75 mmol) and AlCl_3_ (0.77 g, 5.75 mmol). Colorless crystals (CHCl_3_); Yield 1.41 g (78%); mp 184–185°C; ^1^H-NMR (acetone-*d*_6_, 600 MHz) δ 1.73 (s, 3H, CH_3_), 3.51 (d, *J* 14.4 Hz, 1H), 3.68 (d, *J* 14.4 Hz, 1H), 3.94 (d, *J* 11.5 Hz, 1H), 4.16 (d, *J* 11.5 Hz, 1H), 8.74 (br, 1H, NH); ^13^C-NMR (DMSO-*d*_6_, 125 MHz) δ 23.1, 47.9, 49.2, 57.8, 92.7, 156.6. Anal. calcd for C_6_H_8_Cl_4_N_2_O_2_S, %: C 22.95; H 2.57; Cl 45.16; S 10.21. Found, %: C 22.88; H 2.49; Cl 45.13; S 10.09.

#### 3.4.4. 6-Chloromethyl-6-phenyl-3-trichloromethyl-5,6-dihydro-4H-1,2,4-thiadiazine-1,1-dioxide **4d**

The title compound was prepared from 2-phenylprop-2-en-1-yl-amine hydrochloride **2d** (0.78 g, 4.6 mmol), 2,2,2-trichloro-*N*-(chlorosulfonyl)acetimidoyl chloride **1a**, (1.29 g, 4.6 mmol), triethylamine (1.3 mL, 9.3 mmol) and AlCl_3_ (0.62 g, 4.6 mmol). Colorless crystals (CH_3_CN); Yield 1.38 g (80%); mp 202–203 °C; ^1^H-NMR (acetone-*d*_6_, 600 MHz) δ 3.82 (d, *J* 14.7, 1H), 4.23 (d, *J* 14.7, 1H), 4.25 (d, *J* 12.3, 1H), 4.32 (d, *J* 12.3, 1H), 7.36–7.39 (m, 1H, Ph), 7.42–7.44 (m, 2H, Ph), 7.64–7.66 (m, 2H, Ph), 9.12 (br, 1H, NH); ^13^C-NMR (DMSO-*d*_6_, 125 MHz) δ 48.8, 51.0, 64.0, 93.3, 126.3, 128.9, 129.1, 138.1, 157.3. Anal. calcd for C_11_H_10_Cl_4_N_2_O_2_S, %: C 35.13; H 2.68; Cl 37.71; S 8.52. Found, %: C 35.05; H 2.57; Cl 37.59; S 8.34.

#### 3.4.5. 6-Chloromethyl-5,5-dimethyl-3-trichloromethyl-5,6-dihydro-4H-1,2,4-thiadiazine-1,1-dioxide **4e**

The title compound was prepared from 1,1-dimethylprop-2-en-1-yl-amine **2e** (0.62 g, 7.3 mmol), imidoyl chloride **1**a, (2.04 g, 7.3 mmol), triethylamine (1.1 mL, 7.9 mmol) and AlCl_3_ (0.98 g, 7.3 mmol). Colorless crystals (i-PrOH); Yield 1.73 g (72%); mp 173–174 °C; ^1^H-NMR (acetone-*d*_6_, 600 MHz) δ 1.63 (s, 3H, CH_3_), 1.77 (s, 3H, CH_3_), 3.78 (dd, 1H, *J* 5.1 Hz, *J* 4.1 Hz CH), 4.13 (dd, 1H, *J* 12.8 Hz, *J* 4.1 Hz CH_2_), 4.19 (dd, 1H, *J* 12.8 Hz, *J* 5.1 Hz CH_2_), 8.57 (br, 1H, NH), ^13^C-NMR (DMSO-*d*_6_, 125 MHz) δ 23.1, 28.2, 38.5, 58.9, 61.4, 93.1, 155.7. Anal. calcd for C_7_H_10_Cl_4_N_2_O_2_S, %: C, 25.63; H, 3.07; Cl, 43.23; S, 9.77. Found, %: C, 25.49; H, 3.22; Cl, 43.08; S, 9.46.

#### 3.4.6. 1-Chloromethyl-4-trichloromethyl-1,6,7,8,9,9a-hexahydropyrido[1,2-d][1,2,4]thiadiazine-2,2-dioxide **7**

The title compound was prepared from 2-vinylpiperidine hydrochloride **6** (0.6 g, 4.1 mmol), 2,2,2-trichloro-*N*-(chlorosulfonyl)acetimidoyl chloride **1a**, (1.14 g, 4.1 mmol), triethylamine (1.15 mL, 8.25 mmol) and AlCl_3_ (0.55 g, 4.1 mmol). Colorless crystals (benzene); Yield 1.09 g (75%); mp 172–173°C; ^1^H-NMR (CDCl_3_, 600 MHz) δ 1.69–1.77 (m, 1H), 1.78–1.84 (m, 1H), 1.87–1.89 (m, 2H), 2.04–2.07 (m, 1H), 2.48 (ddd, 1H, J 12.2 Hz, J 13.1 Hz, J 3.7 Hz), 3.24–3.29 (m, 1H), 3.32–3.34 (m, 1H), 3.47 (t, 1H, J 11.2 Hz), 4.10–4.14 (m, 2H), 4.92–4.96 (m, 1H). 29 (m, 1H), 3.32–3.34 (m, 1H), 3.47 (t, 1H, J 11.2 Hz), 4.10–4.14 (m, 2H), 4.92–4.96 (m, 1H). ^13^C-NMR (DMSO-d_6_, 100 MHz) δ 25.1 (8-C), 27.0 (7-C), 32.7 (6-C), 41.3 (CH_2_Cl), 55.1 (CH_2_N), 58.2 (CHS), 63.4 (CHN), 93.7 (CCl_3_), 154.9 (C=N). Anal. calcd for C_9_H_12_Cl_4_N_2_O_2_S, %: C 30.53; H 3.42; Cl 40.05; S 9.06. Found, %: C 30.47; H 3.28; Cl 39.76; S 8.92.

#### 3.4.7. 7-Chloro-4-methyl-3-trichloromethyl-5,6,7,8-tetrahydro-4H-1,2,4-thiadiazocine-1,1-dioxide **12**

The title compound was prepared from *N*-methylbut-3-en-1-amine **11** (0.33 g, 3.9 mmol), 2,2,2-trichloro-*N*-(chlorosulfonyl)acetimidoyl chloride **1a**, (1.09 g, 3.9 mmol), triethylamine (0.55 mL, 3.9 mmol) and AlCl_3_ (0.52 g, 3.9 mmol). Colorless crystals (benzene); Yield 0.26 g (20%); mp 174–175 °C; ^1^H-NMR (CDCl_3_, 600 MHz) δ 2.14–2.19 (m, 1H), 2.60–2.65 (m, 1H), 3.51 (s, 3H, CH_3_), 3.66 (dd, 1H, *J* 11.5 Hz, 15.7 Hz), 3.73 (dd, 1H, *J* 8.9 Hz, 15.7 Hz), 4.01 (dt, 1H, *J* 2.3 Hz, 15.4 Hz), 4.18–4.22 (m, 1H), 4.51–4.57 (m, 1H); ^13^C-NMR (DMSO-*d*_6_, 125 MHz) δ 35.7, 42.1, 53.2, 53.4, 60.6, 95.1, 152.8. Anal. calcd for C_7_H_10_Cl_4_N_2_O_2_S, %: C, 25.63; H, 3.07; Cl, 43.23; S, 9.77. Found, %: C, 25.51; H, 3.15; Cl, 43.01; S, 9.54.

#### 3.4.8. 5-Chloro-1-trichloromethyl-4,5,6,6a,7,8,9,10-octahydropyrido[1,2-d][1,2,4]thiadiazocine-3,3-dioxide **14**

The title compound was prepared from 2-allylpiperidine hydrochloride **13** (0.81 g, 5 mmol), 2,2,2-trichloro-*N*-(chlorosulfonyl)acetimidoyl chloride **1a** (1.4 g, 5 mmol), triethylamine (1.4 mL, 10 mmol) and AlCl_3_ (0.67 g, 5 mmol). Colorless crystals (benzene-cyclohexane 1:1); Yield 0.22 g (12%); mp 153–154 °C ^1^H-NMR (CDCl_3_, 600 MHz) δ 1.64–1.76 (m, 3H), 1.79–1.83 (m, 2H), 1.92–1.98 (m, 1H), 2.17 (ddd, 1H, *J* 14.6 Hz, *J* 11.3 Hz, *J* 4 Hz), 2.86–2.91 (m, 1H), 3.19–3.24 (m, 1H), 3.95 (dd, 1H, *J* 13.9 Hz, *J* 11 Hz), 4.16–4.24 (m, 2H), 4.58–4.62 (m, 1H), 5.36–5.40 (m, 1H), ^13^C-NMR (CDCl_3_, 125 MHz) δ 17.8, 24.4, 29.5, 40.5, 47.1, 49.2, 54.3, 65.9, 94.3, 153.0. Anal. calcd for C_10_H_14_Cl_4_N_2_O_2_S, %: C, 32.63; H, 3.83; Cl, 38.52; S, 8.71. Found, %: C, 32.54; H, 3.75; Cl, 38.41; S, 8.52.

### 3.5. 7-Chloro-3-trichloromethyl-1-thia-2,4-diazaspiro[5.5]undec-2-ene-1,1-dioxide 9 and 5a-Chloro-3-trichloromethyl-4,5,5a,6,7,8,9,9a-octahydro-1,2,4-benzothiadiazepine-1,1-dioxide ***10***

A suspension of cyclohex-1-en-1-ylmethylamine hydrochloride **8** (0.56 g, 3.8 mmol) in anhydrous CH_2_Cl_2_ (30 mL) was added at room temperature to a solution of 2,2,2-trichloro-*N*-(chlorosulfonyl)acetimidoyl chloride **1a** (1.06 g, 3.8 mmol) in anhydrous CH_2_Cl_2_ (30 mL). Subsequently, a solution of Et_3_N (1.1 mL, 7.9 mmol) in anhydrous CH_2_Cl_2_ (20 mL) was added dropwise at 0–5 °C for 30 min, the reaction mixture was stirred at room temperature for 1.5 h, then AlCl_3_ (0.51 g, 3.8 mmol) was added and the obtained mixture was stirred for 24 h at room temperature. The solvent was removed under vacuum, the residue was treated with cold water (50 mL), the precipitate was filtered off and dissolved in 5 mL of isopropanol, the solution was poured into cold water (150 mL), the precipitated solid was filtered off, washed with 5% HCl (2 × 20 mL), water (3 × 20 mL) and air-dried. Total yield of isomers **9** and **10** 1.09 g (81%), **9/10** ~ 3:1. Compounds **9** and **10** were separated by preparative HPLC (hexane–*i*-PrOH) performing gradient elution from 3 to 20% of i-PrOH.

#### 3.5.1. 7-Chloro-3-trichloromethyl-1-thia-2,4-diazaspiro[5.5]undec-2-ene-1,1-dioxide **9**

Colorless needles (CH_3_CN); Yield 0.43 g (32%); mp 203–204 °C. ^1^H-NMR (CDCl_3_, 600 MHz) δ 1.63–1.70 (m, 2H), 1.73–1.81 (m, 2H), 1.96–2.04 (m, 2H), 2.09–2.14 (m, 1H), 2.62–2.68 (m, 1H), 3.86 (dd, 1H, *J* 14.5 Hz, J 3.1 Hz), 4.00 (dd, 1H, *J* 14.5 Hz, *J* 3.8 Hz), 4.63–4.65 (m, 1H), 7.08 (br, 1H, NH). ^13^C-NMR (CDCl_3_, 100 MHz) δ 18.9, 20.6, 25.0, 31.1, 50.5, 57.0, 58.6, 91.6, 157.9. Anal. calcd for C_9_H_12_Cl_4_N_2_O_2_S, %: C, 30.53; H, 3.42; Cl, 40.05; S, 9.06. Found, %: C, 30.47; H, 3.31; Cl, 39.81; S, 9.01.

#### 3.5.2. 5a-Chloro-3-trichloromethyl-4,5,5a,6,7,8,9,9a-octahydro-1,2,4-benzothiadiazepine-1,1-dioxide **10**

Colorless prisms (CH_3_CN); Yield 12 mg (9%); mp 223–224 °C; ^1^H-NMR (CDCl_3_, 600 MHz) δ 1.63–1.82 (m, 4H), 1.85–1.88 (m, 1H), 2.04–2.10 (m, 1H), 2.44–2.49 (m, 1H), 2.64–2.68 (m, 1H), 3.63 (dd, 1H, *J* 15.4 Hz, *J* 6.9 Hz), 3.74–3.76 (m, 1H), 4.63 (dd, 1H, *J* 15.4 Hz, *J* 5.1 Hz), 7.23 (br, 1H, NH). ^13^C-NMR (DMSO-*d*_6_, 100 MHz) δ 20.0, 20.6, 22.3, 32.1, 56.5, 64.8, 75.0, 93.1, 158.8. Anal. calcd for C_9_H_12_Cl_4_N_2_O_2_S, %: C, 30.53; H, 3.42; Cl, 40.05; S, 9.06. Found, %: C, 30.50; H, 3.47; Cl, 40.11; S, 8.89.

### 3.6. General Procedure for the Synthesis of Compounds ***17a**,**b***

A solution of the amidine **16** (5 mmol) in anhydrous CH_2_Cl_2_ (50 mL) was treated with AlCl_3_ (0.67 g, 5 mmol) and heated under reflux for 16 h. The solvent was removed in vacuum, the residue was treated with cold water (200 mL), the precipitated solid was filtered off, washed with water (4 × 30 mL), and air-dried. The crude material was recrystallized from acetonitrile.

#### 3.6.1. 4-Methyl-3-trichloromethyl-4,5-dihydro-1,2,4-benzothiadiazepine-1,1-dioxide **17a**

The title compound was prepared from amidine **16a** (2.33 g, 6.4 mmol), and AlCl_3_ (0.86 g, 6.4 mmol). Colorless crystals (CH_3_CN); Yield 1.14 g (54%); mp 202–203 °C (dec.); ^1^H-NMR (CDCl_3_, 500 MHz) δ 3.50 (s, 3H, CH_3_), 4.93 (s, 2H, CH_2_), 7.40 (d, 1H, *J* 7.3 Hz, arom.), 7.61 (dd, 1H, *J* 7.3 Hz, *J* 7.6 Hz, arom.), 7.67 (dd, 1H, *J* 7.3 Hz, *J* 7.6 Hz, arom.), 8.11 (d, 1H, *J* 7.6 Hz, arom.); ^13^C-NMR (DMSO-*d*_6_, 150 MHz) δ 43.3, 55.7, 94.3, 127.2, 130.3, 130.7, 131.5, 134.7, 140.0, 151.9. Anal. calcd for C_10_H_9_Cl_3_N_2_O_2_S, %: C 36.66; H 2.77; N 8.55; Cl 32.46. Found, %: C 36.58; H 2.82; N 8.43; Cl 32.51.

#### 3.6.2. 4-Isopropyl-3-trichloromethyl-4,5-dihydro-1,2,4-benzothiadiazepine-1,1-dioxide **17b**

The title compound was prepared from amidine **16b** (1.78 g, 4.55 mmol) and AlCl_3_ (0.61 g, 4.55 mmol). Colorless crystals (CH_3_CN); Yield 0.76 g (47%); mp 209–210 °C (dec.); ^1^H-NMR (CDCl_3_, 600 MHz) δ 1.40 (d, 6H, *J* 6.6 Hz (CH_3_)_2_), 4.96 (s 2H, CH_2_), 5.08 (sept, *J* 6.6 Hz, 1H, CH), 7.42 (d, 1H, *J* 7.5 Hz, arom.), 7.55 (dd, 1H, *J* 7.5 Hz, J 7.8 Hz, arom.), 7.63 (dd, 1H, *J* 7.5 Hz, *J* 7.8 Hz, arom.), 8.05 (d, 1H, *J* 7.8 Hz, arom.); ^13^C-NMR (DMSO-d_6_, 125 MHz) δ 20.1, 46.3, 55.9, 95.0, 126.5, 130.4, 131.3, 131.9, 134.6, 141.3, 150.6. Anal. calcd for C_12_H_13_Cl_3_N_2_O_2_S, %:C 40.52; H 3.68; N 7.88; Cl 29.90. Found, %: 40.48; H 3.64; N 7.76; Cl 29.84.

### 3.7. General Procedure for the Synthesis of Compounds ***18**, **19***

A solution of benzylamine or (*S*)-1-phenylethylamine (5 mmol) in anhydrous CH_2_Cl_2_ (30 mL) was added dropwise at 0–5 °C to a solution of 2,2,2-trichloro-*N*-(chlorosulfonyl)acetimidoyl chloride **1a** (1.4 g, 5 mmol) in anhydrous CH_2_Cl_2_ (20 mL) for 30 min. Then a solution of Et_3_N (0.7 mL, 5 mmol) in anhydrous CH_2_Cl_2_ (10 mL) was added dropwise for 10 min. The obtained mixture was treated with AlCl_3_ (0.67 g, 5 mmol) and refluxed for 16 h. The solvent was removed in vacuum, the residue was treated with cold water (100 mL). Then, the precipitated solid was filtered off, washed with 5% HCl (2 × 20 mL), water (4 × 20 mL), and air-dried.

#### 3.7.1. 3-Trichloromethyl-4,5-dihydro-1,2,4-benzothiadiazepine-1,1-dioxide **18**

The title compound was prepared from benzylamine (0.6 g, 5.6 mmol), 2,2,2-trichloro-*N*-(chlorosulfonyl)acetimidoyl chloride **1a** (1.56 g, 5.6 mmol), triethylamine (0.8 mL, 5.75 mmol) and AlCl_3_ (0.75 g, 5.6 mmol). Yield 0.88 g (50%); Colorless crystals (CH_3_CN); mp 223–224 °C (dec.). ^1^H-NMR (CDCl_3_+DMSO-*d*_6_, 500 MHz) δ 4.96 (br, 2H, CH_2_), 7.36 (d, 1H, *J* 7.5 Hz, arom.), 7.50 (dd, 1H, *J* 7.5 Hz, *J* 7.8 Hz, arom.), 7.61 (dd, 1H, *J* 7.5 Hz, *J* 7.8 Hz, arom.), 7.92 (d, 1H, *J* 7.8 Hz, arom.), 9.49 (br, 1H, NH); ^13^C-NMR (DMSO-*d*_6_, 125 MHz) δ 42.0, 94.4, 126.8, 130.2, 130.4, 131.3, 135.1, 140.8, 156.5. Anal. calcd for C_9_H_7_Cl_3_N_2_O_2_S, %: C 34.47; H 2.25; N 8.93; Cl 33.92. Found, %: C 34.39; H 2.21; N 8.87; Cl 33.96.

#### 3.7.2. (S)-5-Methyl-3-trichloromethyl-2,3,4,5-tetrahydro-1,2,4-benzothiadiazepine-1,1-dioxide **19**

The title compound was prepared from (*S*)-1-phenylethylamine (0.86 g, 7.15 mmol), 2,2,2-trichloro-*N*-(chlorosulfonyl)acetimidoyl chloride **1a** (2 g, 7.15 mmol), triethylamine (1 mL, 7.2 mmol) and AlCl_3_ (0.95 g, 7.15 mmol). Yield 0.85 g (36%); Colorless crystals (benzene-cyclohexane 4:1); mp 212–213 °C (dec.); [α]_D_^25^ –70.8 (*c* 1.5, CH_3_CN). ^1^H-NMR (CDCl_3_, 600 MHz) δ 1.86 (d, 3H, *J* 6.9 Hz, CH_3_), 6.14 (dq, *J* 6.9 Hz, *J* 3.9 Hz 1H, CH), 6.74 (br, 1H, NH), 7.47 (d, 1H, *J* 7.7 Hz, arom.), 7.58 (t, 1H, *J* 7.7 Hz, arom.), 7.72 (t, 1H, *J* 7.7 Hz, arom.), 8.11 (d, 1H, *J* 7.7 Hz, arom.). ^13^C-NMR (CDCl_3_, 150 MHz) δ 17.1, 49.0, 94.0, 124.4, 128.2, 129.8, 134.3, 139.5, 155.0. Anal. calcd for C_10_H_9_Cl_3_N_2_O_2_S, %: C 36.66; H 2.77; N 8.55; Cl 32.46. Found, %: C 36.55; H 2.85; N 8.47; Cl 32.42.

### 3.8. (E)-6-Chloromethylene-4-methyl-3-trichloromethyl-5,6-dihydro-4H-1,2,4-thiadiazine-1,1-dioxide ***21***

To a solution of amidine **20** (1.34 g, 4.3 mmol) in anhydrous CH_2_Cl_2_ (50 mL) was added AlCl_3_ (0.58 g, 4.3 mmol) and the mixture was stirred for 24 h at room temperature. The solvent was removed in vacuum, the residue was treated with cold water (100 mL), the precipitate was filtered off and dissolved in 5 mL of methanol, the solution was poured into cold water (200 mL), the precipitated solid was filtered off, washed with water (3 × 30 mL) and air-dried. Yield 0.96 g (71%); Colorless crystals (benzene); mp 173–174 °C; ^1^H-NMR (CDCl_3_, 400 MHz) δ 3.61 (s, 3H, CH_3_), 4.63 (d, 2H, *J* 1.3 Hz, CH_2_), 7.33 (t, 1H, *J* 1.3 Hz, CH), ^13^C-NMR (DMSO-*d*_6_, 100 MHz) δ 42.5, 51.8, 92.2, 129.4, 130.2, 154.7. Anal. calcd for C_6_H_6_Cl_4_N_2_O_2_S, %: C 23.10; H 1.91; Cl 45.45; S 10.28. Found, %: C 23.12; H 1.84; Cl 45.36; S 10.19.

### 3.9. 5-Chloro-1-trichloromethyl-6,6a,7,8,9,10-hexahydropyrido[1,2-d][1,2,4]thiadiazocine 3,3-dioxide 23 and (E)-4-Chloromethylene-1-trichloromethyl-5,5a,6,7,8,9-hexahydro-4H-pyrido[1,2-d][1,2,4]thiadiazepine-3,3-dioxide ***24***

A solution of Et_3_N (1.13 ml, 8.05 mmol) in anhydrous CH_2_Cl_2_ (40 mL) was added dropwise to a mixture of 2-prop-2-yn-1-ylpiperidine hydrochloride **22** (0.64 g, 4 mmol) and 2,2,2-trichloro-*N*-(chlorosulfonyl)acetimidoyl chloride **1a** (1.12 g, 4 mmol) in anhydrous CH_2_Cl_2_ (60 mL) at 0–5 °C. After 1 h, AlCl_3_ (0.54 g, 4 mmol) was added and the mixture was stirred for 24 h at room temperature. The solvent was removed under vacuum, the residue was treated with cold water (100 mL), the precipitate was filtered off and dissolved in 10 mL of isopropanol, the obtained solution was poured into water (200 mL), the precipitated solid was filtered off, washed with 5% HCl (2 × 20 mL), water (3 × 20 mL) and air-dried. Total yield of isomers **23** and **24** 1.23 g (84%), **23**/**24** ~3:1. Individual compounds **23** and **24** were isolated by preparative HPLC (CH_3_CN–H_2_O) performing gradient elution from 50 to 100% of CH_3_CN.

#### 3.9.1. 5-Chloro-1-trichloromethyl-6,6a,7,8,9,10-hexahydropyrido[1,2-d][1,2,4]thiadiazocine 3,3-dioxide **23**

White solid; Yield 0.88 g (60%); mp 152–153 °C. ^1^H-NMR (CDCl_3_, 600 MHz) δ 1.69–1.88 (m, 4H), 1.93–2.04 (m, 1H), 3.21–3.30 (m, 2H), 3.36–3.42 (m, 1H), 4.50–4.56 (m, 1H), 5.49–5.57 (m, 1H), 6.75 (d, 1H, *J* 2.4 Hz), ^13^C-NMR (CDCl_3_, 100 MHz) δ 17.1, 24.5, 29.1, 39.8, 46.8, 56.6, 94.2, 128.2, 148.5, 157.5. Anal. calcd for C_10_H_12_Cl_4_N_2_O_2_S, %: C, 32.81; H, 3.30; Cl, 38.74; S, 8.76. Found, %: C, 32.73; H, 3.37; Cl, 38.65; S, 8.62. Single crystals for XRD analysis were obtained by recrystallization from benzene.

#### 3.9.2. (E)-4-Chloromethylene-1-(trichloromethyl)-5,5a,6,7,8,9-hexahydro-4H-pyrido[1,2-d][1,2,4]thiadiazepine-3,3-dioxide **24**

White solid; Yield 0.23 g (16%); mp 124–125 °C. ^1^H-NMR (CDCl_3_, 600 MHz) δ 1.69–1.84 (m, 5H), 1.91–1.97 (m, 1H), 15.6 (ddd, 1H, *J* 15.6 Hz, *J* 4.4 Hz, *J* 2.6 Hz), 3.09–3.13 (m, 1H), 3.69 (dd, 1H, *J* 13.6 Hz, 15 Hz), 4.25–4.29 (m, 1H), 4.76–4.81 (m, 1H), 7.41 (d, 1H, *J* 2.6 Hz), ^13^C-NMR (CDCl_3_, 100 MHz) δ 18.2, 24.3, 28.6, 31.7, 47.1, 55.6, 93.9, 132.2, 138.1, 159.2. Anal. calcd for C_10_H_12_Cl_4_N_2_O_2_S, %: C, 32.81; H, 3.30; Cl, 38.74; S, 8.76. Found, %: C, 32.77; H, 3.39; Cl, 38.53; S, 8.60. Single crystals for XRD analysis were obtained by recrystallization from acetone-cyclohexane (1:2).

### 3.10. 1-Methylene-4-(trichloromethyl)-1,6,7,8,9,9a-hexahydropyrido[1,2-d][1,2,4]thiadiazine-2,2-dioxide ***25***

A solution of DABCO (47 mg, 0.42 mmol) in DMSO (2 mL) was added to a solution of **7** (142 mg, 0.4 mmol) in DMSO (3 mL) and the obtained mixture was stirred at room temperature overnight. The solution was poured into cold water (100 mL), the precipitated solid was filtered off, washed with water (4 × 10 mL) and air-dried. White solid; Yield 91 mg (71%); mp 173–174 °C. ^1^H-NMR (CDCl_3_, 600 MHz) δ 1.65–1.73 (m, 1H), 1.75–1.87 (m, 2H), 2.00–2.08 (m, 2H), 2.37 (ddd, 1H, *J* 25.7 Hz, *J* 12.7 Hz, *J* 3.6 Hz), 3.18–3.22 (m, 1H), 4.40 (d, 1H, *J* 12.1 Hz), 4.86–4.90 (m, 1H), 5.82 (s, 1H), 6.27 (s, 1H). ^13^C-NMR (CDCl_3_, 100 MHz) δ 23.7, 25.5, 32.3, 53.6, 65.5, 93.3, 122.4, 140.0, 155.0. Anal. calcd for C_9_H_11_Cl_3_N_2_O_2_S, %: C 34.03; H 3.49; N 8.82; Cl 33.49. Found, %: C 33.72; H 3.62; N 8.63; Cl 33.24.

### 3.11. 1-(4-N-Boc-piperazin-1-yl)methyl-4-trichloromethyl-1,6,7,8,9,9a-hexahydropyrido[1,2-d][1,2,4]thiadiazine-2,2-dioxide ***26***

*N*-Boc-piperazine (125 mg, 0.67 mmol) was added to a solution of the compound **7** (237 mg, 0.67 mmol) in DMSO (4 mL). Subsequently, a solution of DIPEA (86 mg, 0.67 mmol) in DMSO (2 mL) was added and the obtained mixture was stirred overnight at room temperature. The solution was poured into cold water (150 mL) the precipitated solid was filtered off, washed with water (5 × 10 mL) and air-dried to yield 315 mg of **26** containing 30% of dehydrochlorination product **25** (^1^H-NMR). Compound **26** was isolated by preparative HPLC (CH_3_CN–H_2_O) performing gradient elution from 50 to 100% of CH_3_CN. White solid; Yield 210 mg (62%); mp 112–113 °C. ^1^H-NMR (CDCl_3_, 600 MHz) δ 1.44 (s, 9H), 1.58–1.71 (br, 2H), 1.76–1.86 (m, 2H), 1.83–1.95 (br, 1H), 1.99–2.06 (m, 1H), 2.18–2.26 (m, 1H), 2.30–2.46 (br, 3H), 2.47–2.61 (br, 2H), 2.92–3.05 (br, 1H), 3.11–3.30 (br, 2H), 3.33–3.61 (br, 4H), 3.82–3.96 (br, 1H), 4.04–4.17 (br, 1H), 4.86–4.91 (m, 1H). ^13^C-NMR (CDCl_3_, 100 MHz) δ 24.3 (8-C), 26.1 (7-C), 28.4 (Me), 31.8 (6-C), 43.5 (br, CH_2_NCO), 53.4 (2CH_2_N-piperazine), 54.0 (C-5), 54.4 (CH_2_CHS), 55.8 (C-1), 63.3 (CHN), 80.0 (CMe3), 93.3 (CCl_3_), 154.63, 154.7 (C=N, C=O) Some low intensity signals of minor conformational isomer, caused by restricted rotation around amide N-C bond, were also observed: 25.4, 26.3, 50.7, 52.2, 53.2, 63,4, 93.4, 153.9, 154.62 ppm. Anal. calcd for C_18_H_29_Cl_3_N_4_O_4_S, %: C 42.91; H 5.80; N 11.12; Cl 21.11. Found, %: C 42.62; H 5.89; N 10.93; Cl 20.94.

### 3.12. 4-Methyl-4,5-dihydro-1,2,4-benzothiadiazepin-3(2H)-one-1,1-dioxide ***27***

The mixture of 0.65 g (2 mmol) of compound **17a** and 30 mL of 30% aq. KOH solution was refluxed for 3 h until complete dissolution of the precipitate. Concentrated HCl (60 mL) was added to ice cooled solution, the precipitated solid was filtered off, washed with water (4 × 20 mL), and air-dried. Colorless crystals (CH_3_CN); Yield 0.3 g (66%); mp 208–209 °C; ^1^H-NMR (DMSO-*d*_6_, 400 MHz) δ 3.00 (s, 3H, CH_3_), 4.78 (s, 2H, CH_2_), 7.60–7.63 (m, 2H, arom.), 7.67–7.71 (m, 1H, arom.), 7.82–7.84 (m, 1H, arom.), 11.31 (br, 1H, NH). ^13^C-NMR (DMSO-*d*_6_, 125 MHz) δ 37.9, 52.2, 124.9, 129.6, 130.5, 133.6, 133.7, 140.2, 152.9. Anal. calcd for C_9_H_10_N_2_O_3_S, %: C 47.78; H 4.45; N 12.38; S 14.17. Found %: C 47.64; H 4.36; N 12.26; S 13.88.

## 4. Conclusions

In conclusion, a new type of intramolecular electrophilic heterocyclization involving sulfonyl chloride as electrophilic site and C=C or C≡C bond as a nucleophilic site was found. Aluminum chloride initiated electrophilic heterocyclization of unsaturated amidines generated from imidoyl chloride **1a** and various amines bearing double or triple carbon-carbon bond allows preparation of six-, seven- and eight-numbered monocyclic and fused heterocycles with biorelevant endocyclic sulfonamide moiety. Four new heterocyclic ring systems were synthesized: derivatives of the pyrido[1,2-*d*][1,2,4]thiadiazine **7**, **25**, **26**, 1,2,4-thiadiazocine **12**, **14**, **23**, and pyrido[1,2-*d*][1,2,4]thiadiazepine **24**. The structures of the heterocyles synthesized was unambigously proved by X-ray crystallographic analysis. The regularities found for the regiochemistry of the new heterocyclization may be used for the purposeful preparation of 6-, 7- and 8-numbered heterocycles with biorelevant sulfonamide fragment.

## Figures and Tables

**Table 1:** Intramolecular heterocyclization of **3a**.

Entry	Reagent and Conditions	Conversion of 3a to 4a (%) ^a^
1	ClCH_2_CH_2_Cl, reflux, 7 h	NR ^b^
2	AlCl_3_, CH_2_Cl_2_, r.t, 24 h	100
3	TiCl_4_, CH_2_Cl_2_, r.t, 24 h	100
4	BF_3_·Et_2_O, CH_2_Cl_2_, r.t, 24 h	NR ^b^
5	ZnCl_2_, CH_2_Cl_2_, r.t, 24 h	NR ^b^

^a^ Determined by LCMS; ^b^ **4a** not detected by LCMS.

## Supplementary Materials

The following are available online. ^1^H and ^13^C-NMR data of all the new compounds, crystallographic XRD data for compounds **4a**,**b**,**d**, **7**, **10**, **12**, **14**, **17a**,**b**, **18**, **19**, **21**, **23**, **27**. Refs. [34,35,36,37,38] are cited in the Supplementary Materials.
