# Supplementary material for: Novel Convenient Approach to 6-, 7-, and 8-Numbered Nitrogen Heterocycles Incorporating Endocyclic Sulfonamide Fragment
Source: Molecules. 2020 Jun 23;25(12):2887. doi: 10.3390/molecules25122887 (PMC7356088; doi:10.3390/molecules25122887)
Supplement: Supplementary file 1 [file molecules-25-02887-s001.pdf]

Supplementary material

# Novel Convenient Approach to 6-, 7-, and 8-Numbered Nitrogen Heterocycles Incorporating Endocyclic Sulfonamide Fragment

Oleksandr Shalimov[1], Eduard Rusanov[1], Oksana Muzychka[2], Petro Onys'ko[1,*]

[1] Department of Heteroatom Chemistry, Institute of Organic Chemistry, National Academy of Sciences of Ukraine, 02660 Kyiv, 5 Murmans'ka, Ukraine; onysko_@ukr.net (P.O.); ashal@ukr.net (O.S.); xray@ioch.kiev.ua (E.R.)

[2] Department of Mechanisms of Bioorganic Reactions, V.P. Kukhar Institute of Bioorganic Chemistry and Petrochemistry of the National Academy of Sciences of Ukraine, 1 Murmanska St., Kyiv 02094, Ukraine; oksana@bpci.kiev.ua

* Correspondence: onysko_@ukr.net (P.O.); Tel.: +38 044 573 25 94

**3a**

$^{1}$H-NMR ($CDCl_3$, 500 MHz)

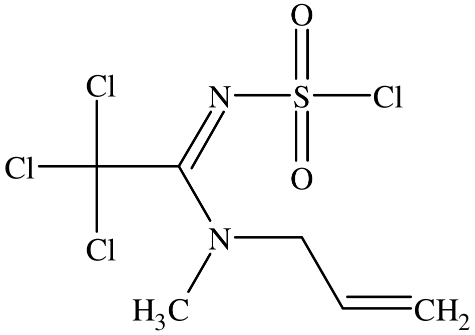


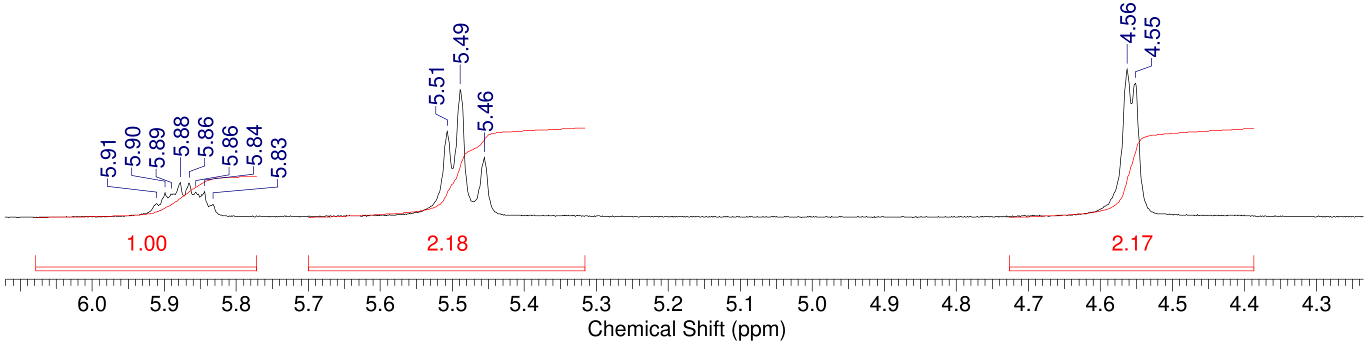


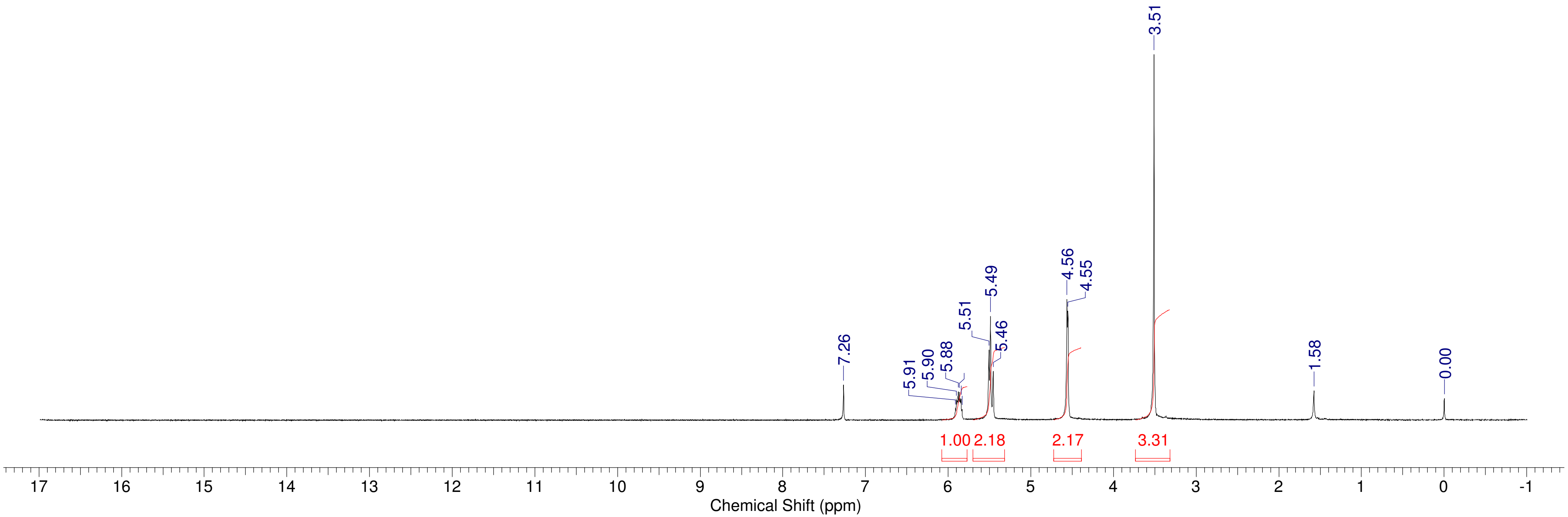

**3a** $^{13}$C-NMR (CDCl$_3$, 125 MHz)

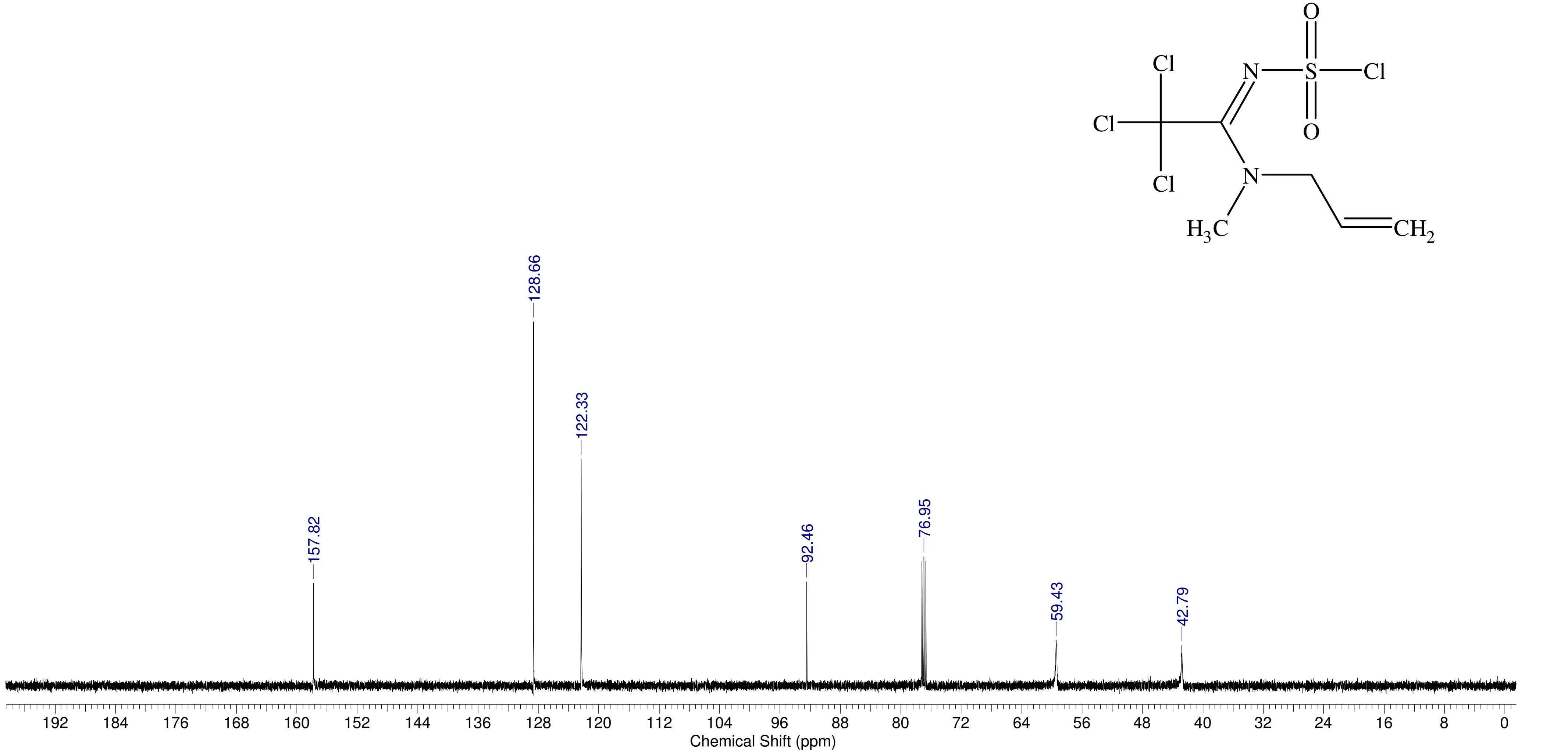

**4a** $^{1}$H-NMR (acetone-d6, 600 MHz)

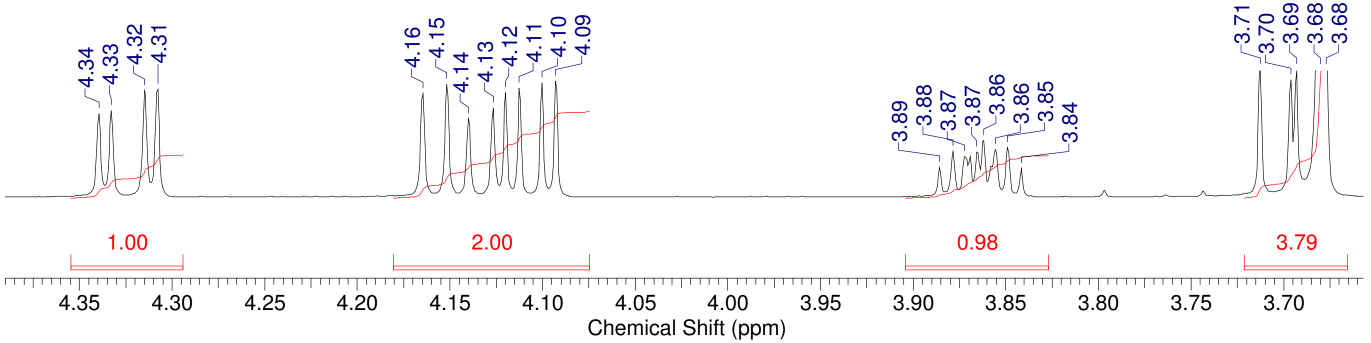


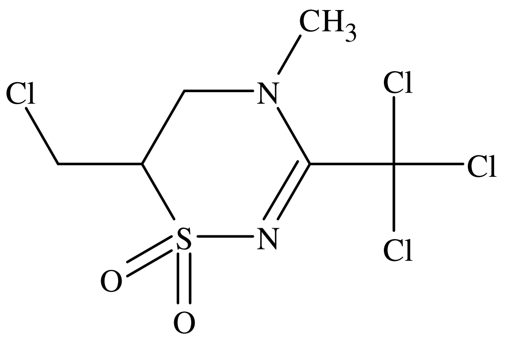


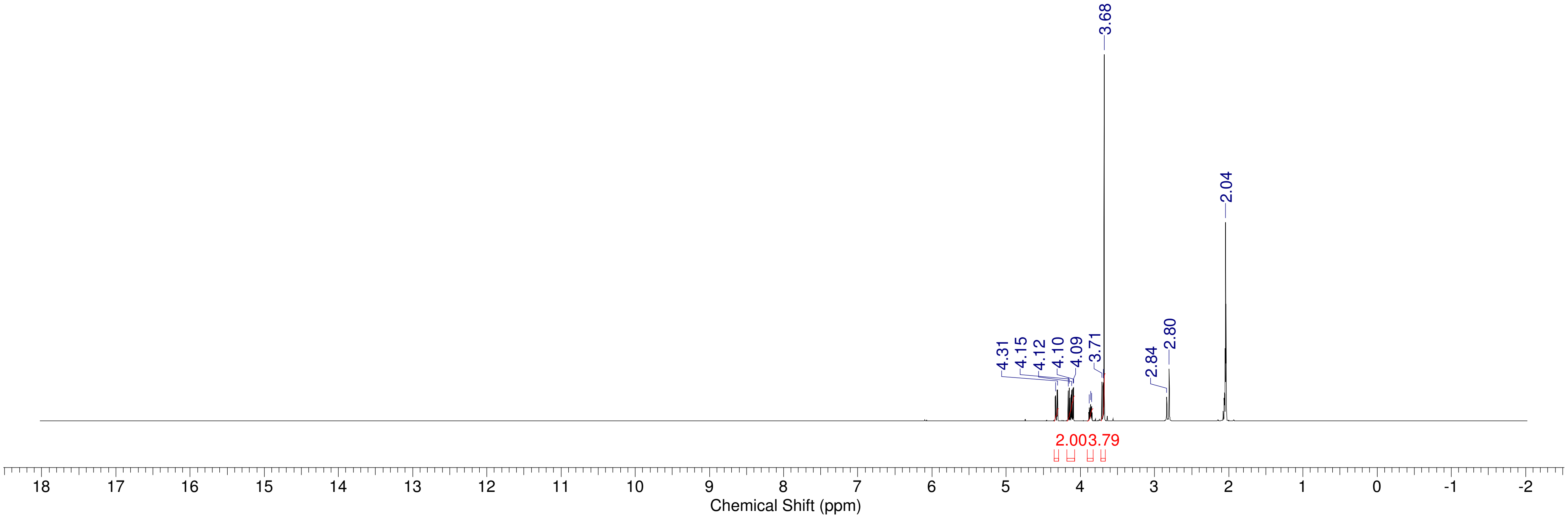

**4a** $^{13}$C-NMR (DMSO-d6, 125 MHz)

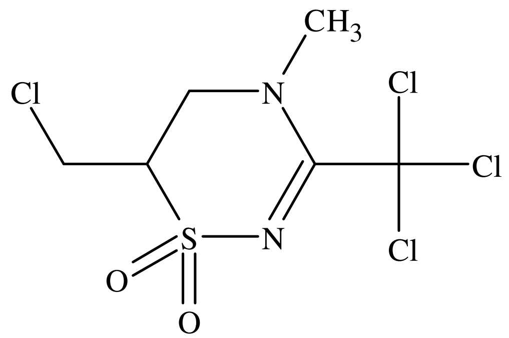


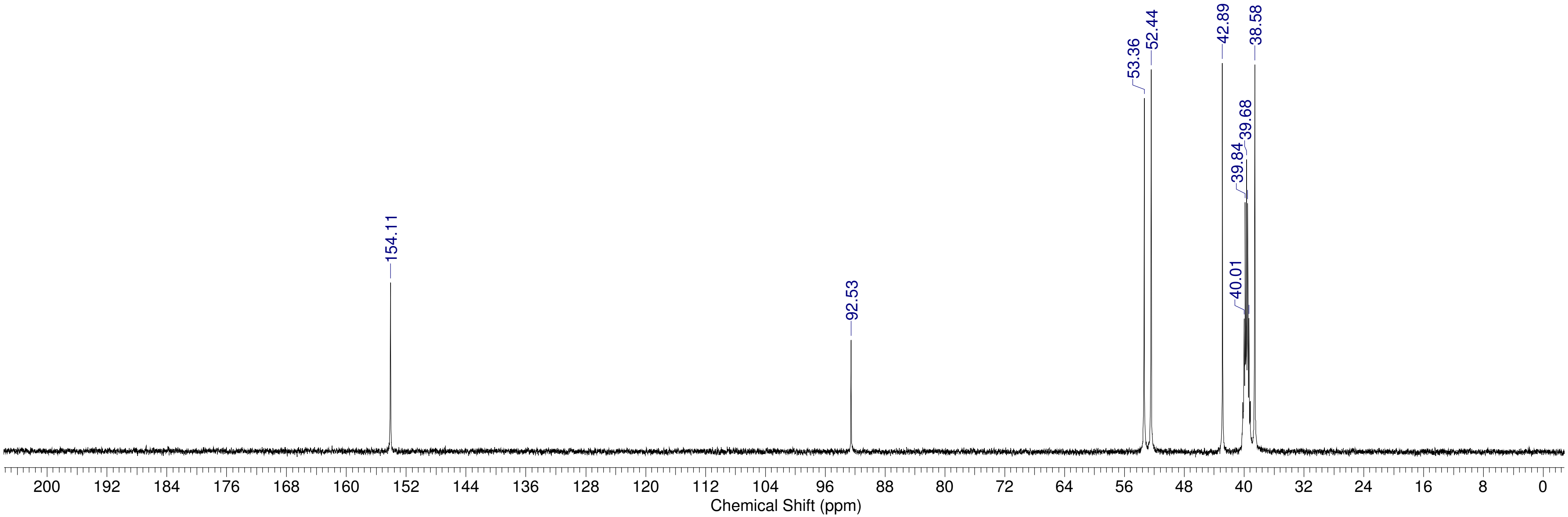

**4b** $^1$H-NMR ($CD_3CN$, 600 MHz)

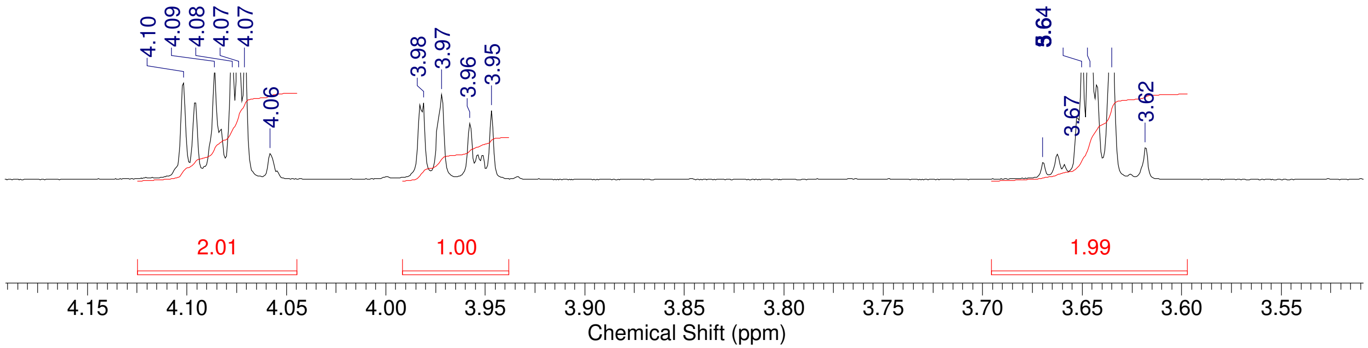


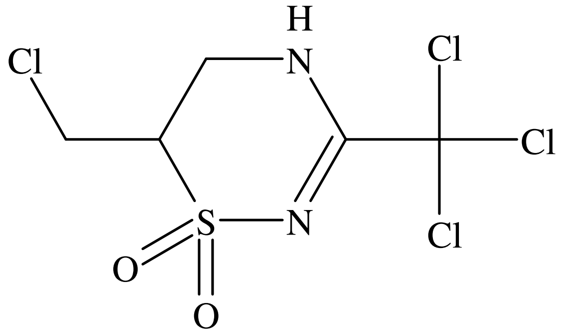


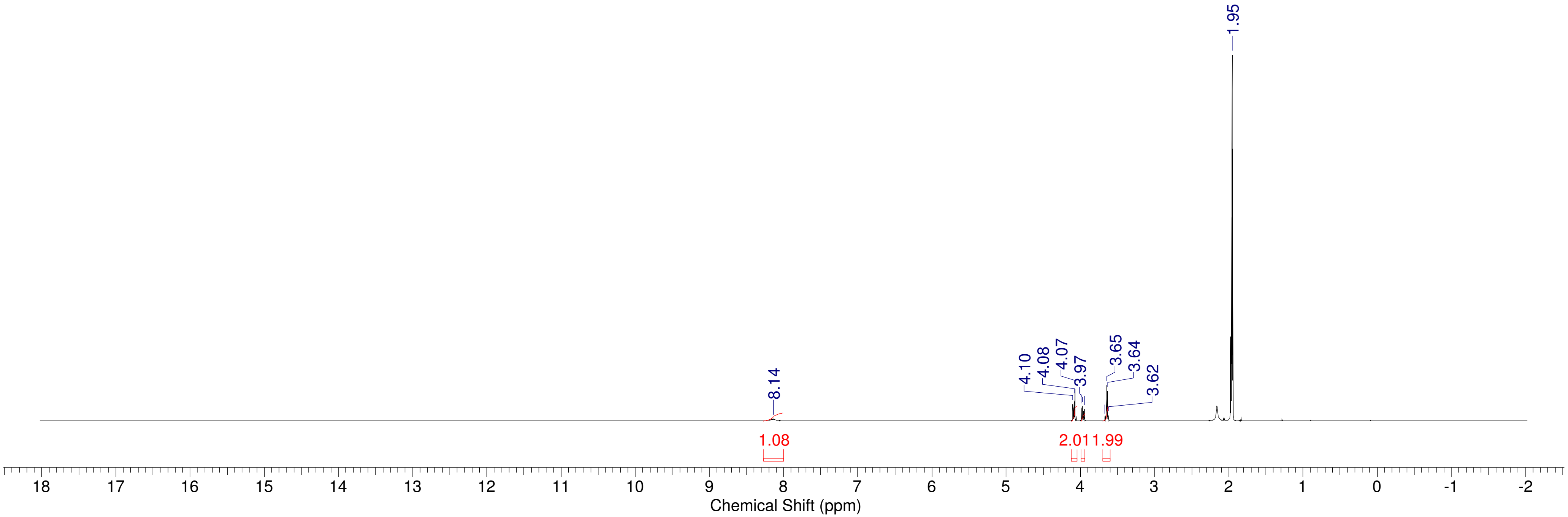

**4b** $^{13}$C-NMR (DMSO-d6, 125 MHz)

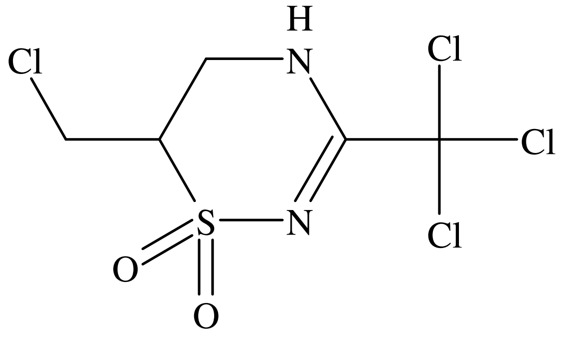


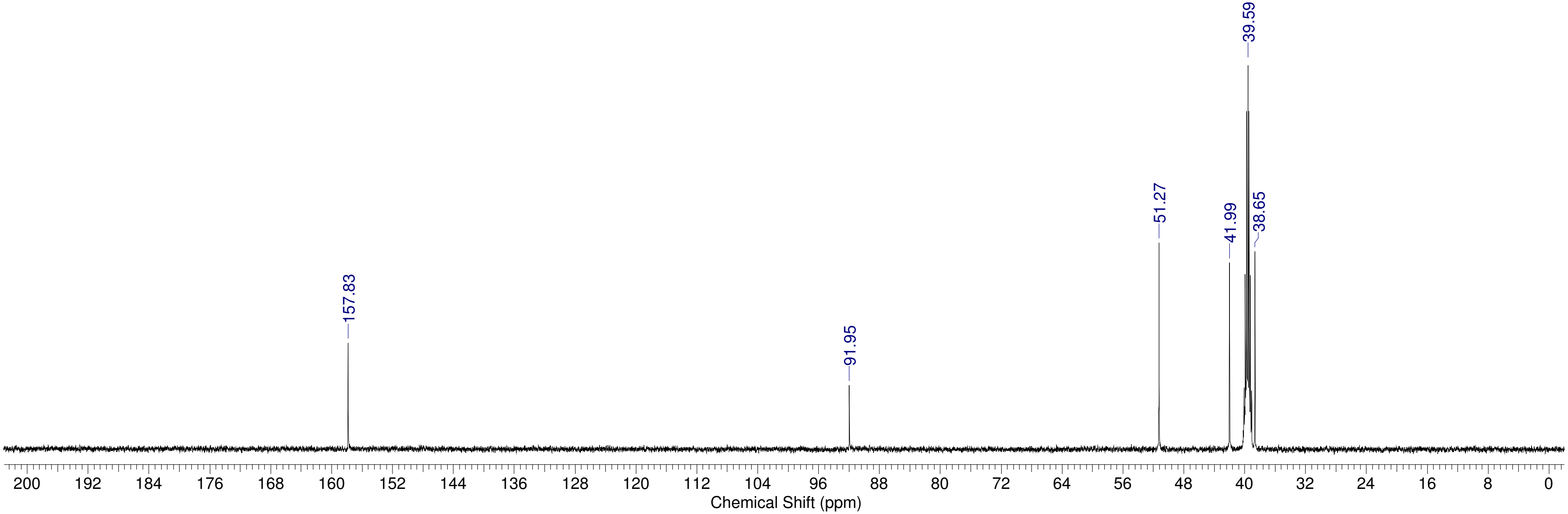

**4c** $^1$H-NMR (acetone-d6, 600 MHz)

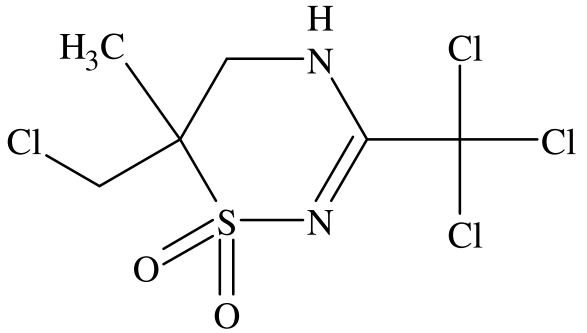


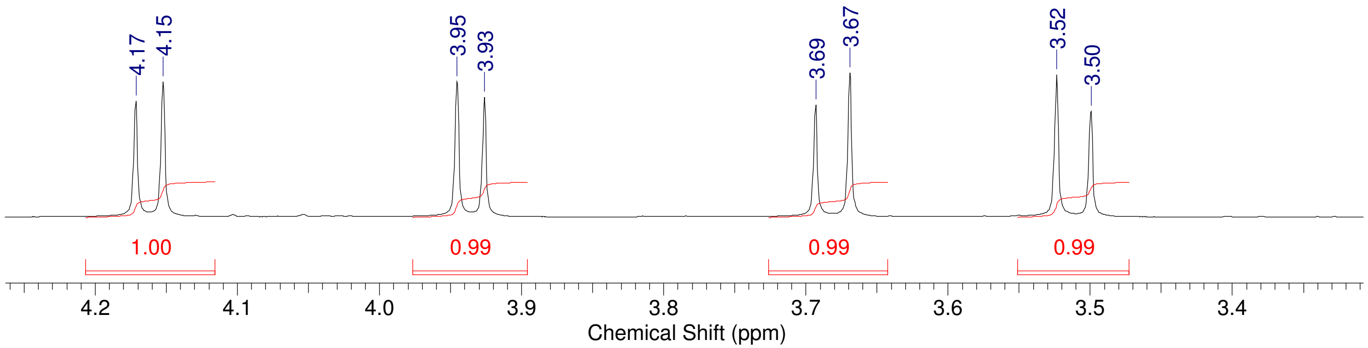


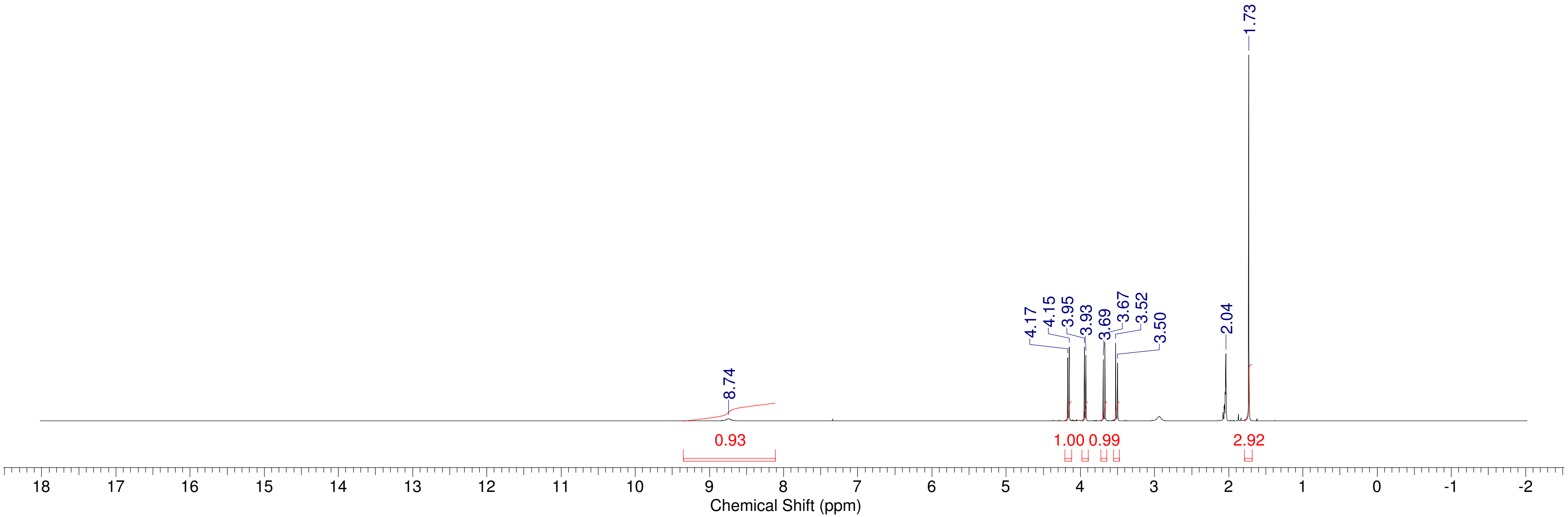

**4c** $^{13}$C-NMR (DMSO-d6, 125 MHz)

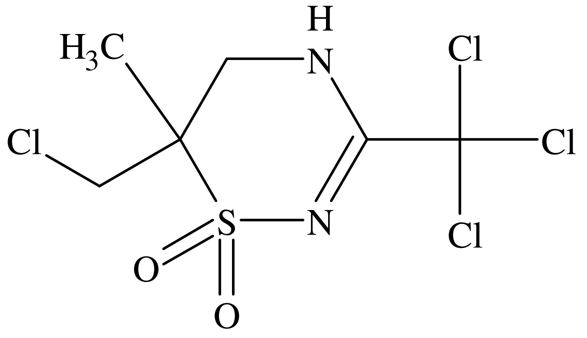

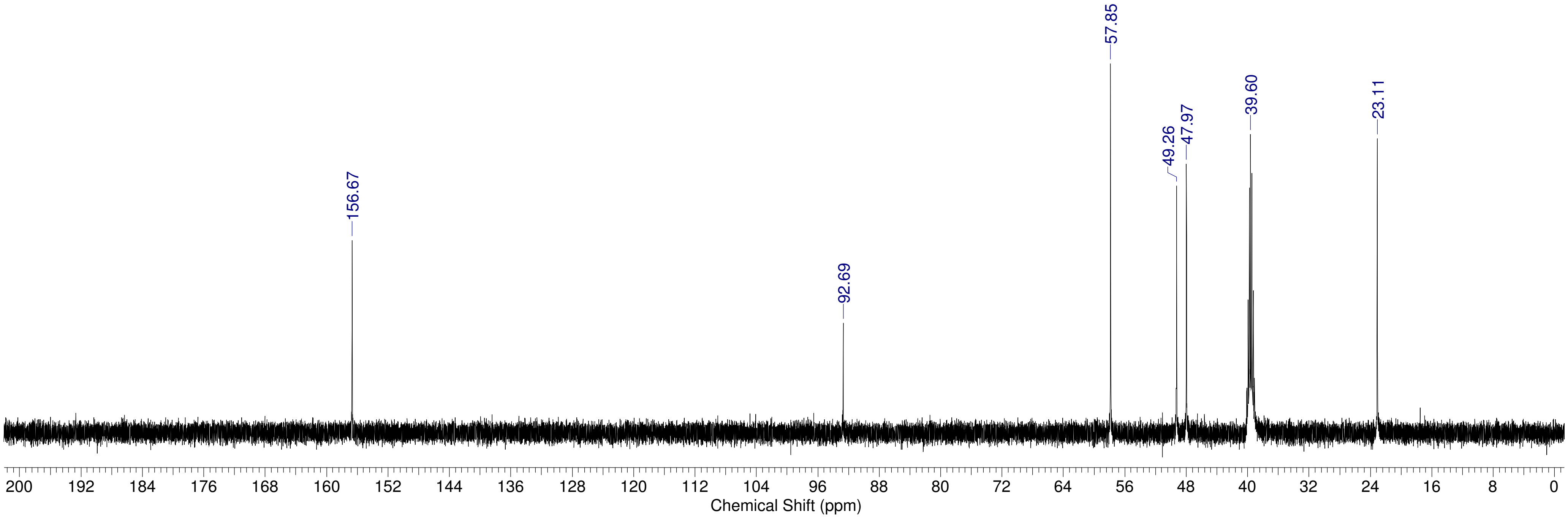

**4d** $^{1}$H-NMR (acetone-d6, 600 MHz)

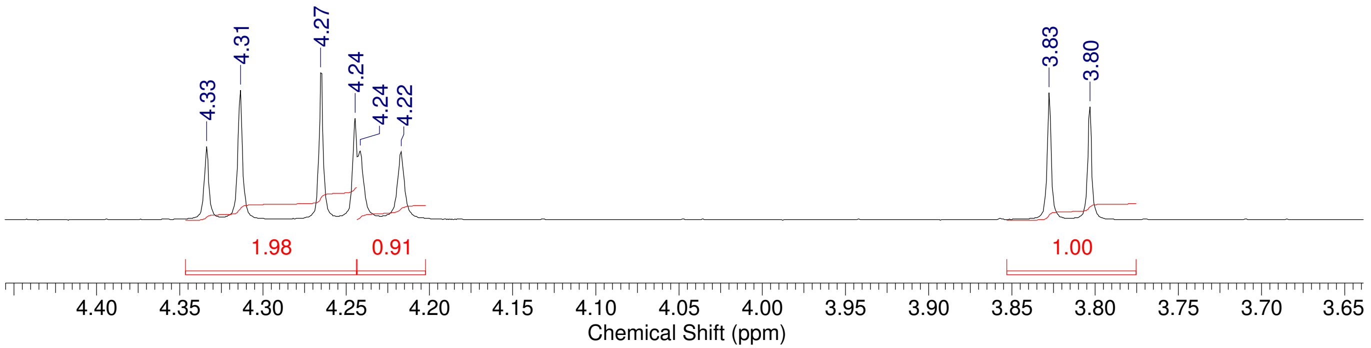


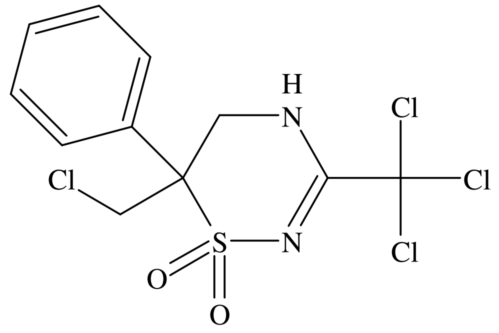


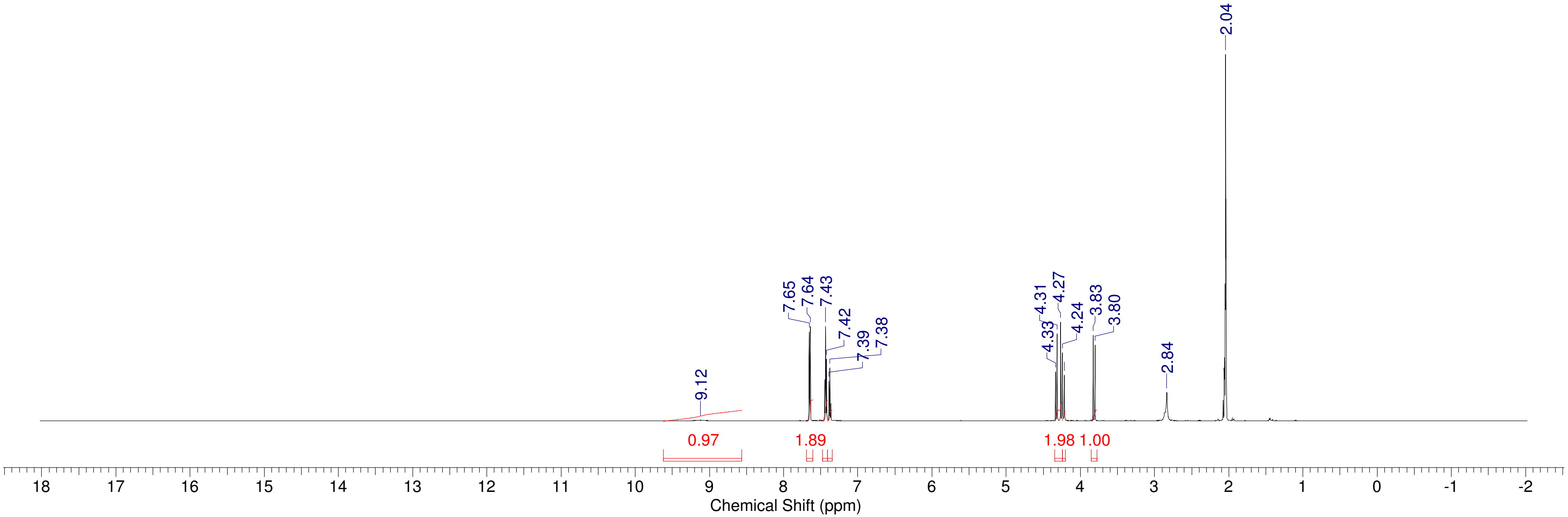

**4d** $^{13}$C-NMR (DMSO-d6, 125 MHz)

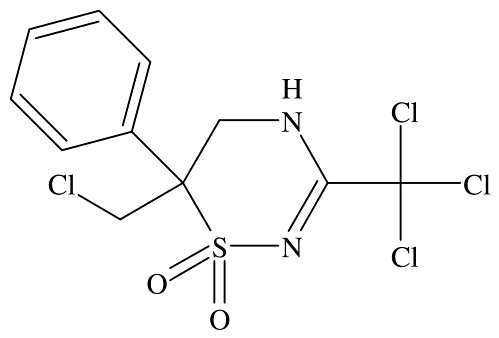


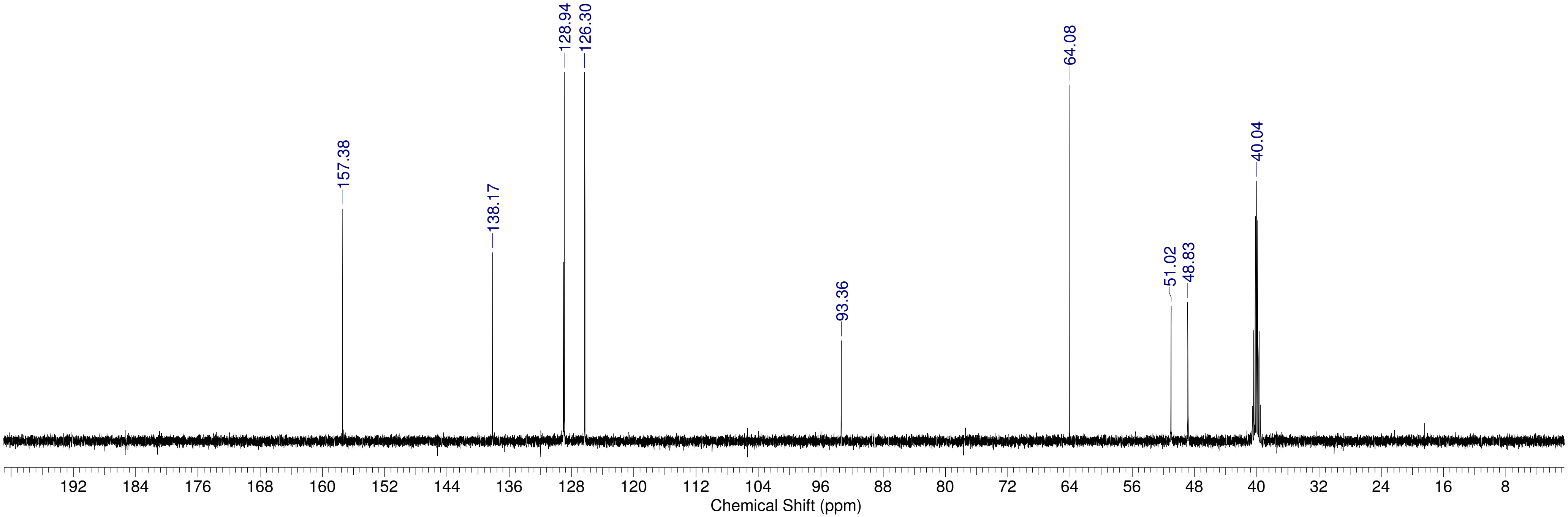

**4e** $^{1}$H-NMR (acetone-d6, 600 MHz)

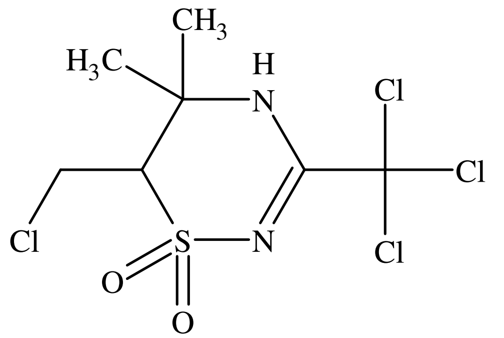


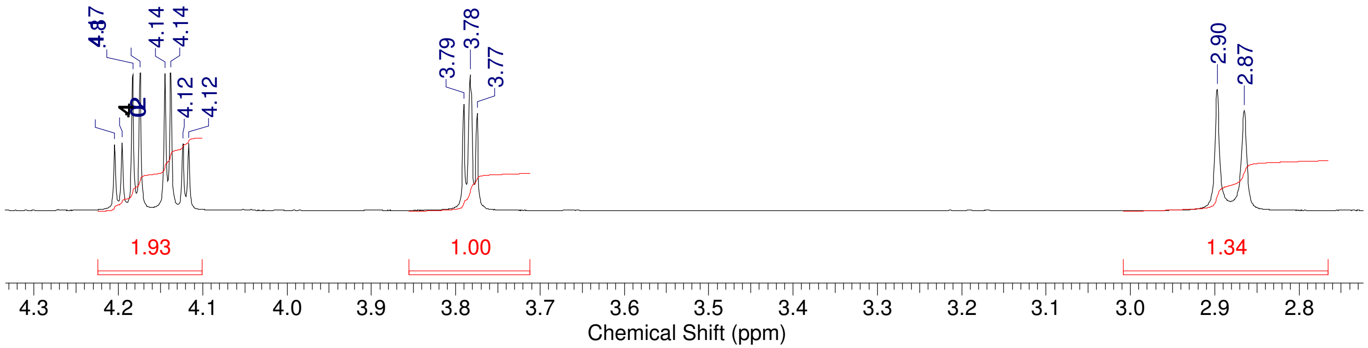


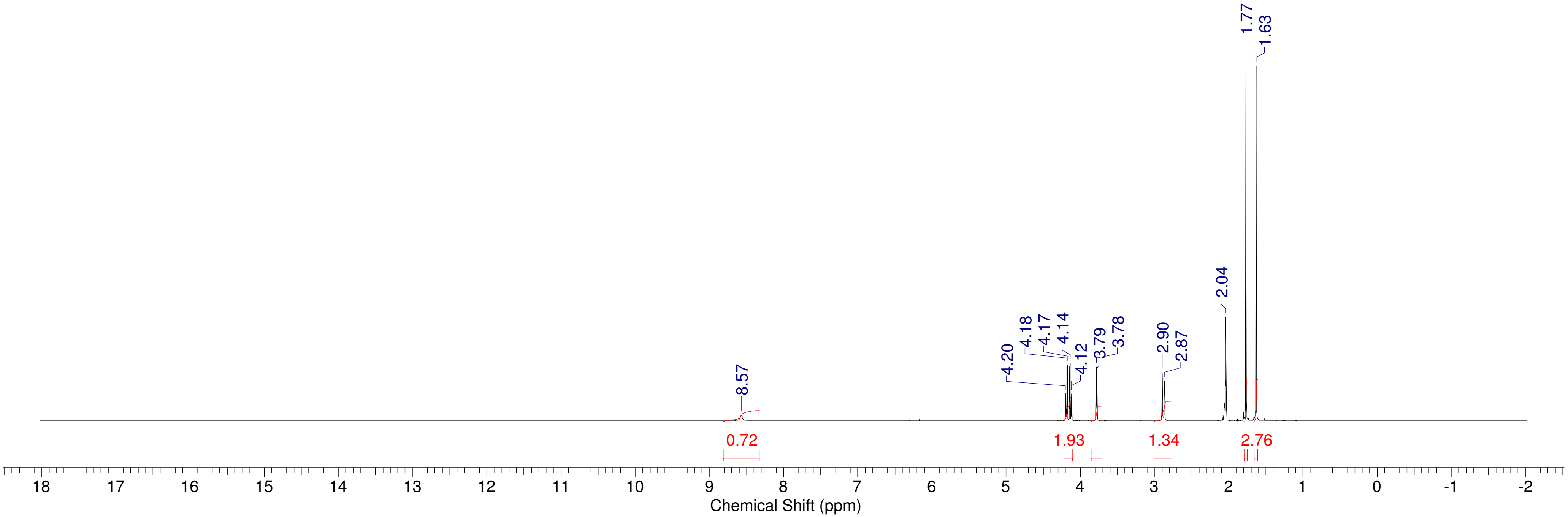

**4e** $^{13}$C-NMR (DMSO-d6, 125 MHz)

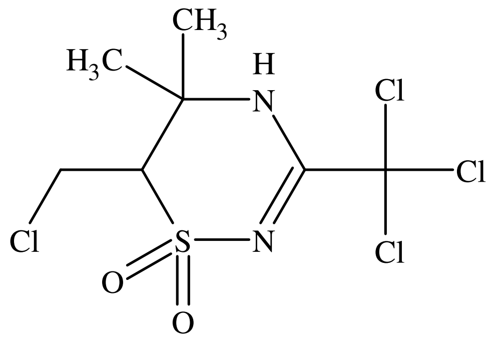

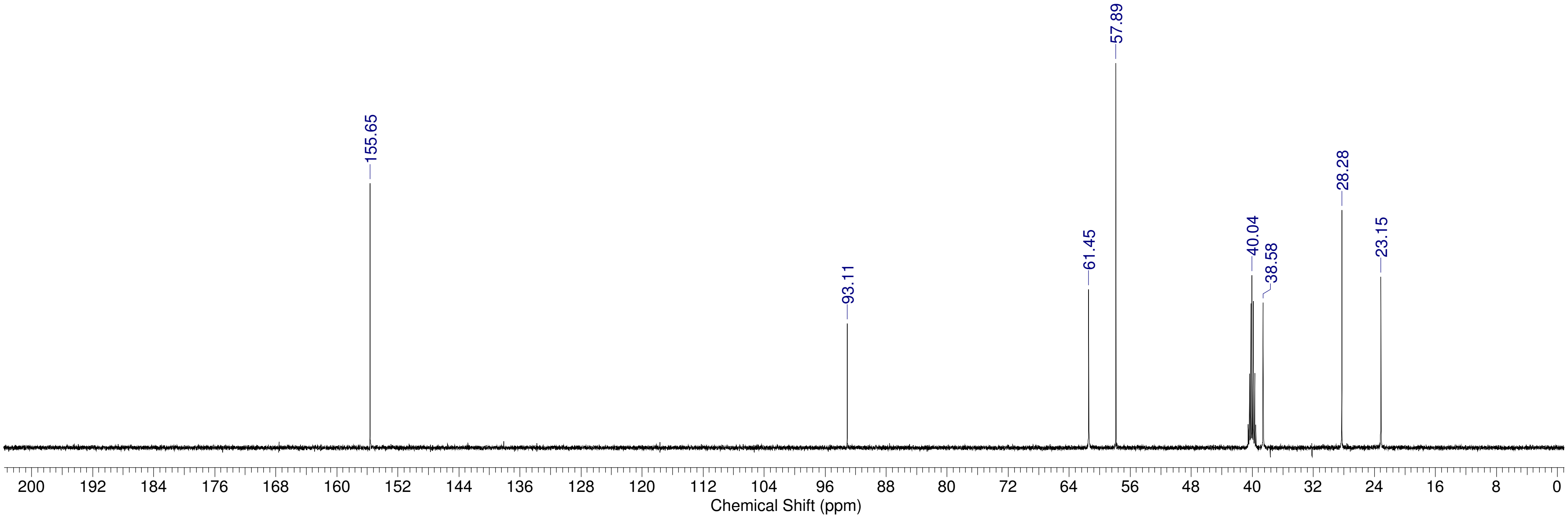

**7** $^{1}$H-NMR ($CDCl_3$, 600 MHz)

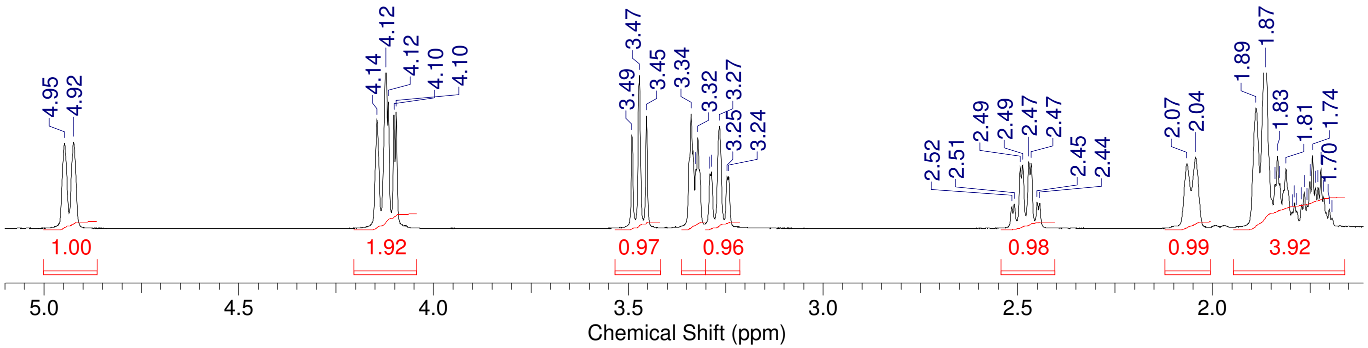


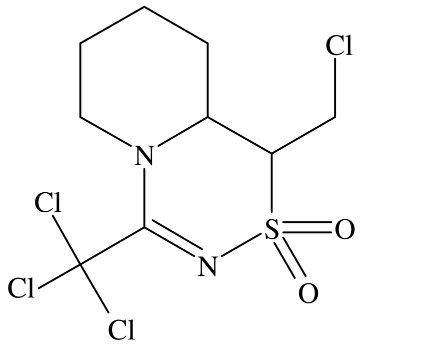


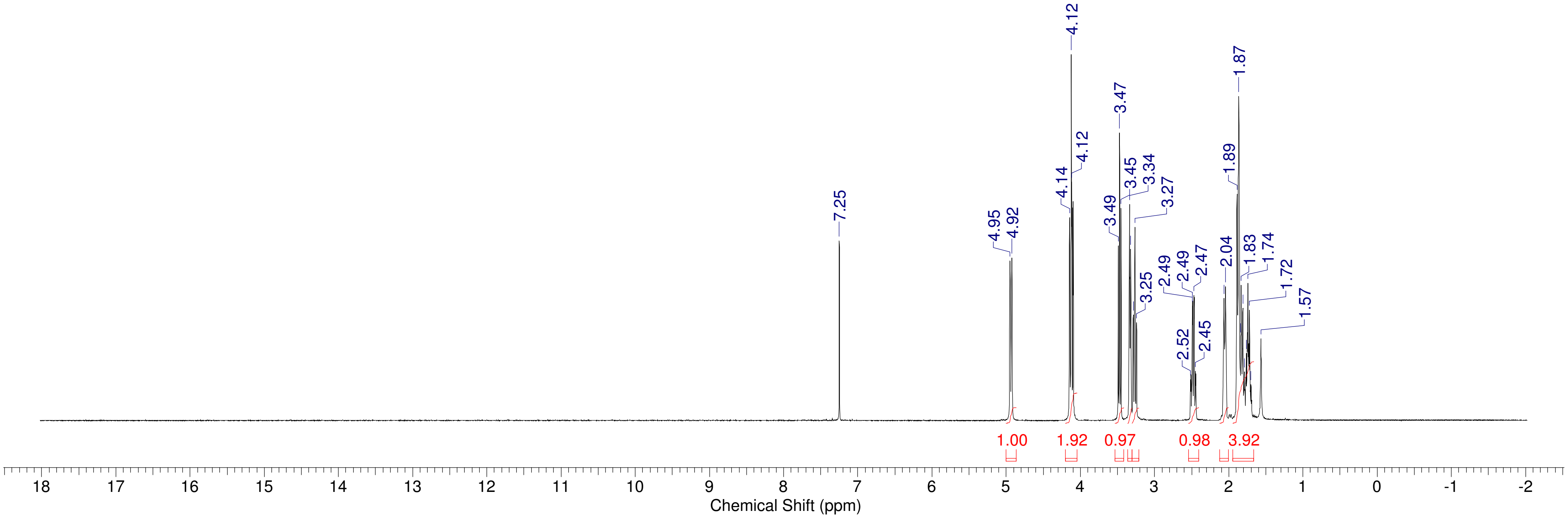

**7** $^{13}$C-NMR ($CDCl_3$, 100 MHz)

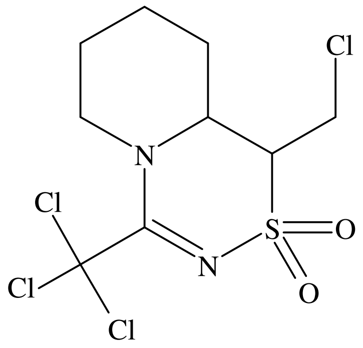


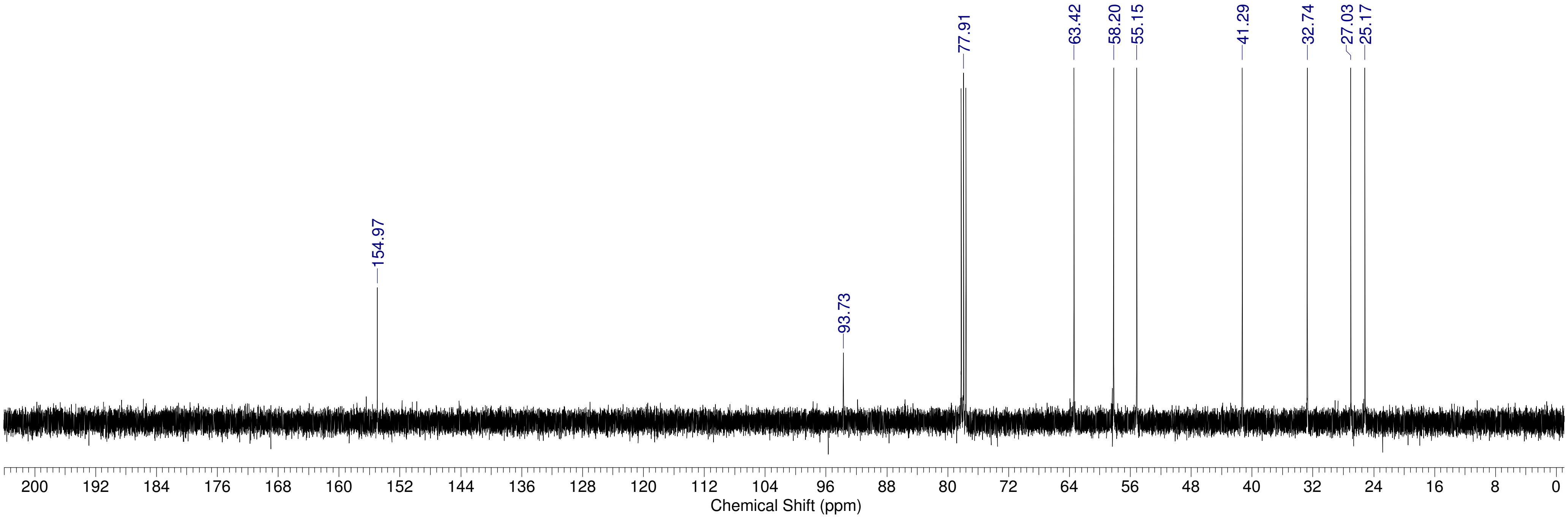

**9** $^1$H-NMR ($CDCl_3$, 600 MHz)

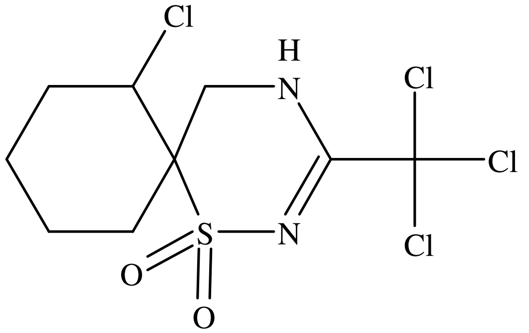

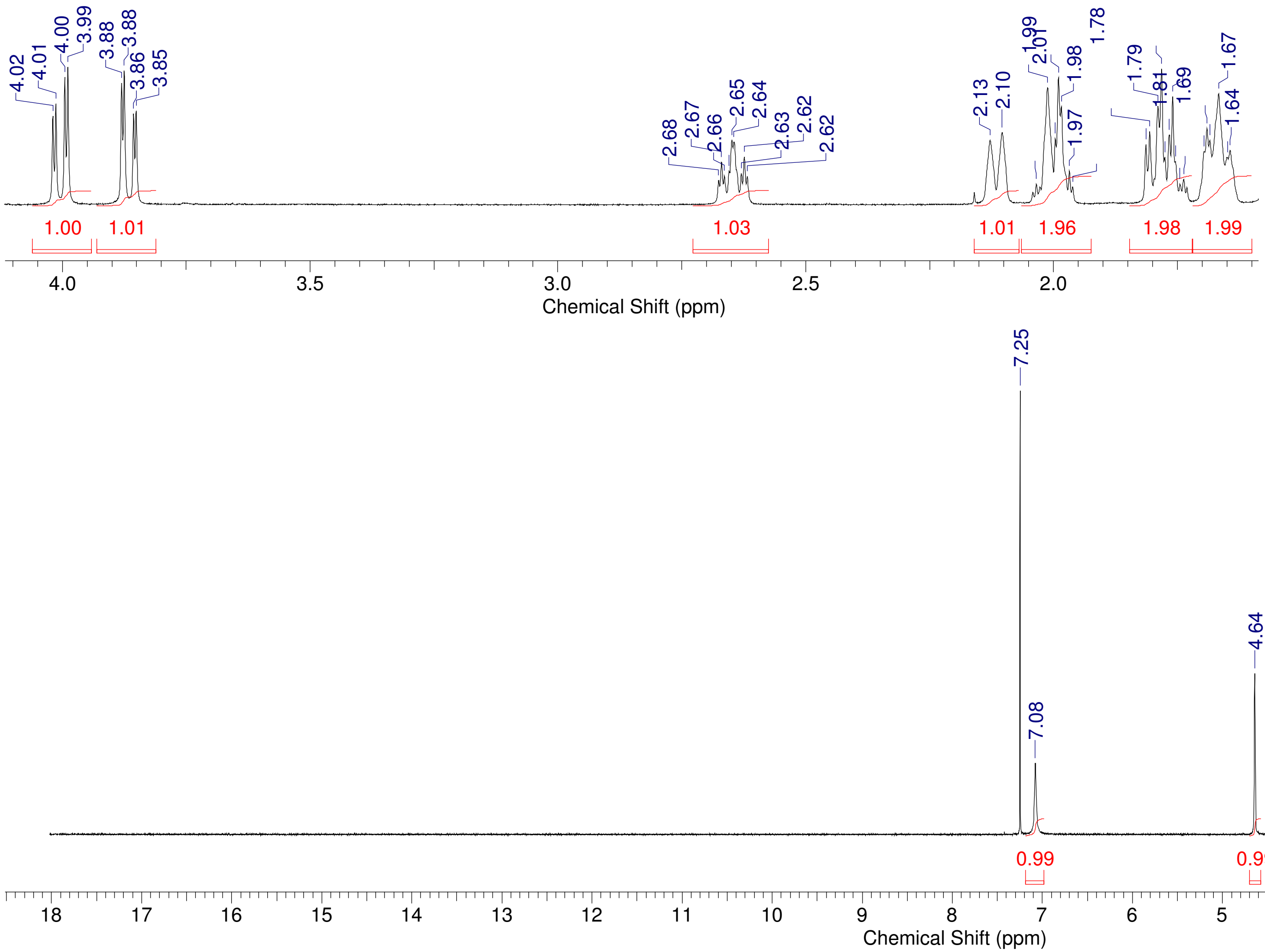

**9** $^{13}C$-NMR ($CDCl_3$, 100 MHz)

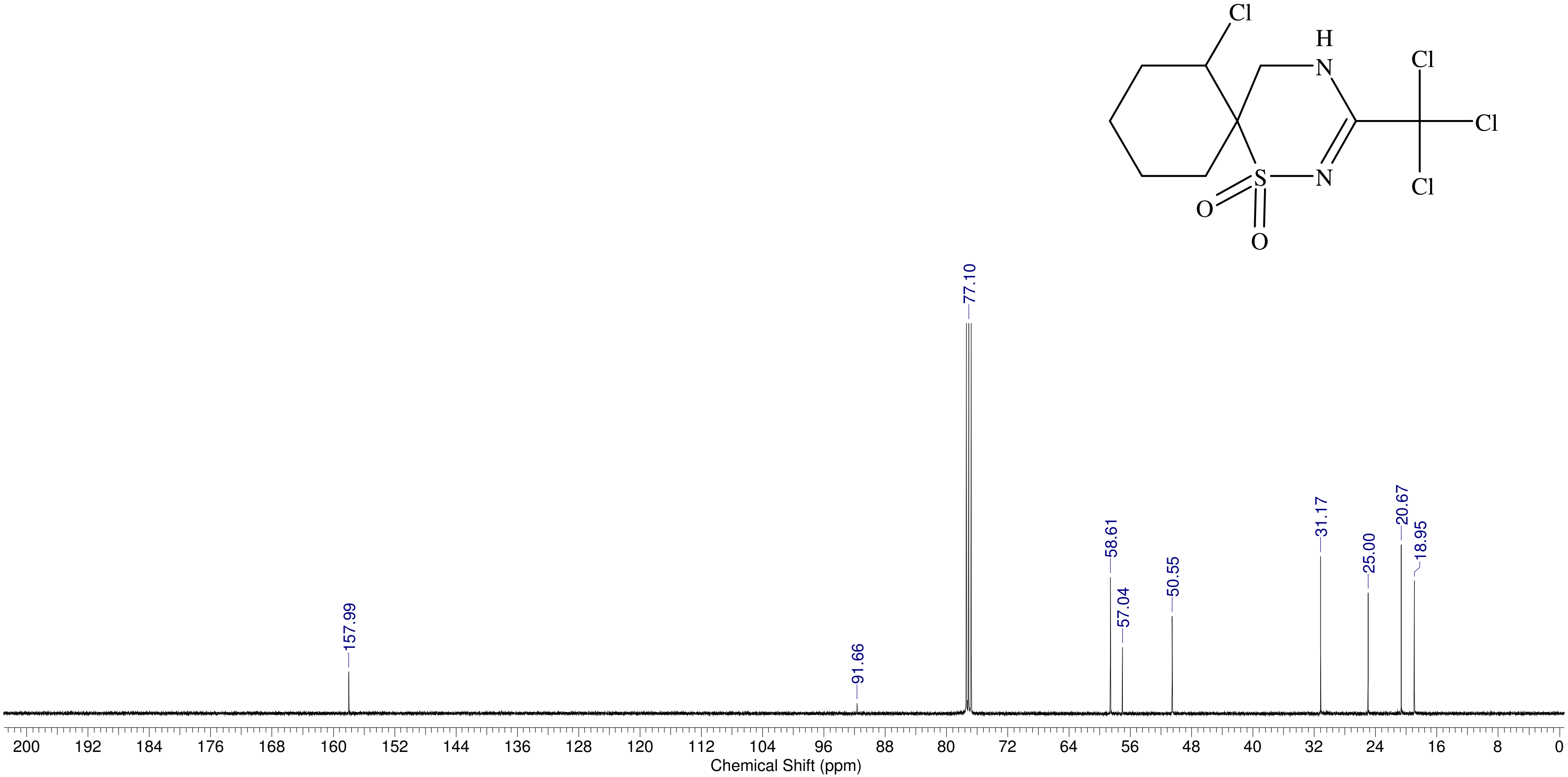

COSY

**9** $^{1}H,^{1}H$-COSY NMR

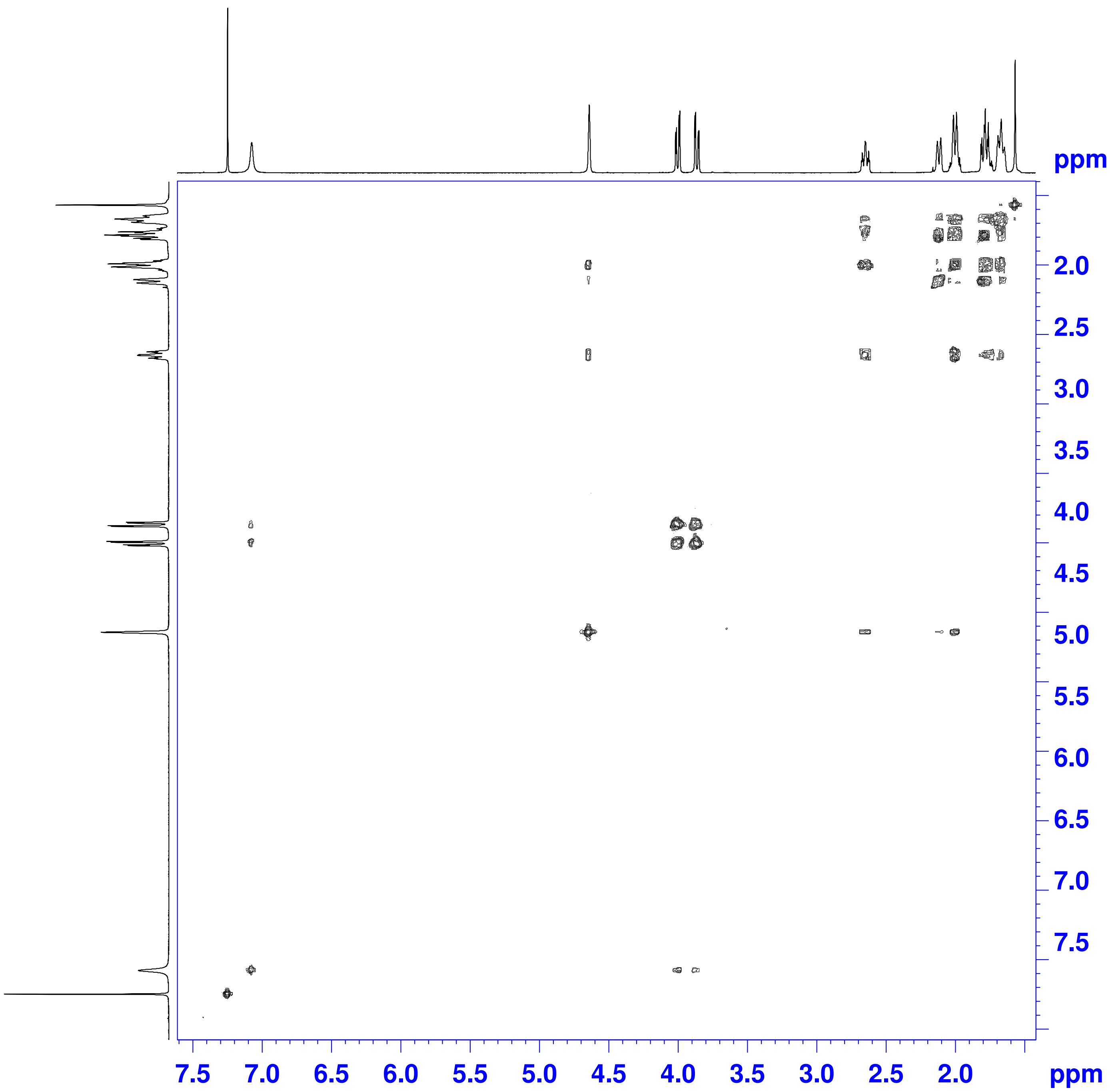


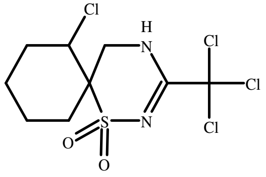

HSQC

**9** HSQC NMR

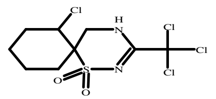

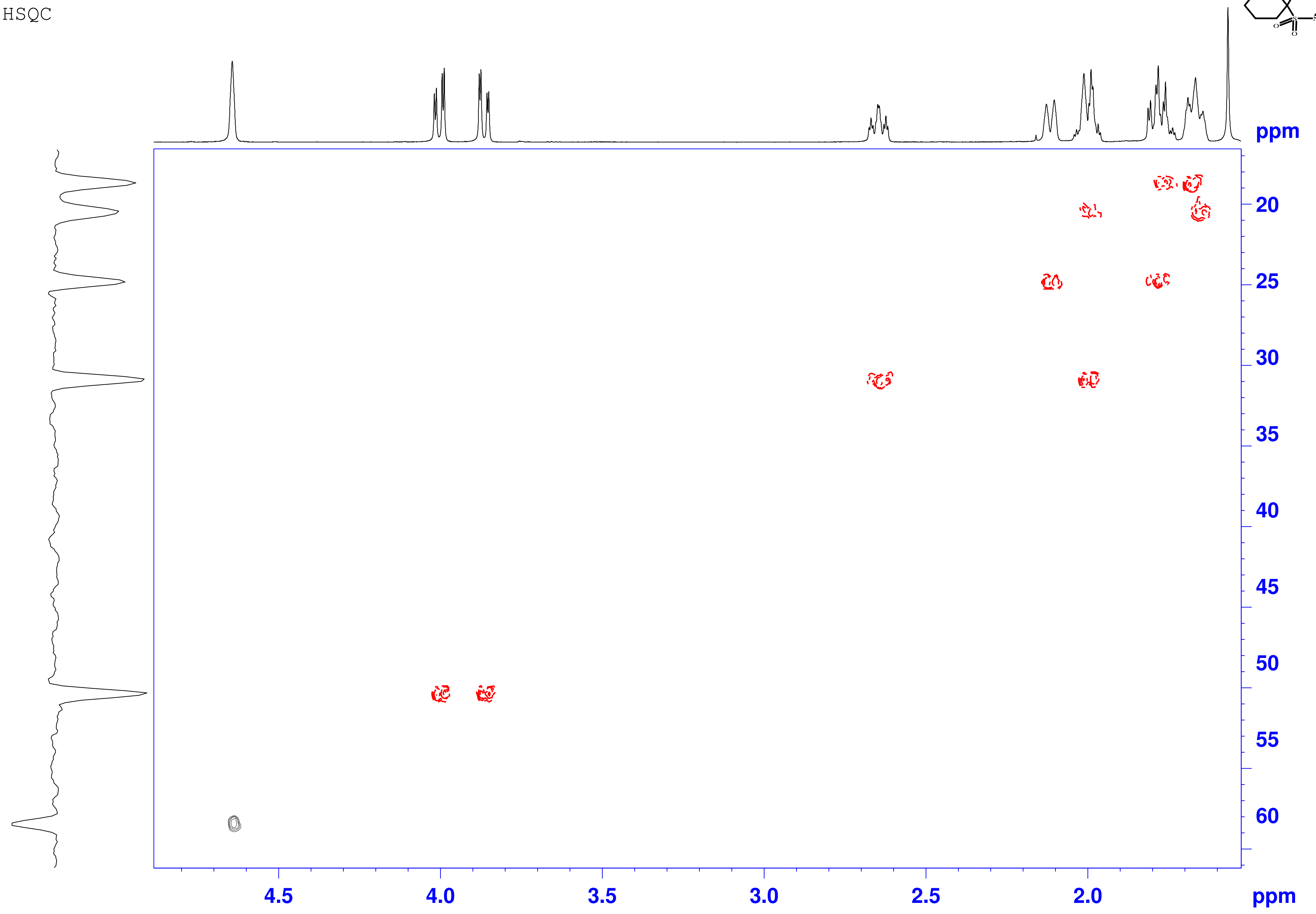

**10** $^1$H-NMR ($CDCl_3$, 600 MHz)

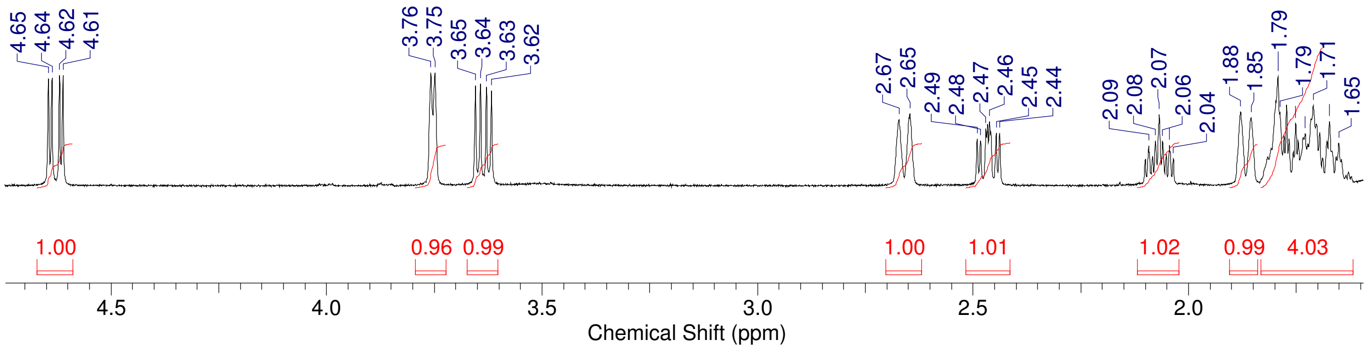

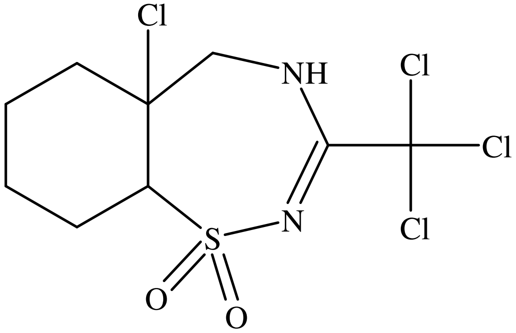


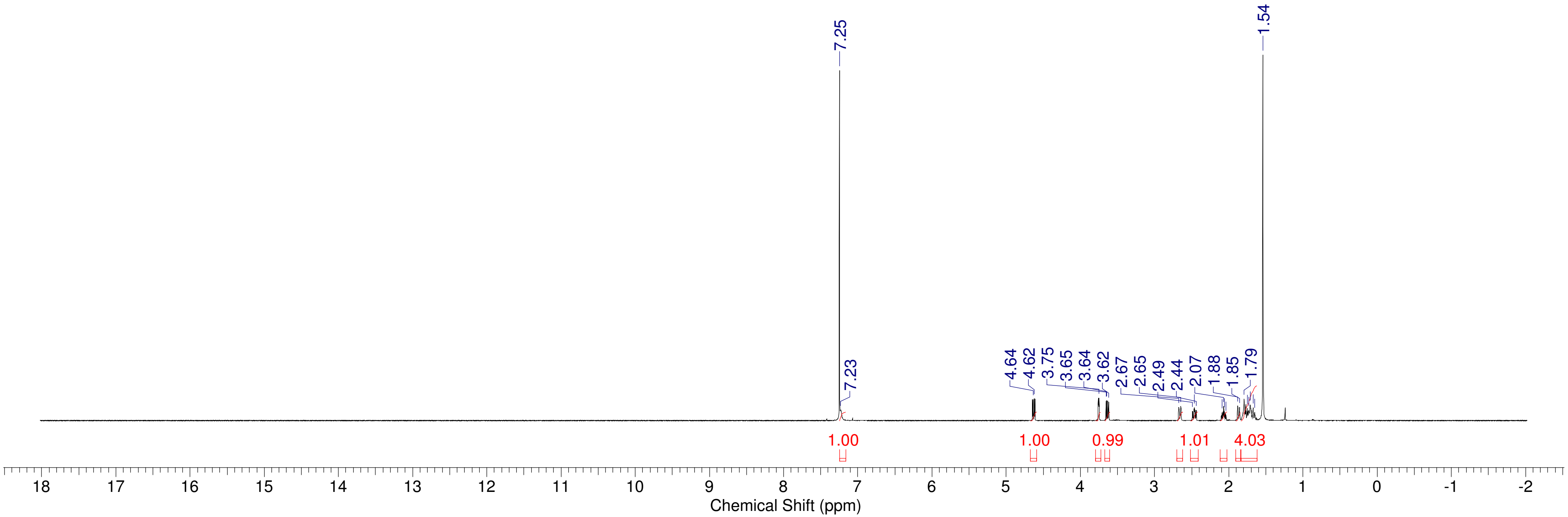

**10** $^{13}$C-NMR (DMSO-d6, 100 MHz)

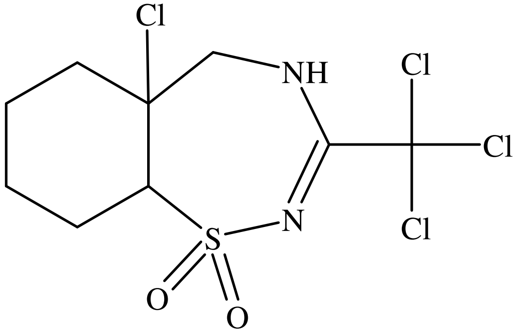


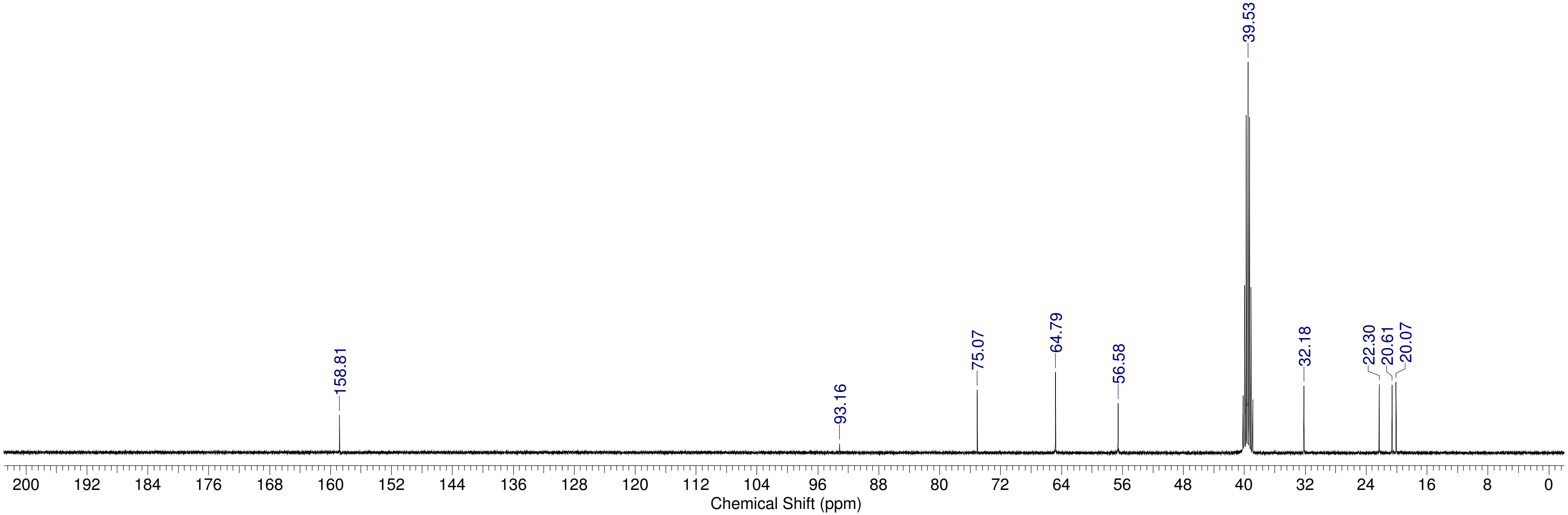

**10** $^{1}H,^{1}H$-COSY NMR

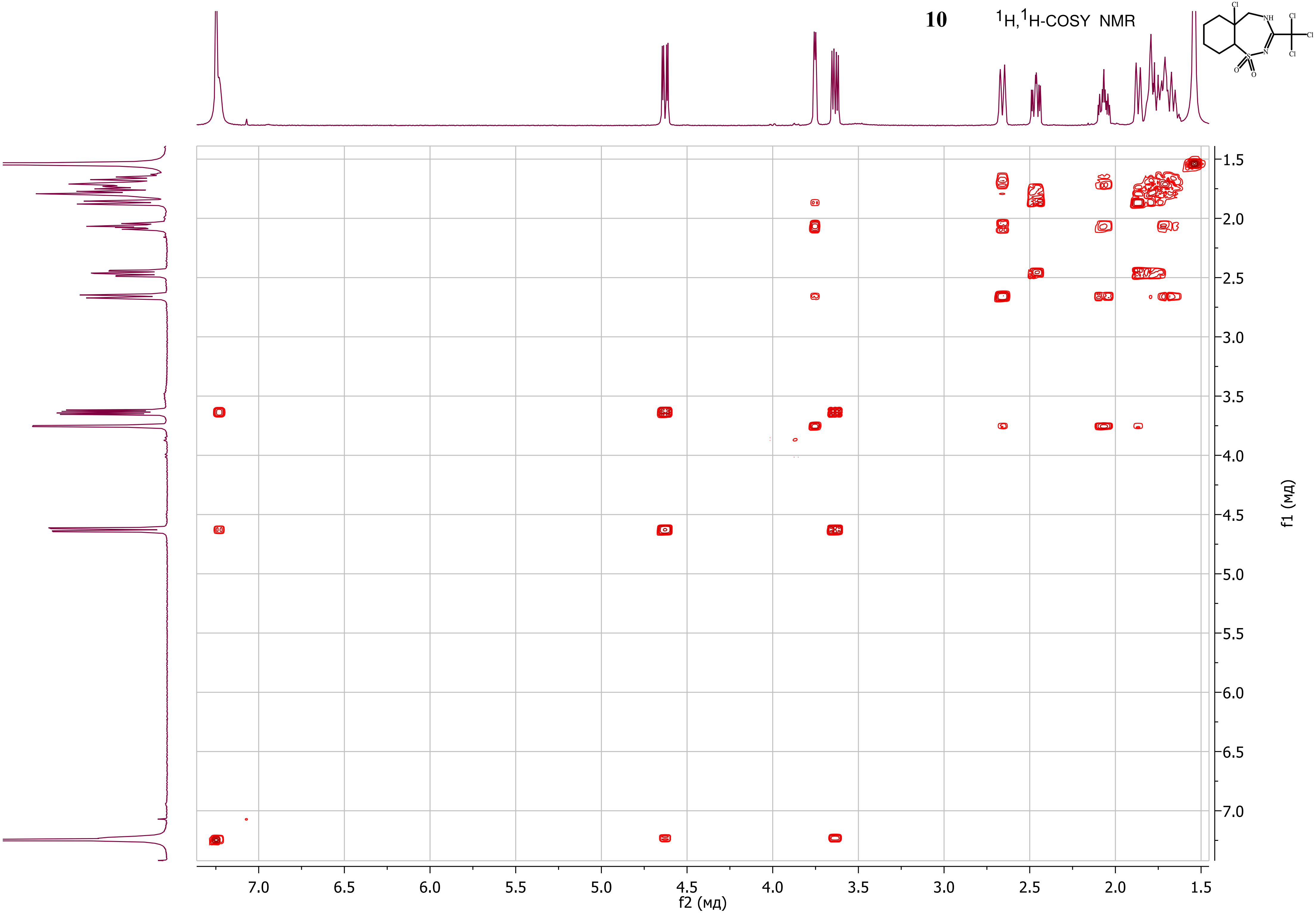

**12**

$^{1}$H-NMR ($CDCl_3$, 600 MHz)

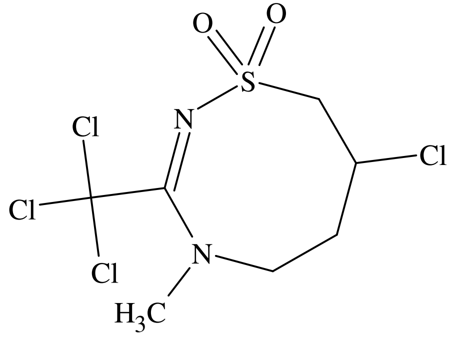


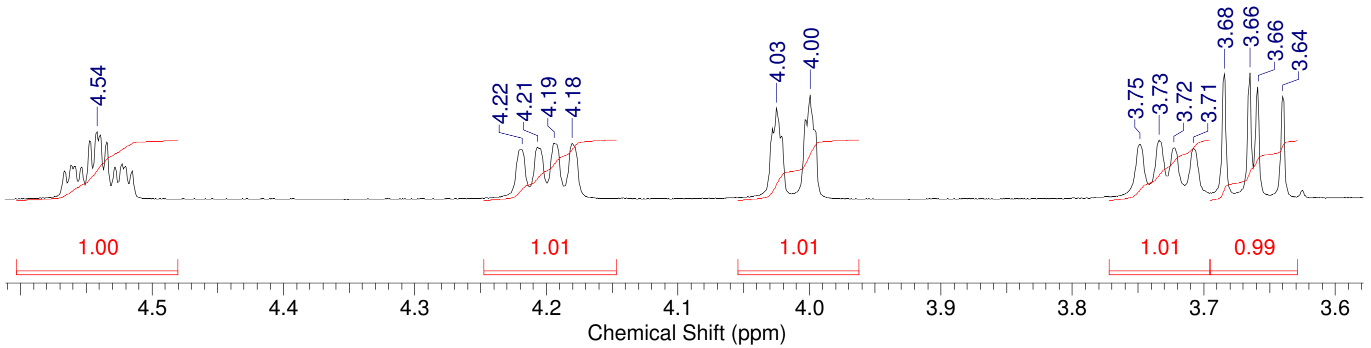

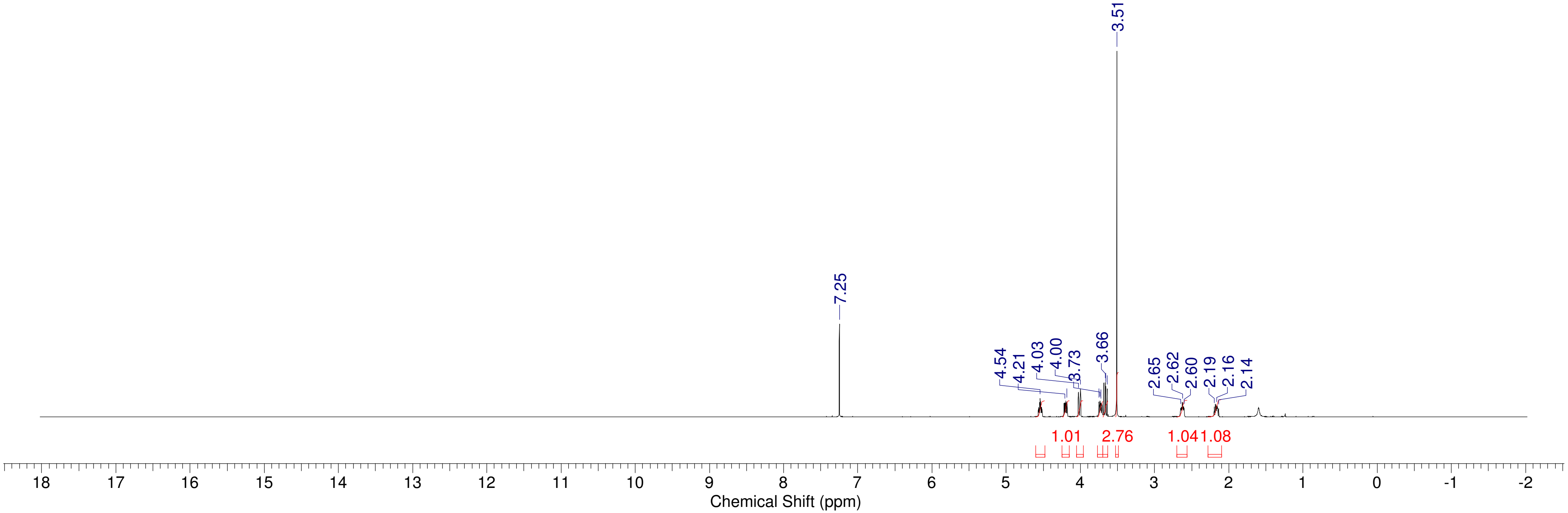

**12** $^{13}$C-NMR (DMSO-d6, 125 MHz)

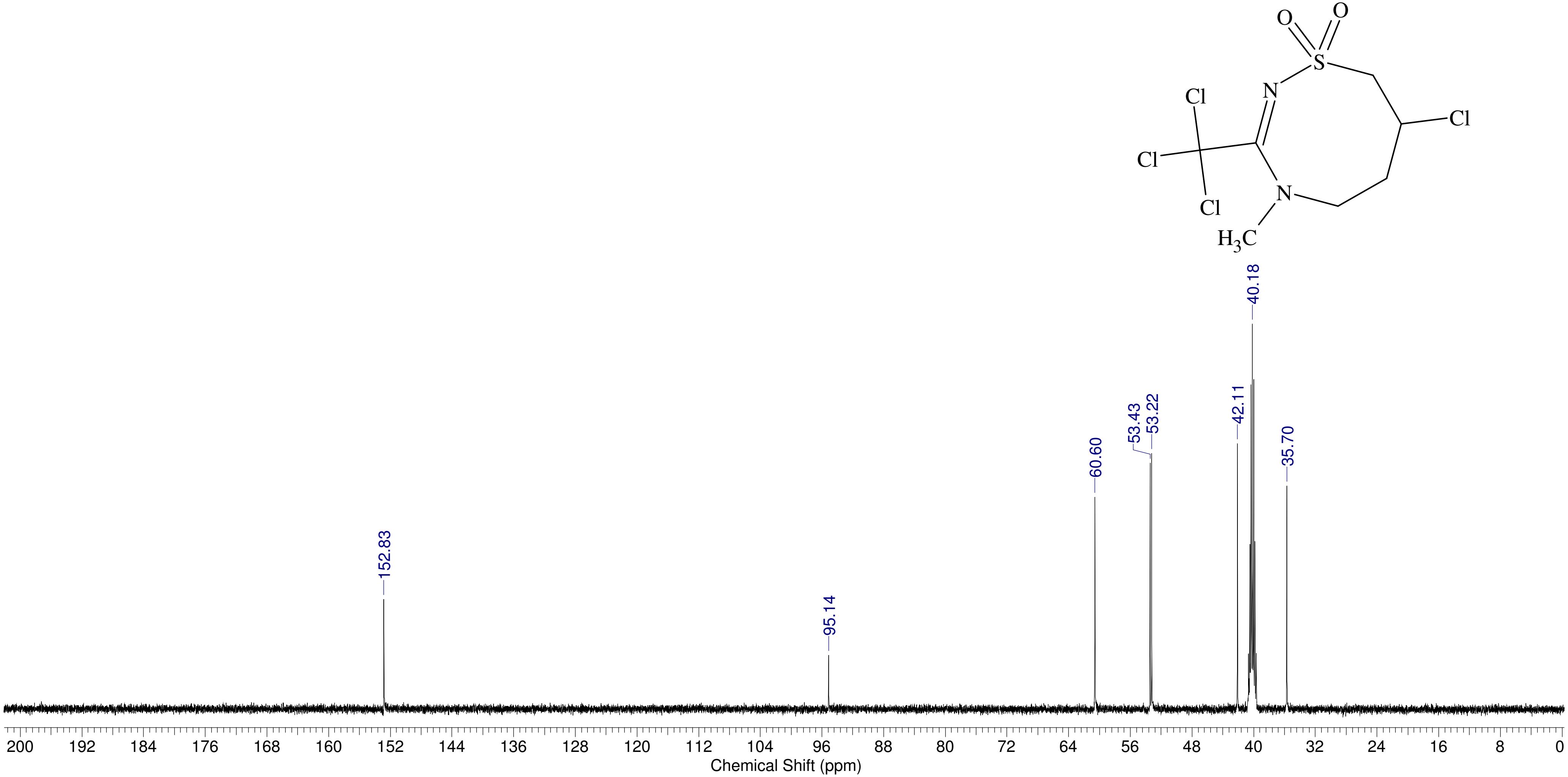

**14** $^{1}$H-NMR ($CDCl_3$, 600 MHz)

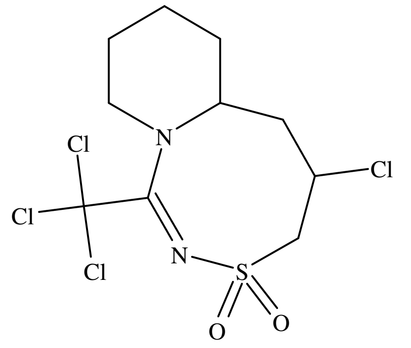


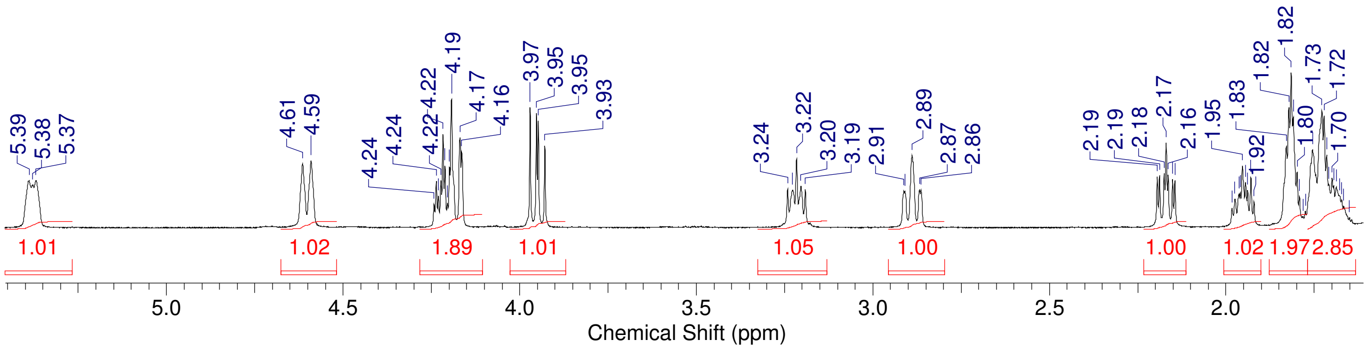


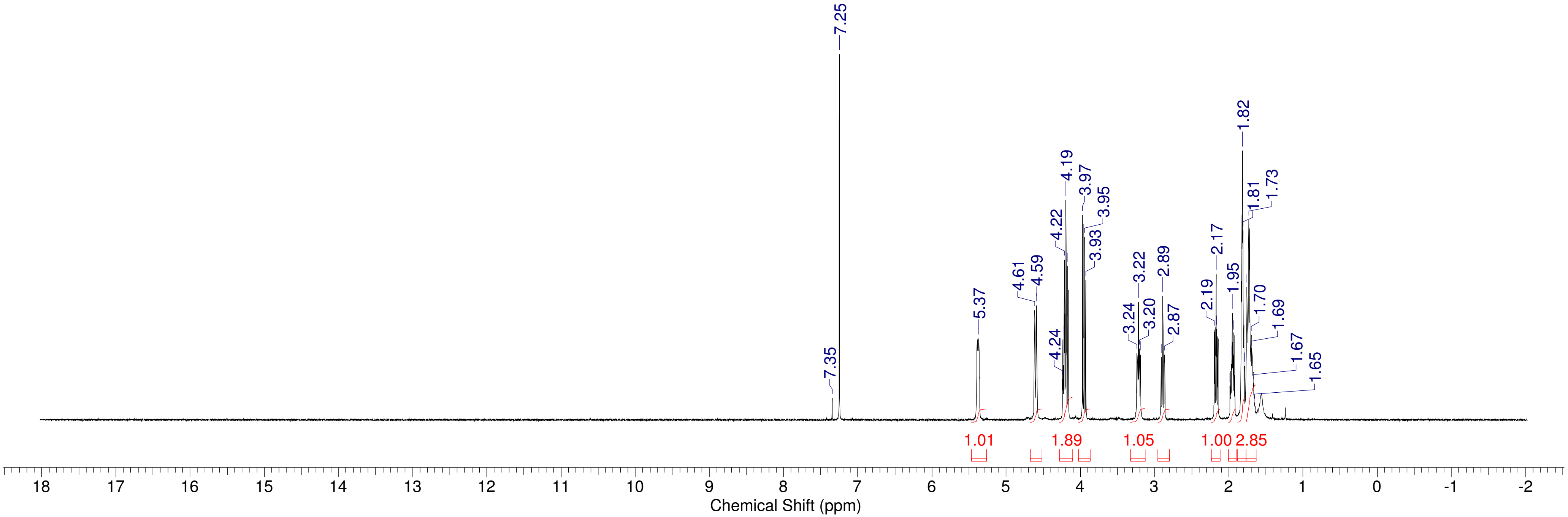

**14** $^{13}$C-NMR ($CDCl_3$, 100 MHz)

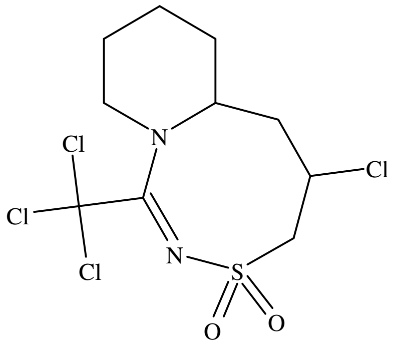


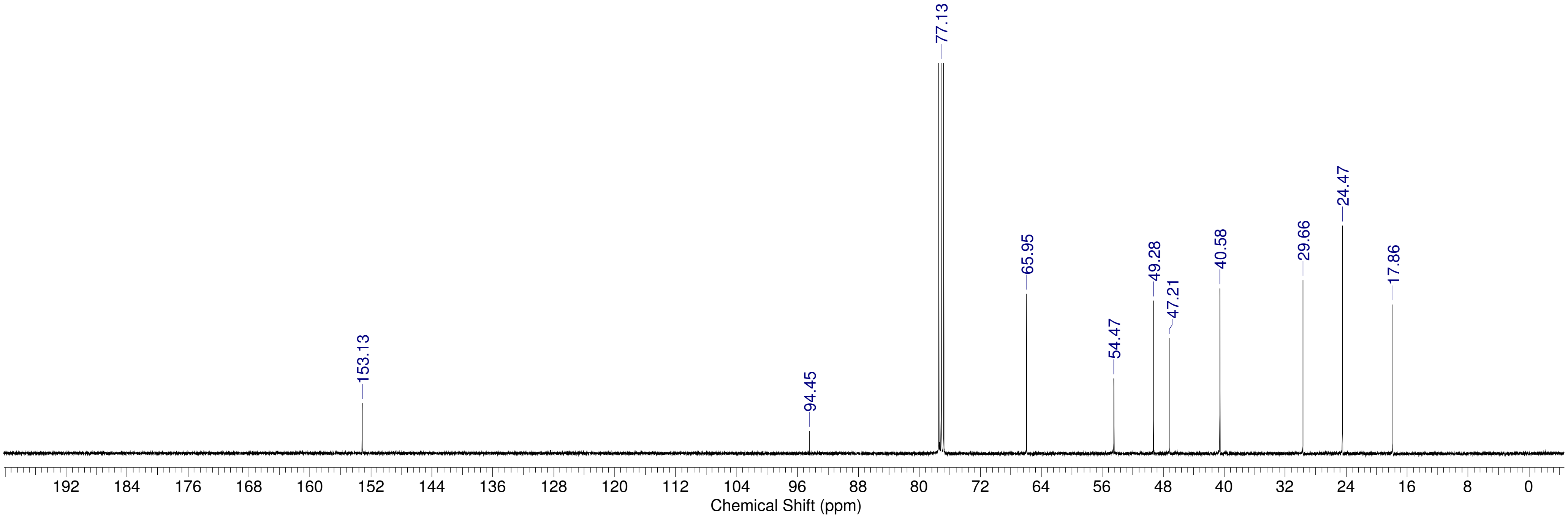

**16a** $^1$H-NMR ($CDCl_3$, 500 MHz)

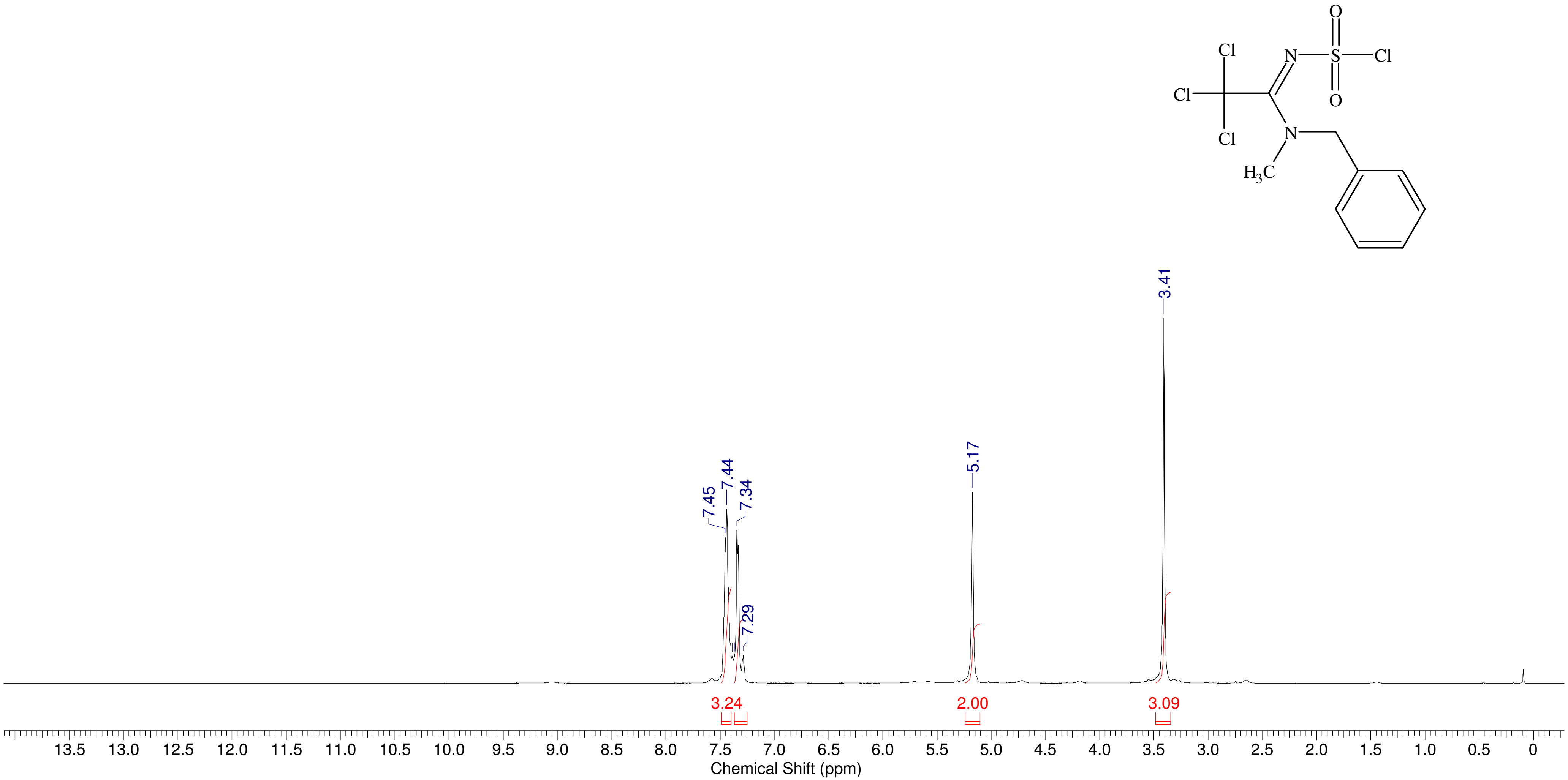

**16a** $^{13}$C-NMR ($CDCl_3$, 125 MHz)

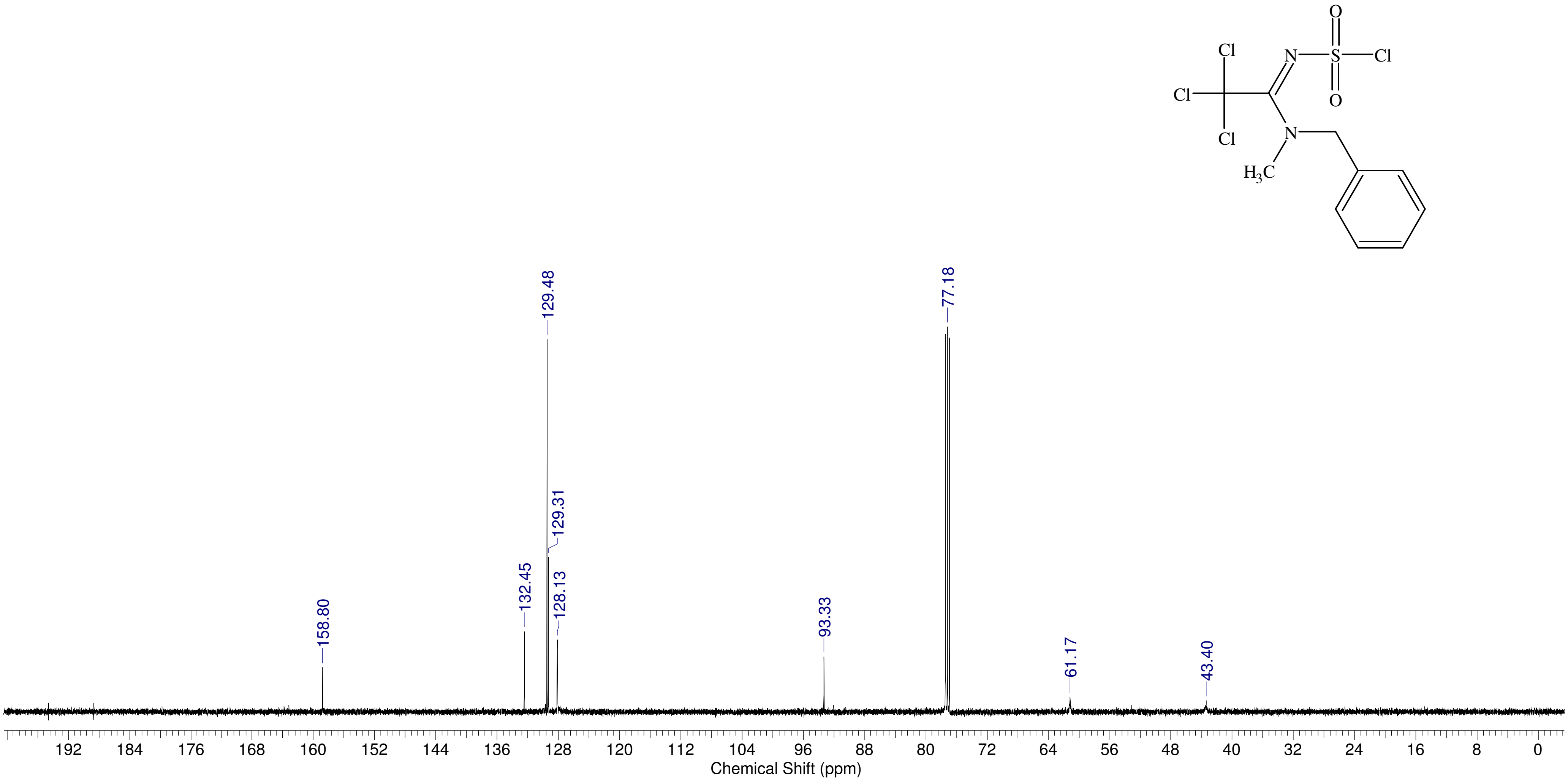

**16b** $^{1}$H-NMR ($CDCl_3$, 600 MHz)

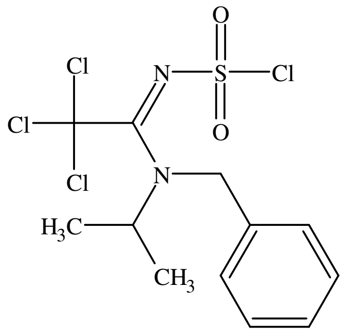


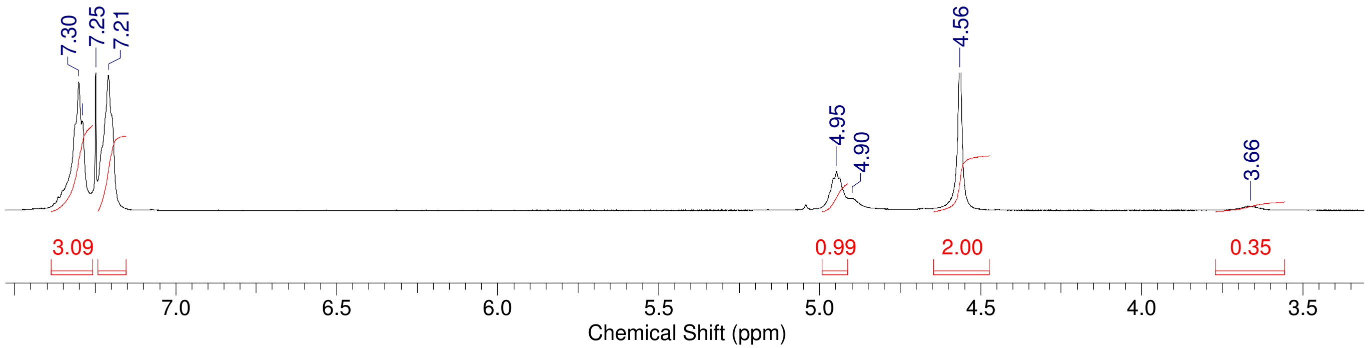


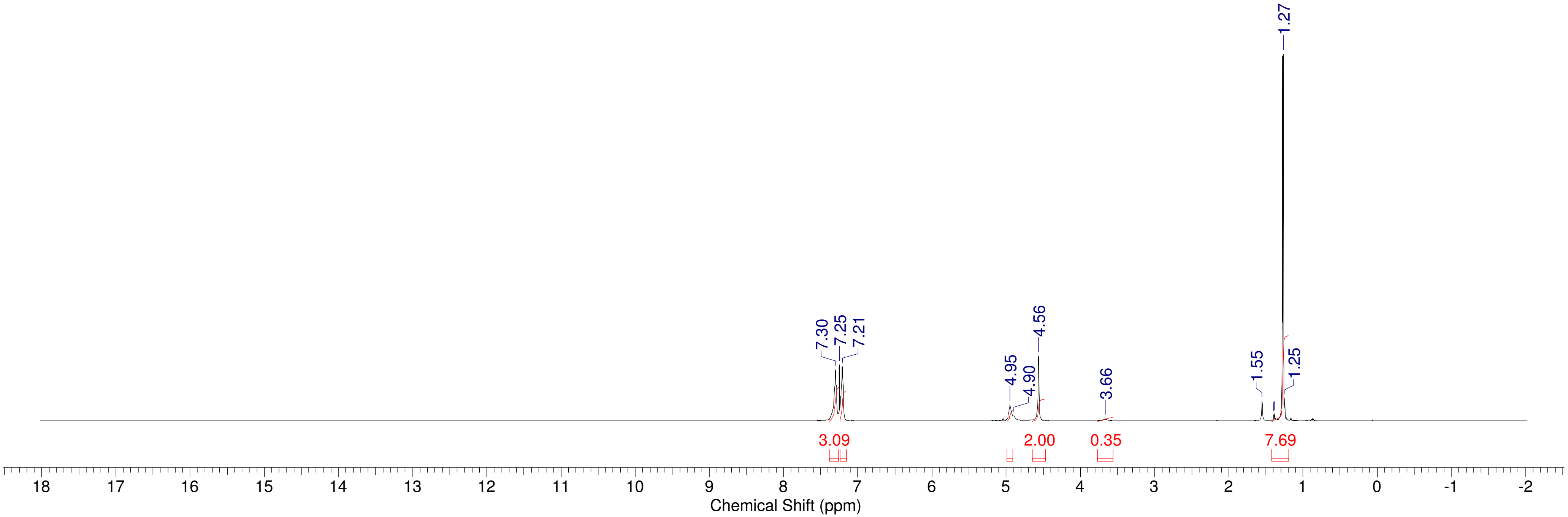

**16b** $^{13}$C-NMR (CDCl$_3$+DMSO-d6, 125 MHz)

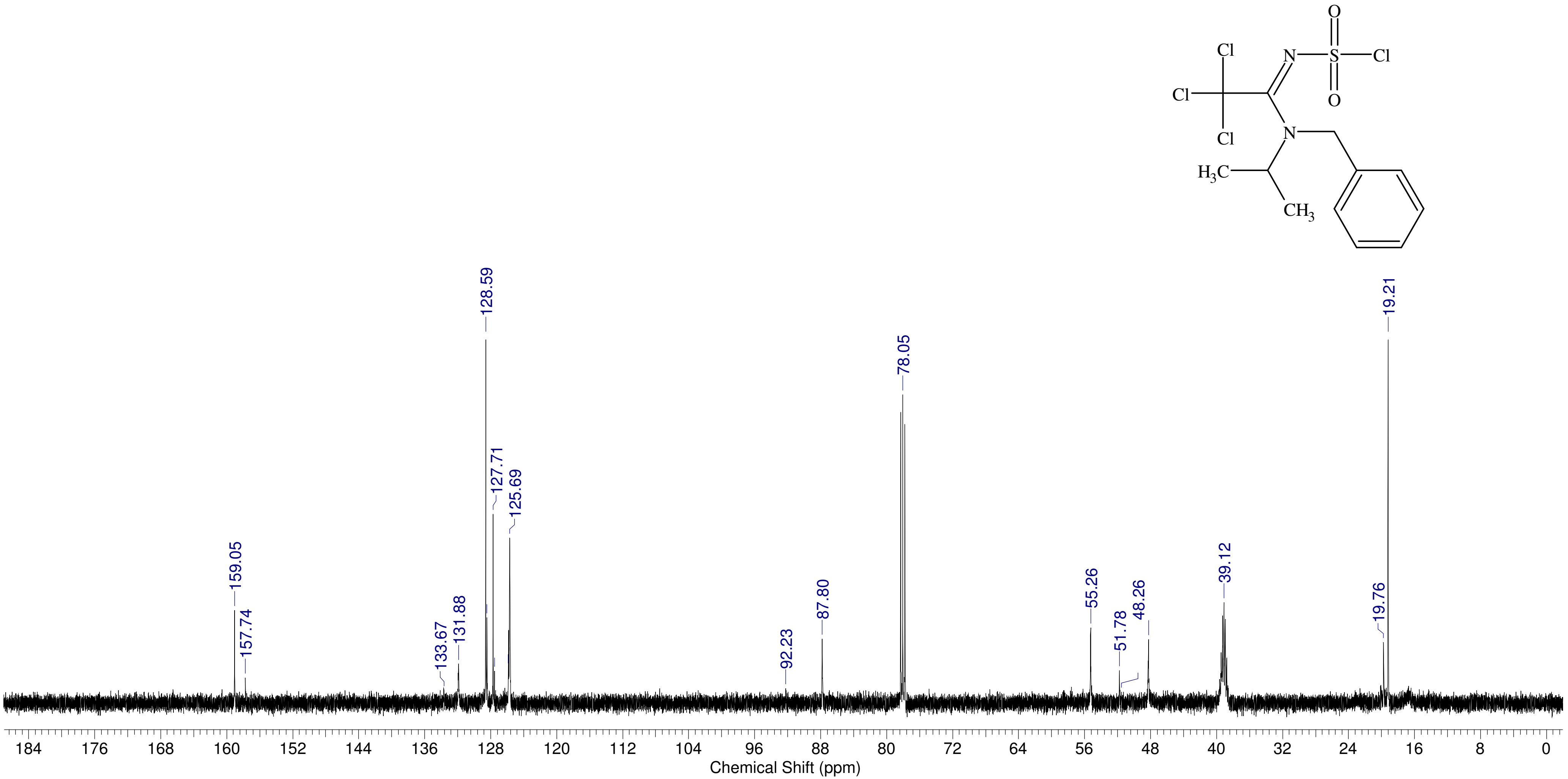

**17a** $^1$H-NMR ($CDCl_3$, 500 MHz)

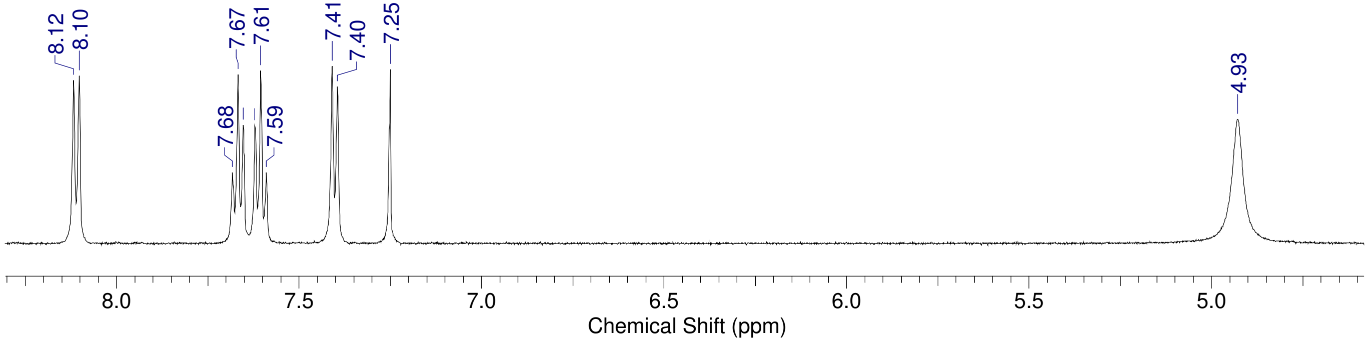


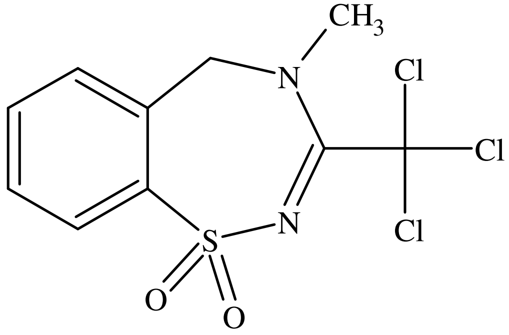


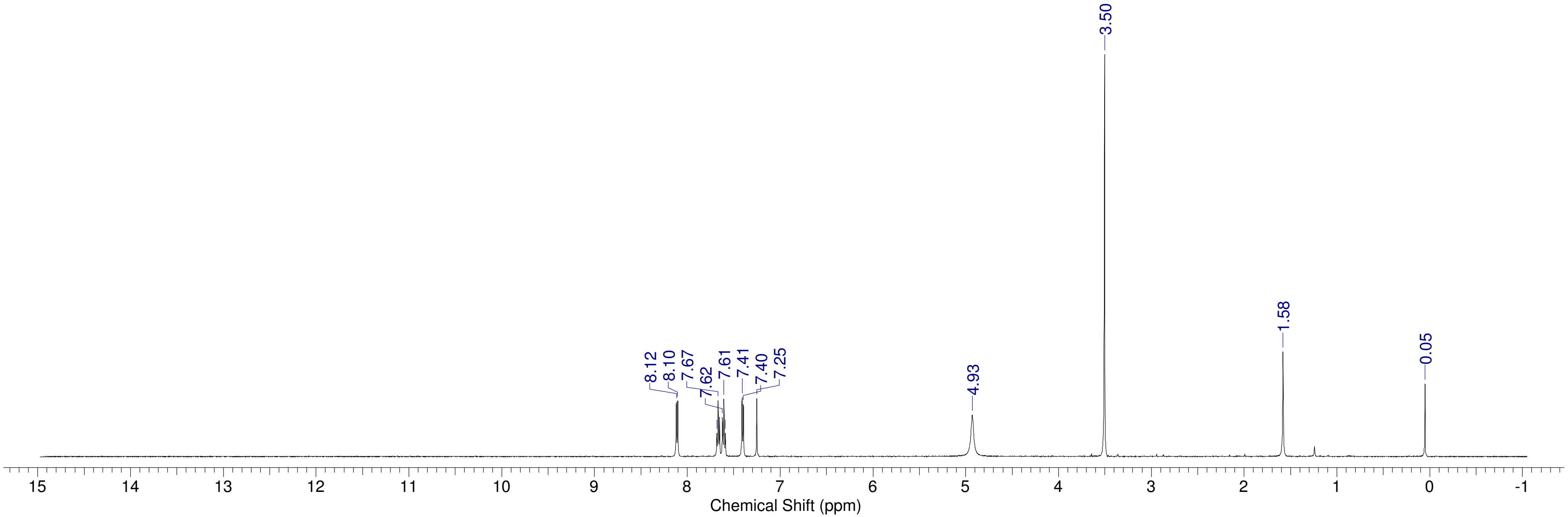

**17a** $^{13}$C-NMR (DMSO-d6, 150 MHz)

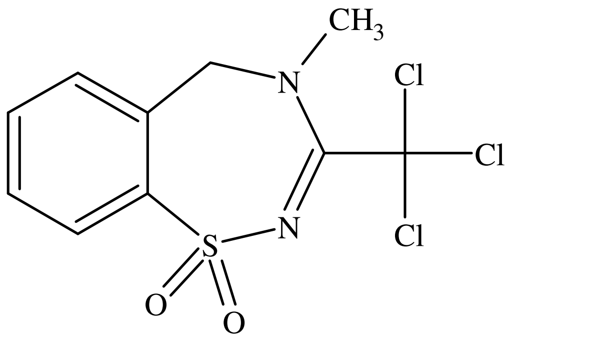

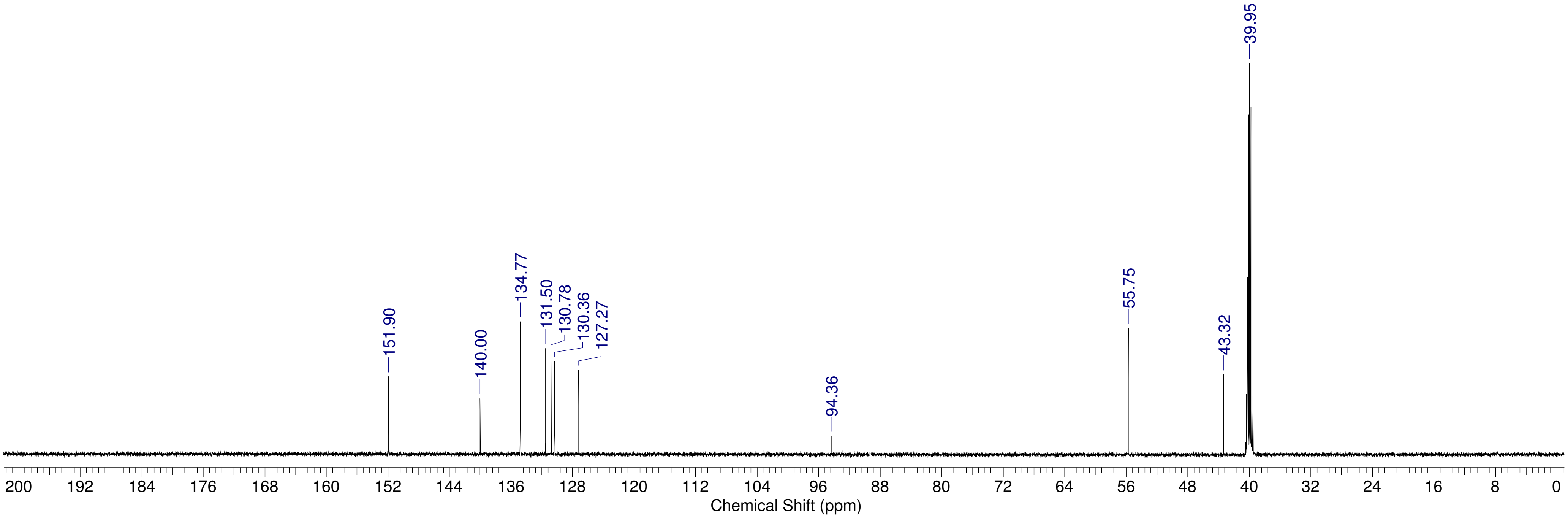

**17b** $^1$H-NMR ($CDCl_3$, 600 MHz)

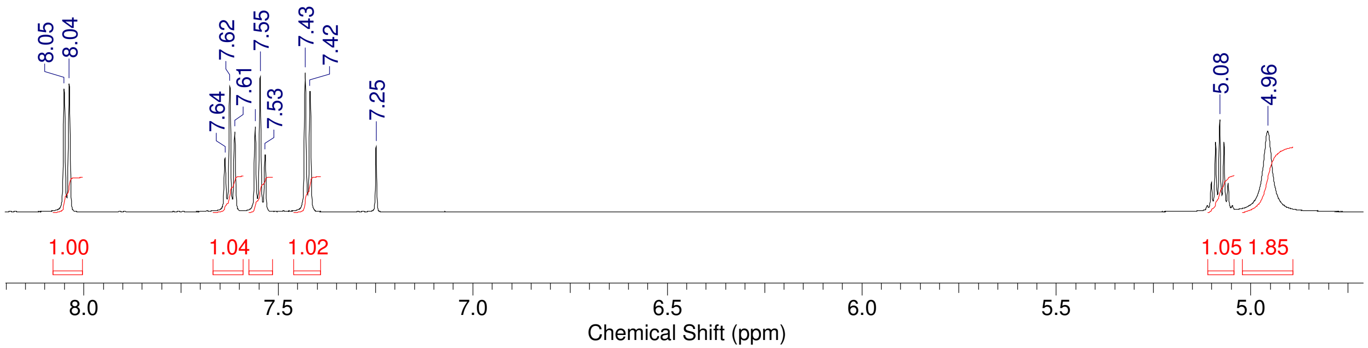

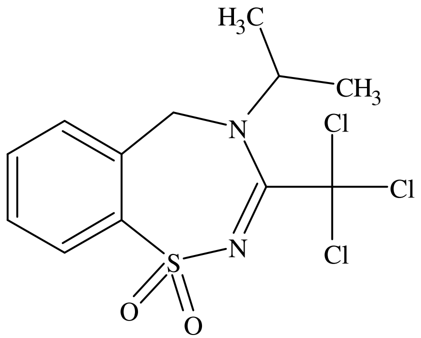


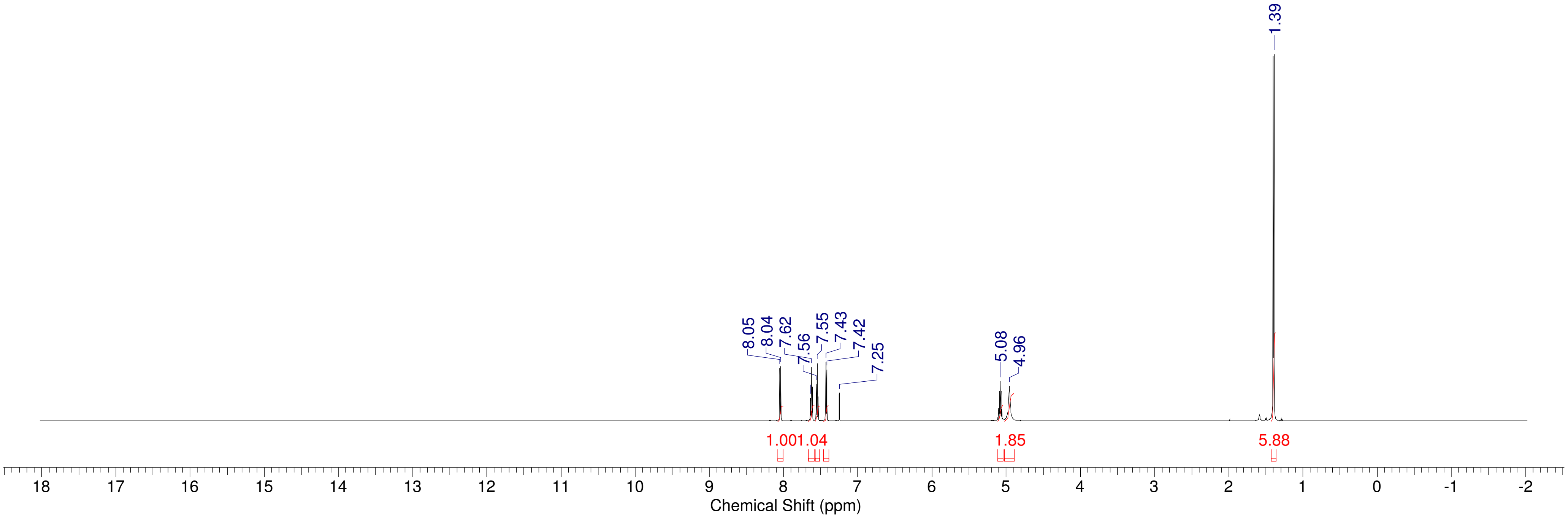

**17b** $^{13}$C-NMR (DMSO-d6, 125 MHz)

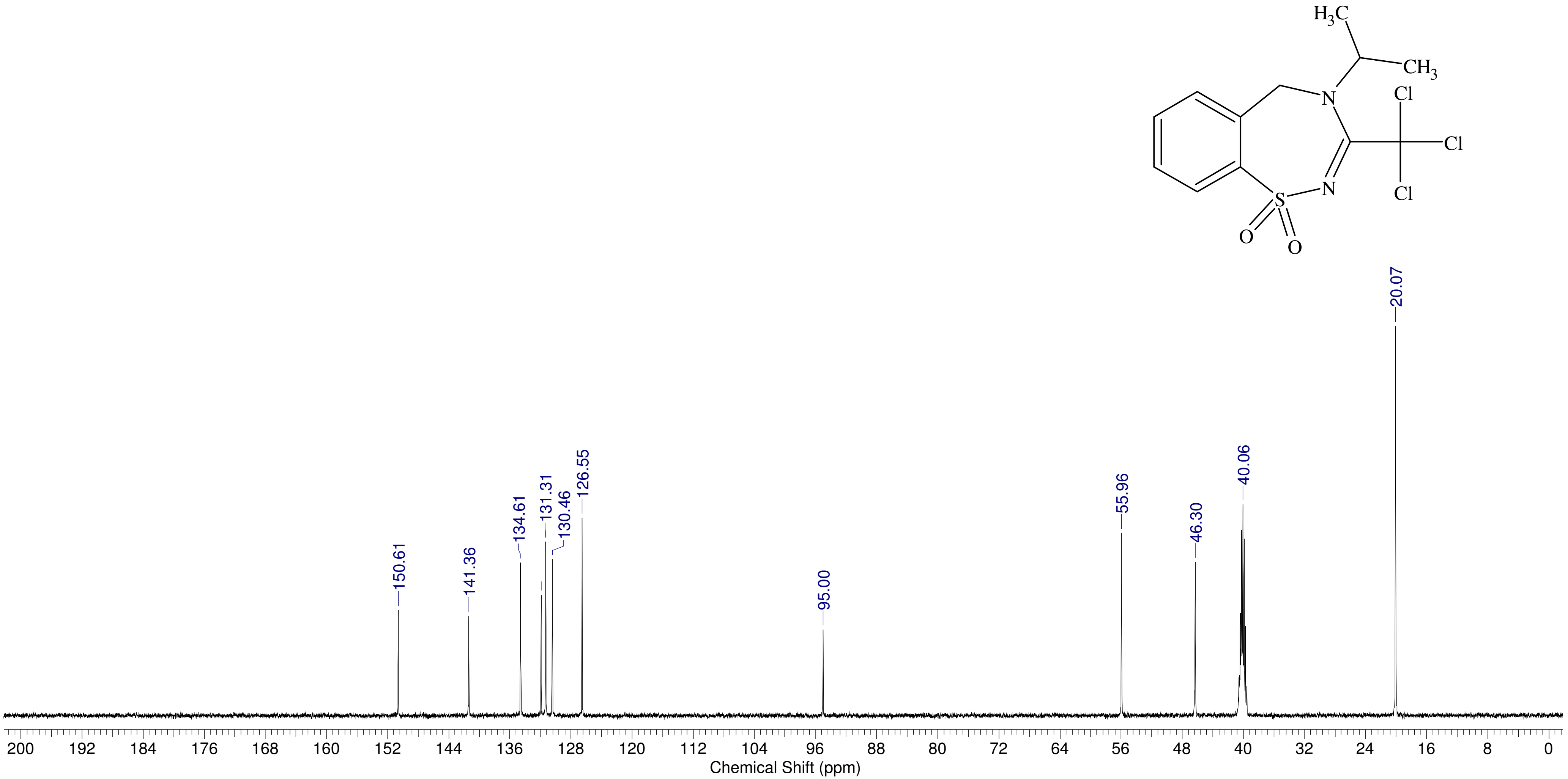

**18** $^{1}$H-NMR ($CDCl_3$+DMSO-d6, 500 MHz)

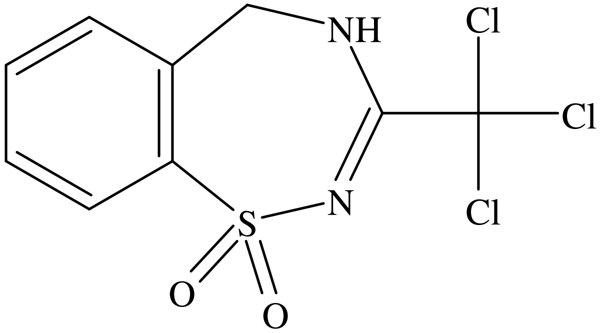


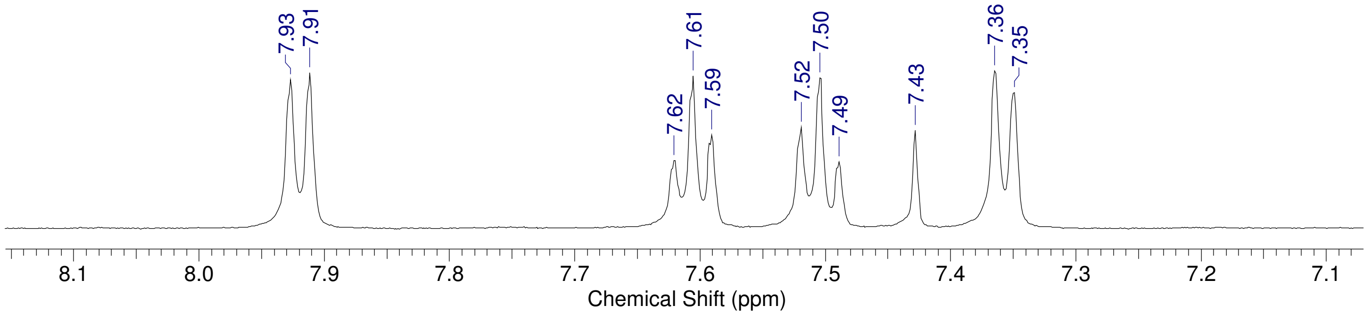


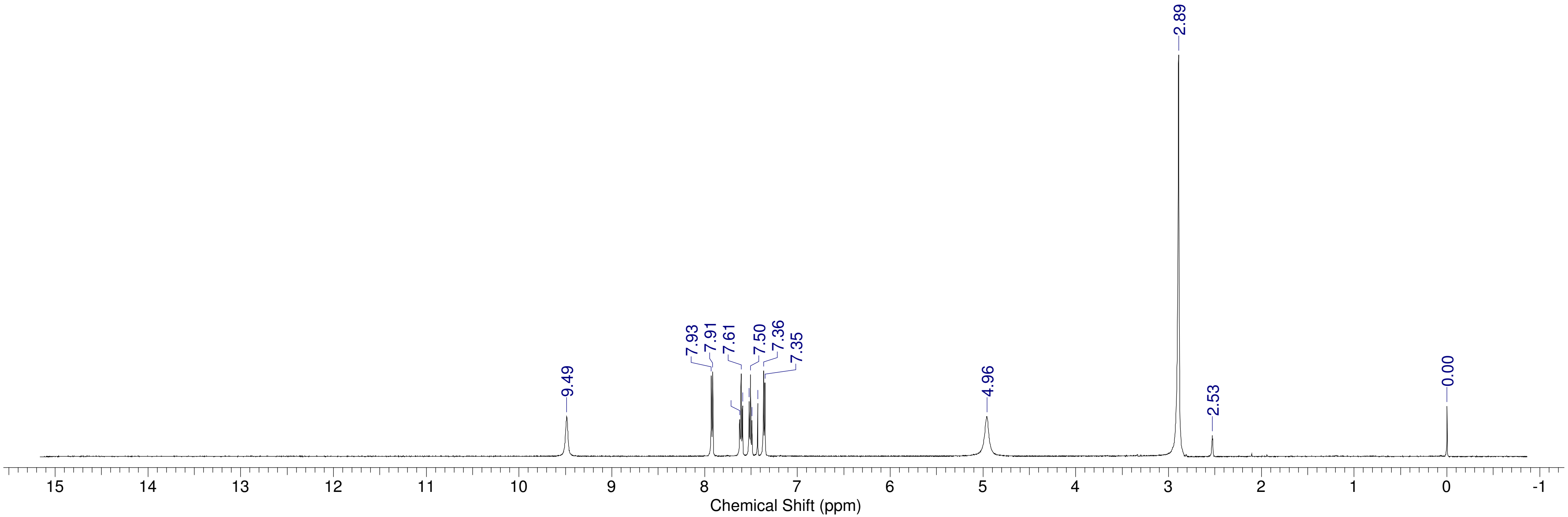

**18** $^{13}C$-NMR (DMSO-d6, 125 MHz)

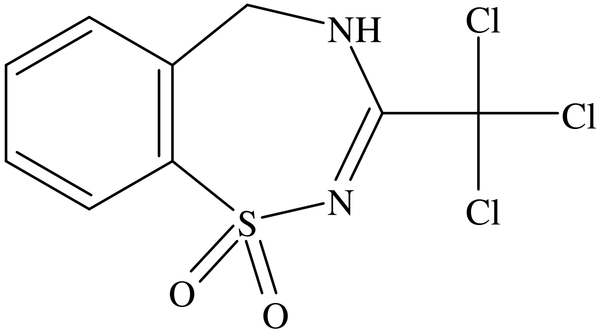


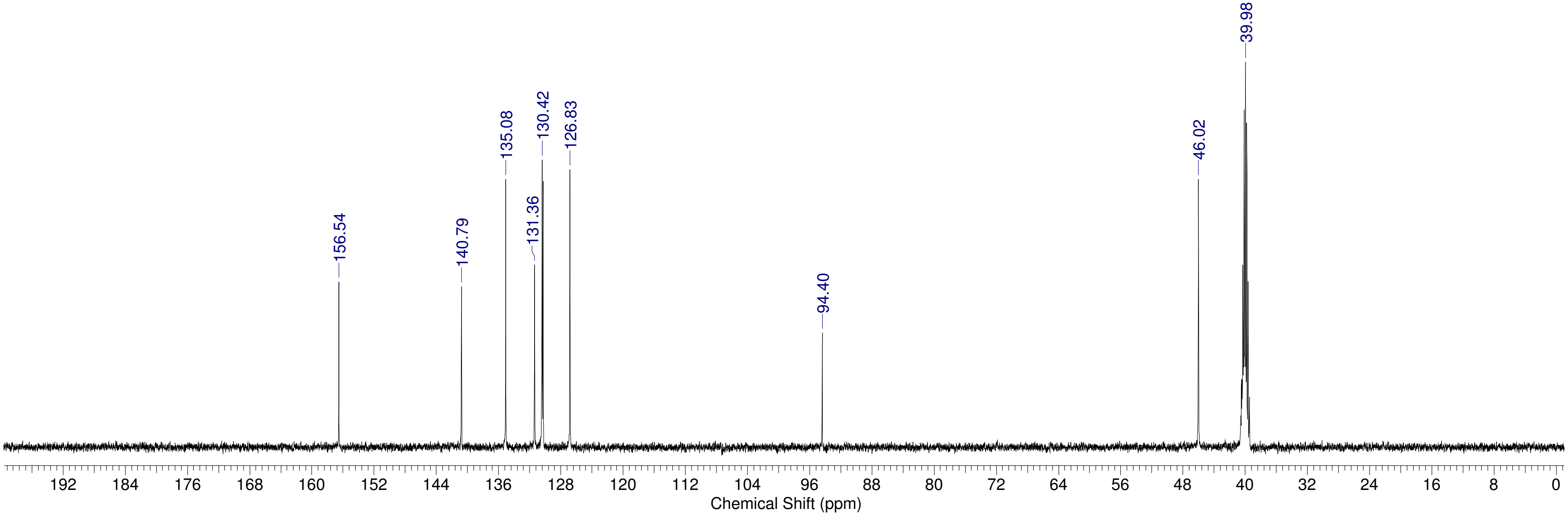

**19** $^{1}$H-NMR ($CDCl_3$, 600 MHz)

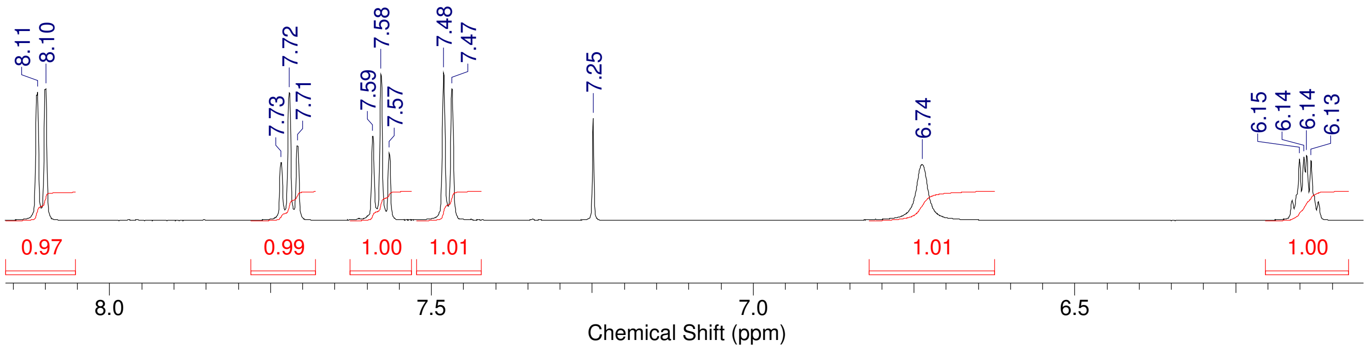


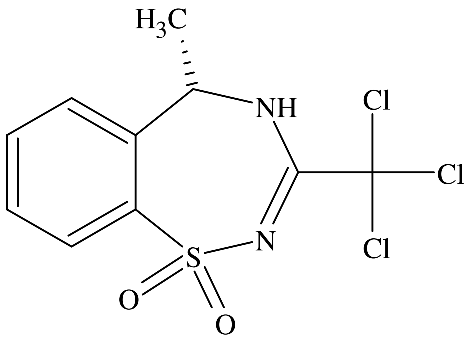


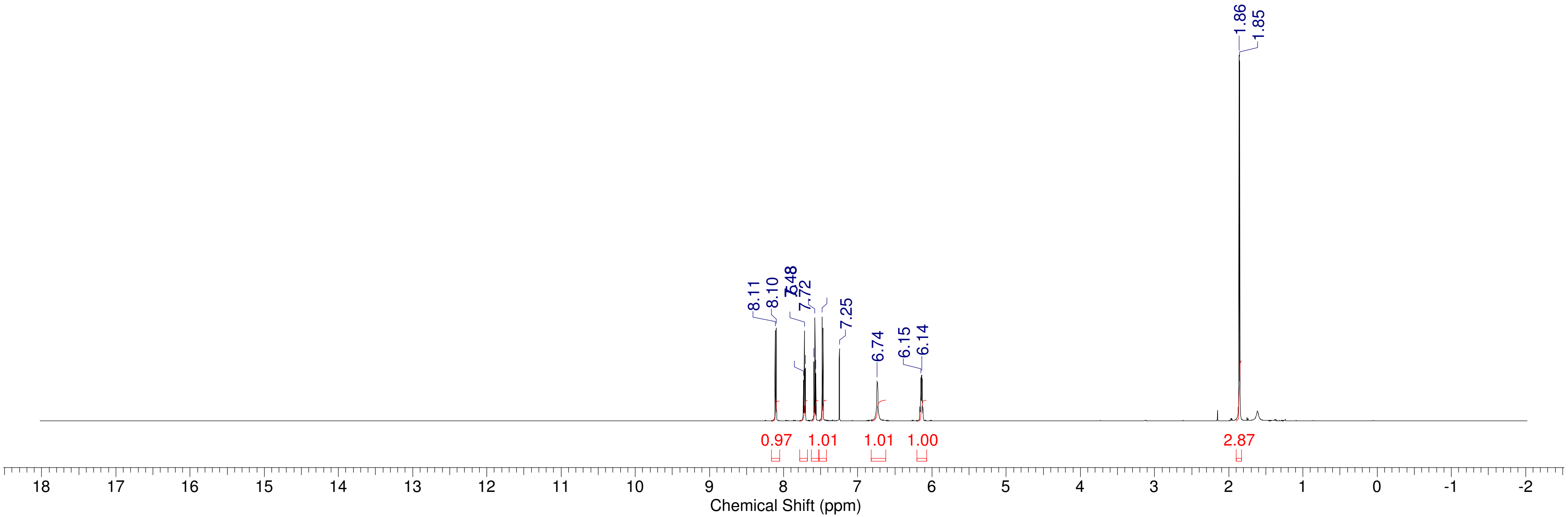

**19** $^{13}$C-NMR (CDCl$_3$, 150 MHz)

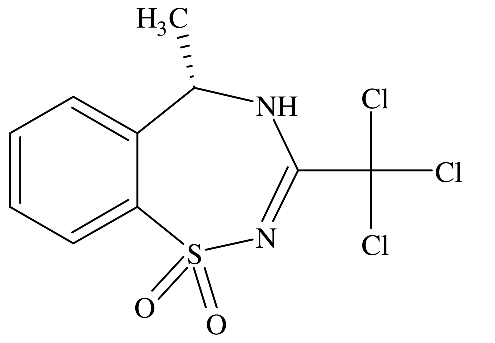


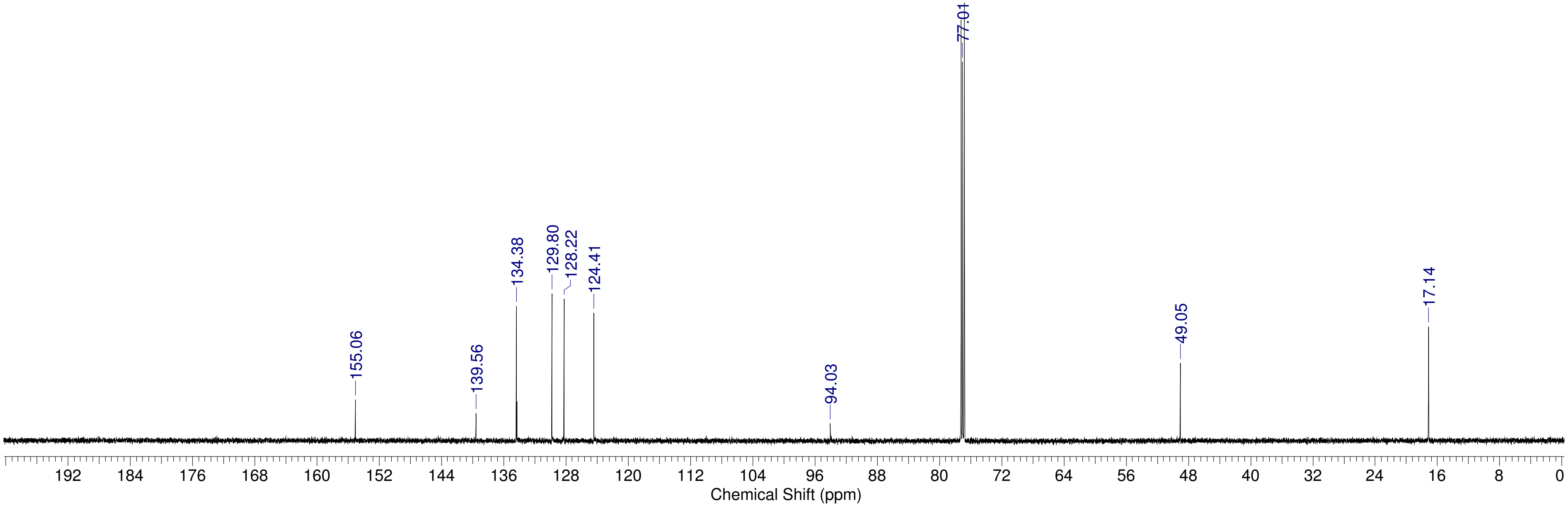

**20** $^{1}$H-NMR ($CDCl_3$, 400 MHz)

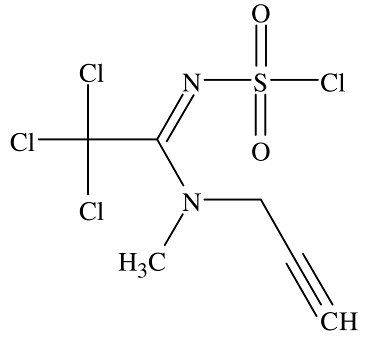


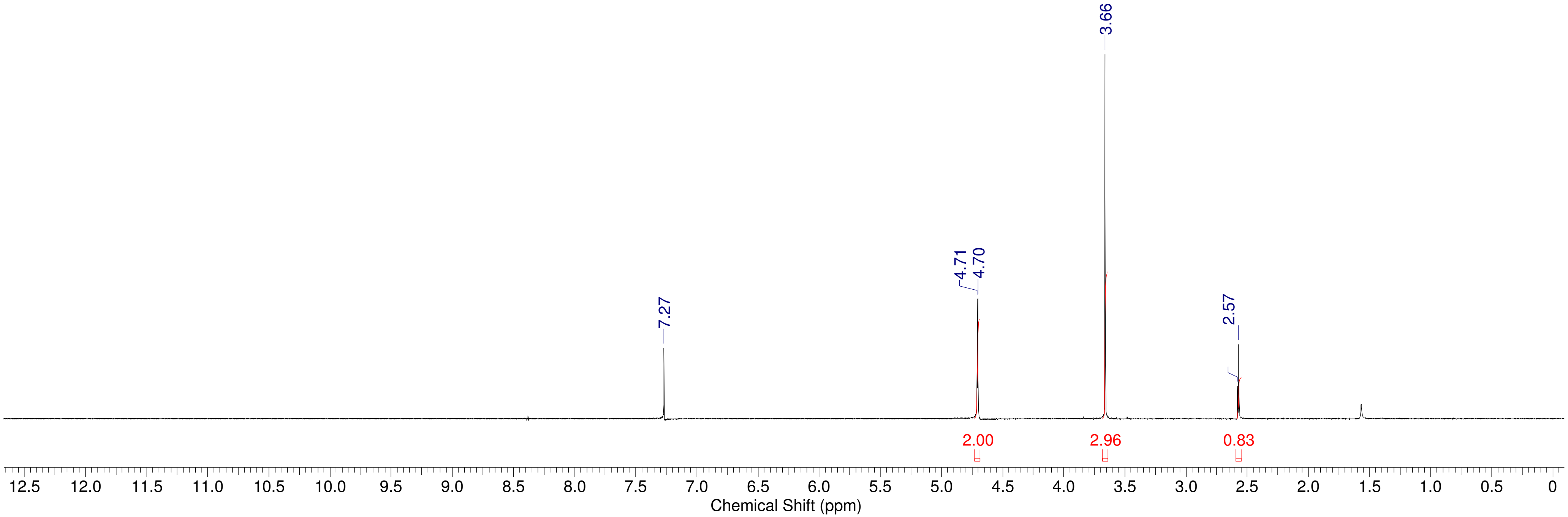

**20** $^{13}$C-NMR ($CDCl_3$, 100 MHz)

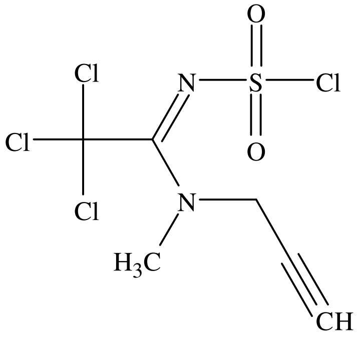

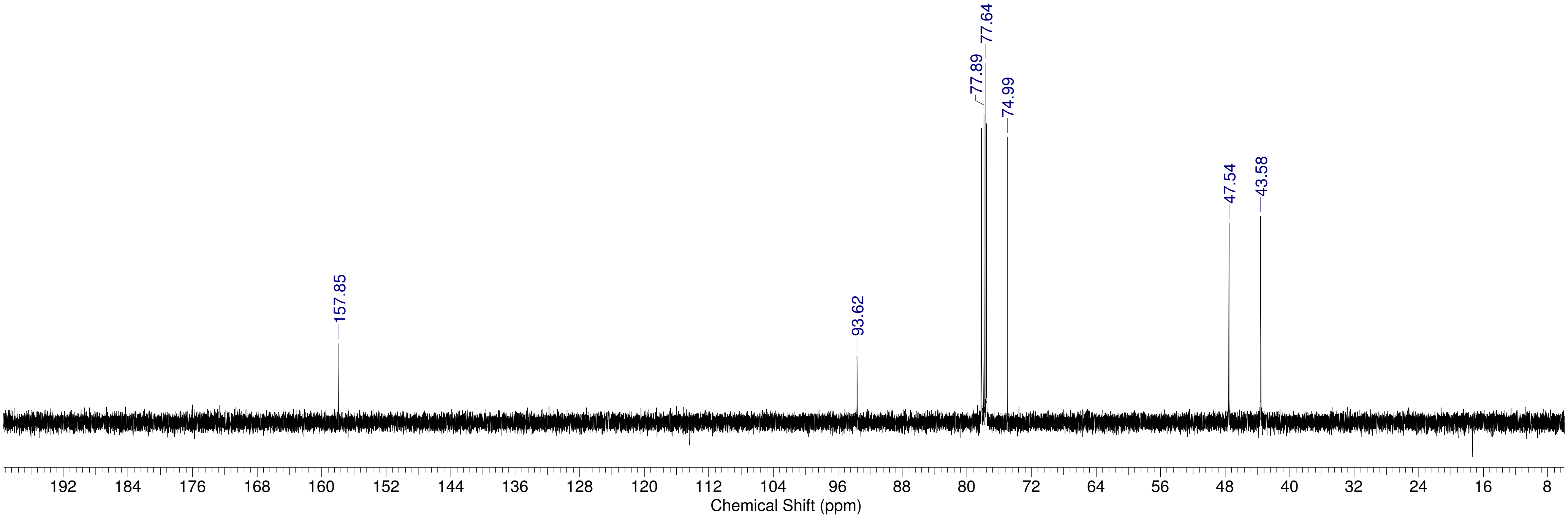

**21** $^{1}$H-NMR ($CDCl_3$, 400 MHz)

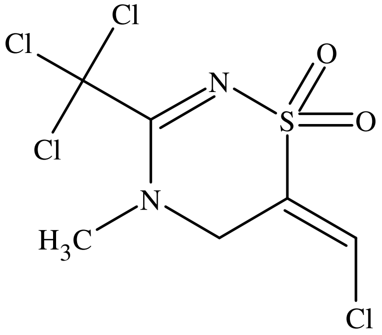


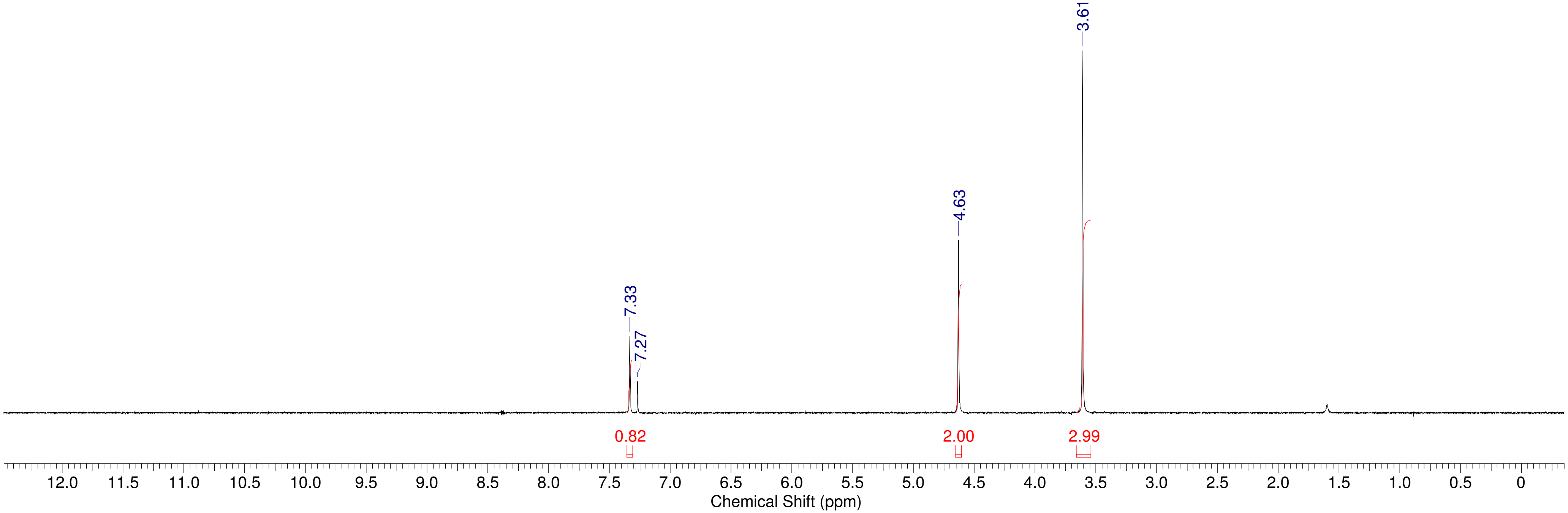

**21** $^{13}$C-NMR (DMSO-d6, 100 MHz)

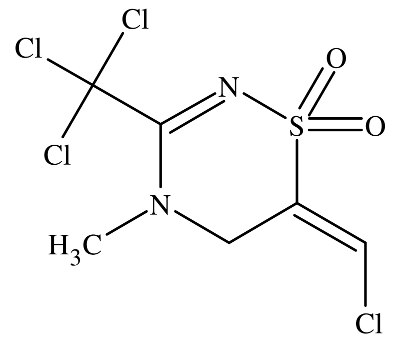


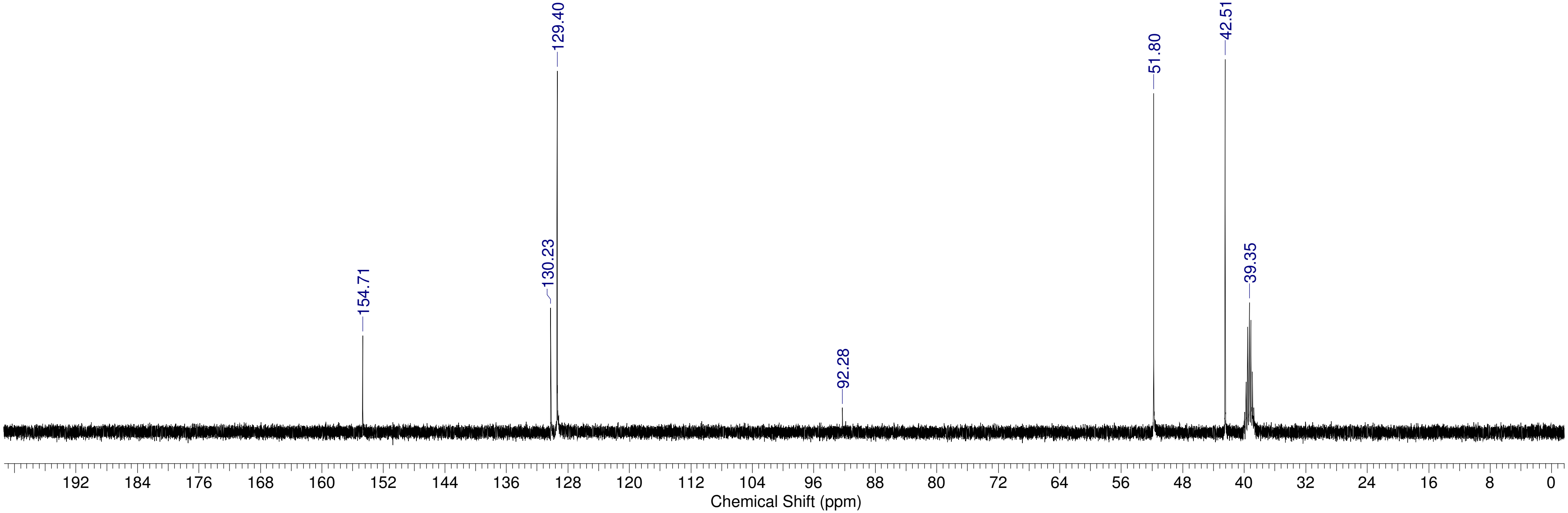

**21** NOE ($CDCl_3$, 600 MHz)

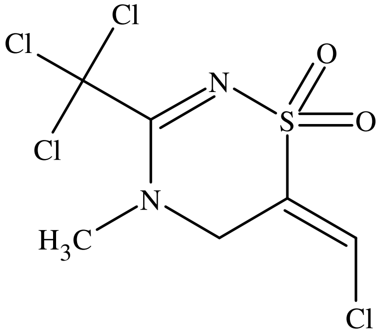


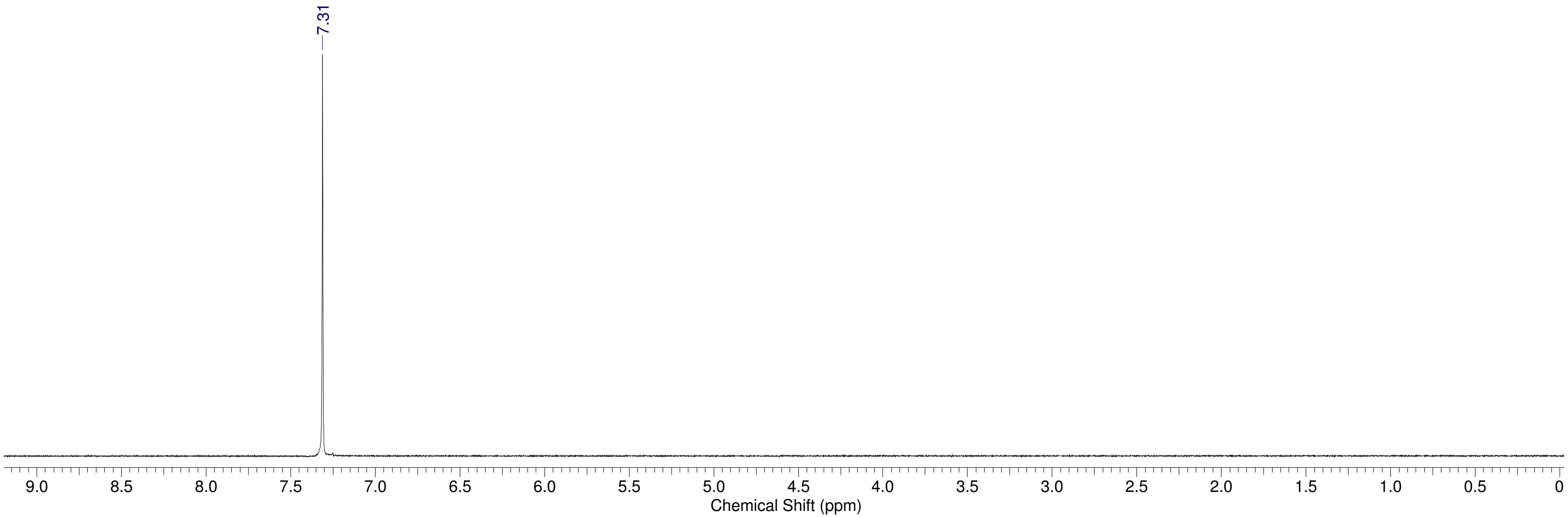

**23** $^1$H-NMR ($CDCl_3$, 600 MHz)

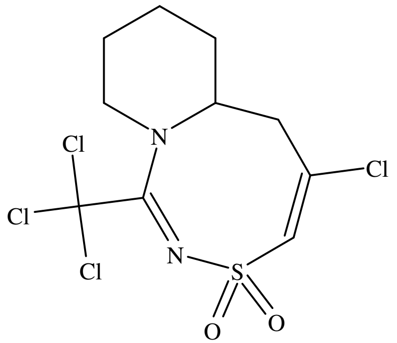


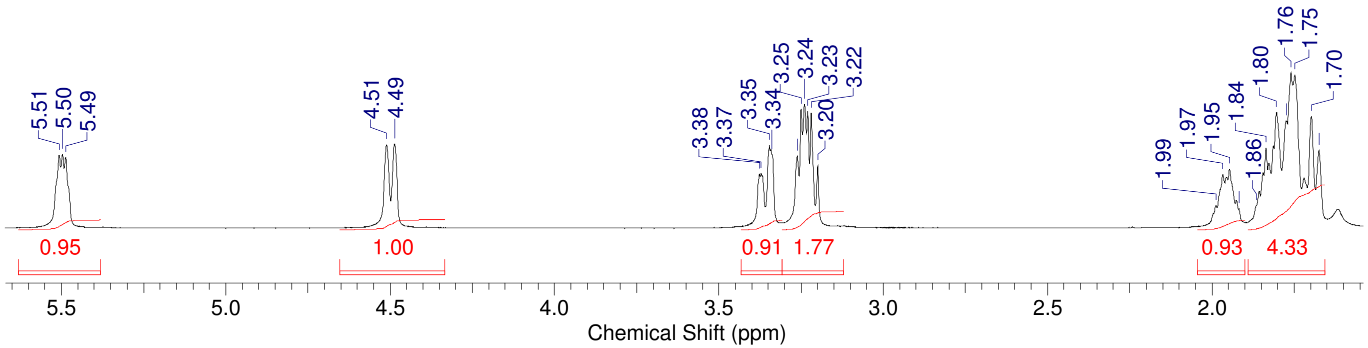


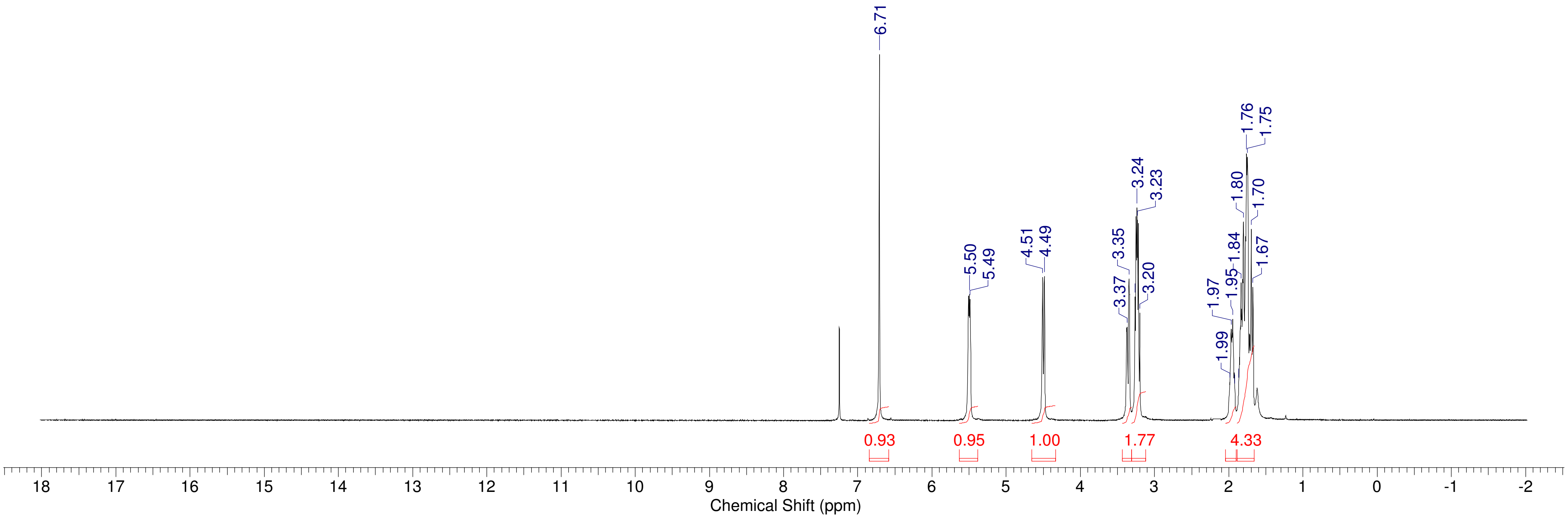

**23** $^{13}$C-NMR (CDCl$_3$, 100 MHz)

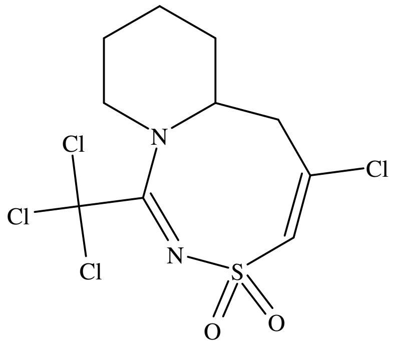


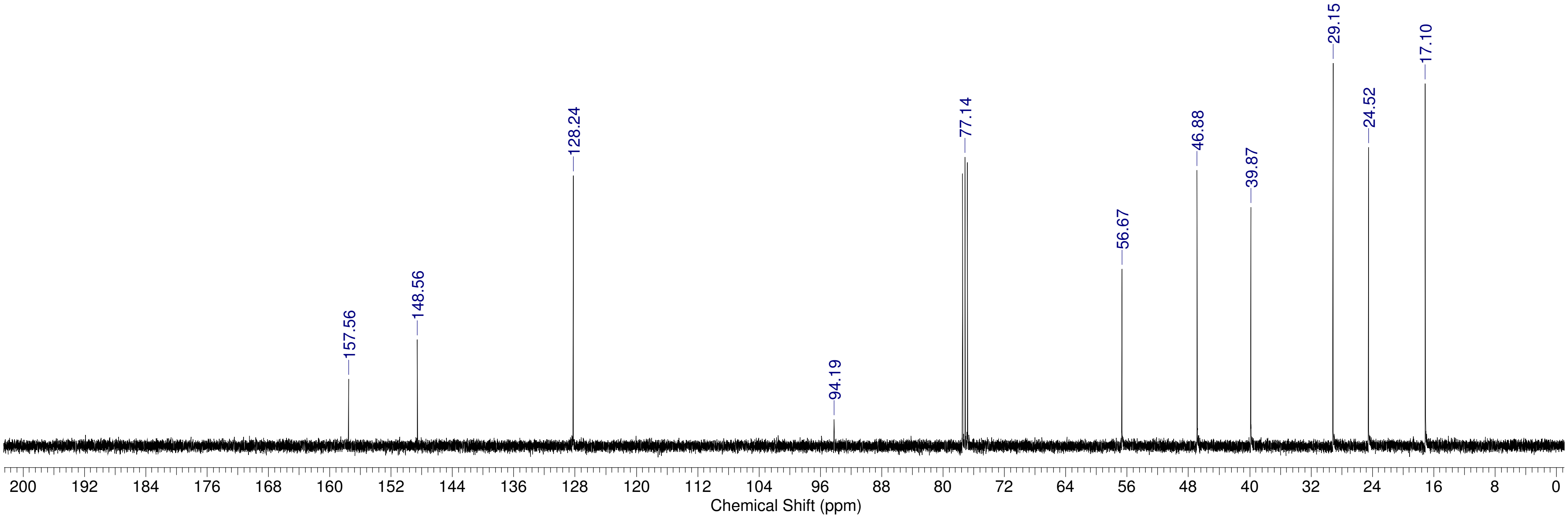

**23** $^{1}$H,$^{1}$H-COSY NMR

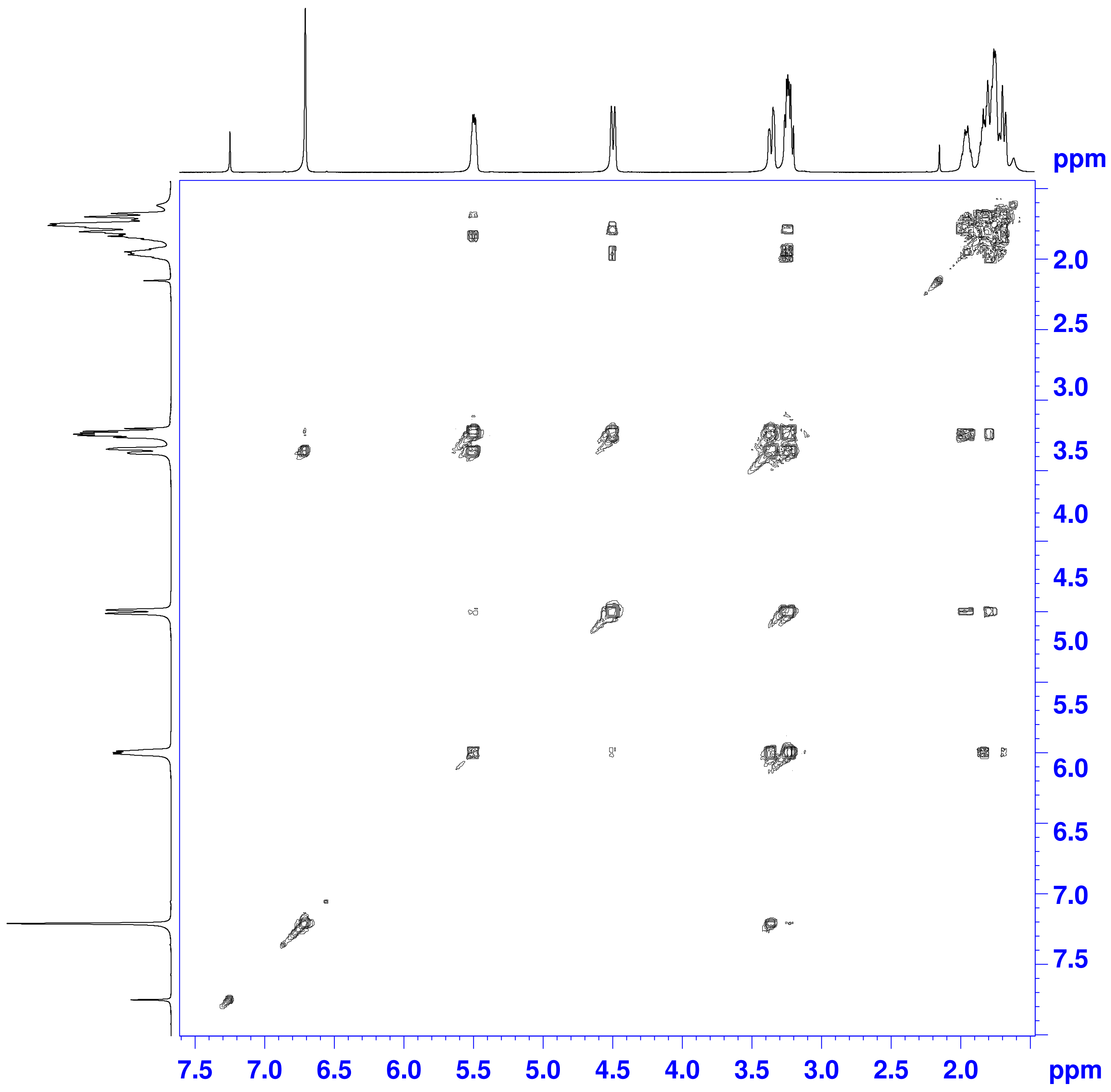


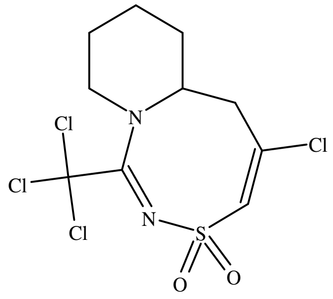

**24** $^1$H-NMR ($CDCl_3$, 600 MHz)

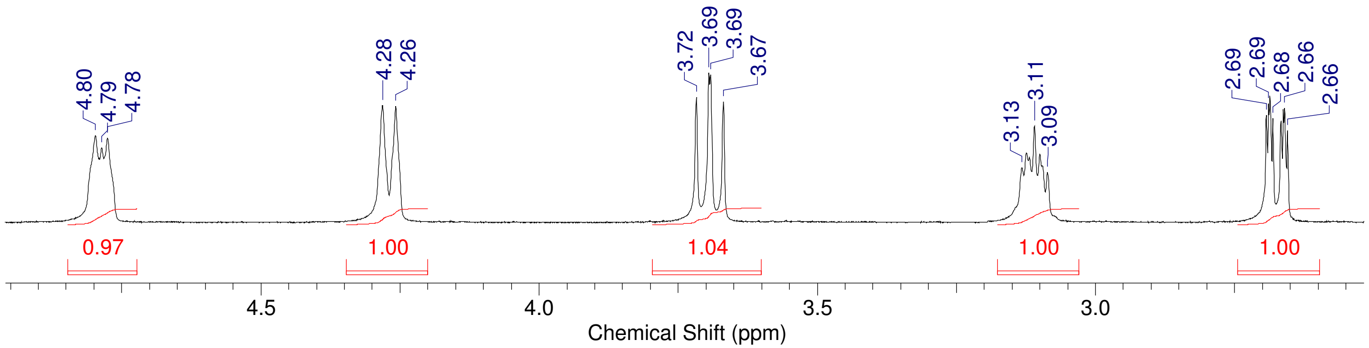

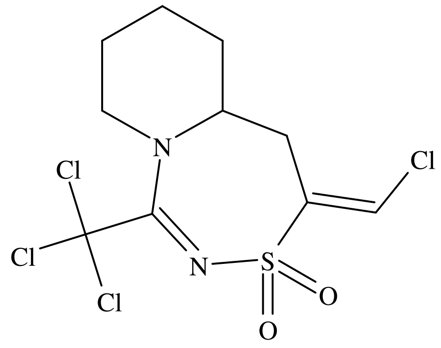

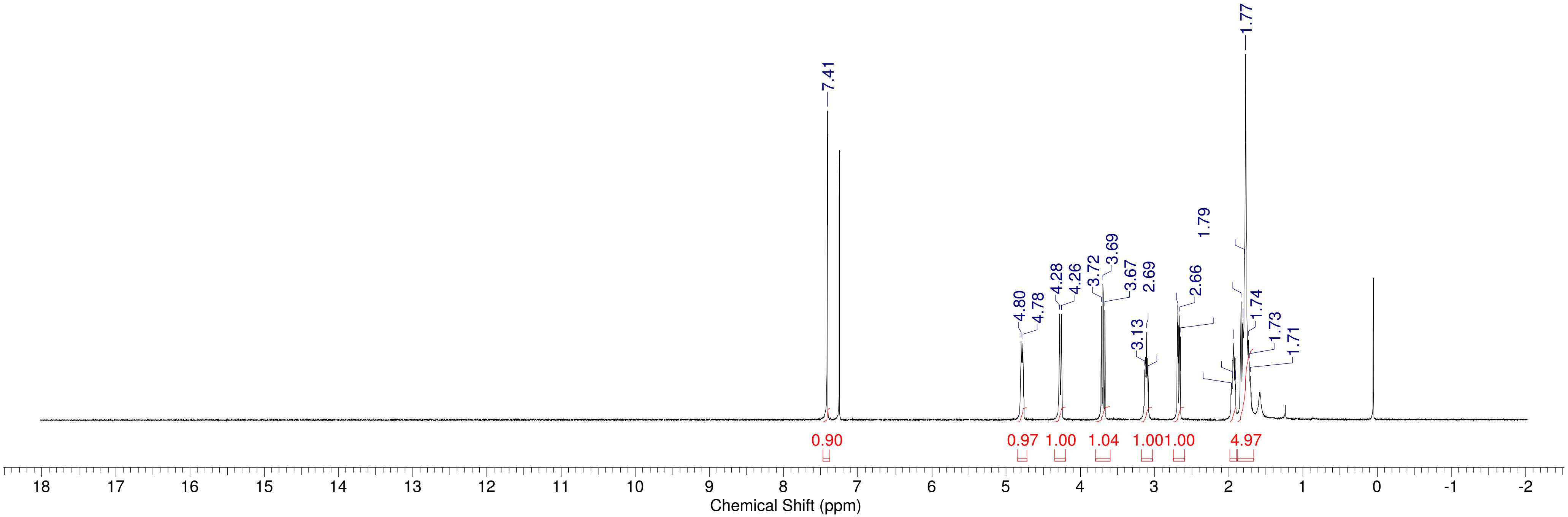

**24** $^{13}$C-NMR (CDCl$_3$, 100 MHz)

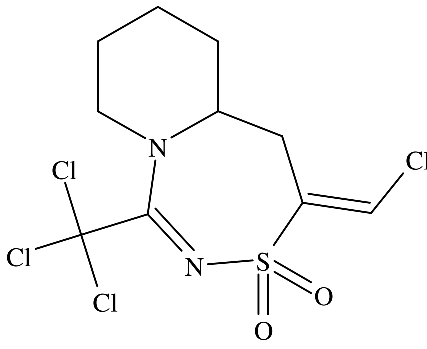


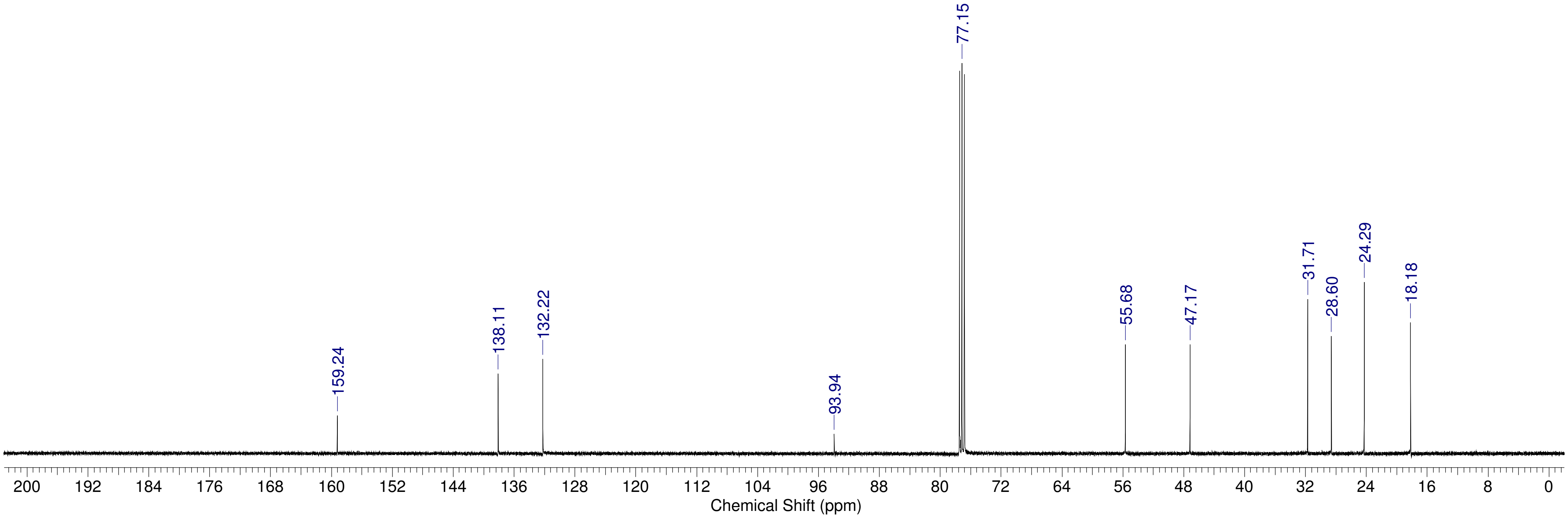

**24** NOE ($CDCl_3$, 600 MHz)

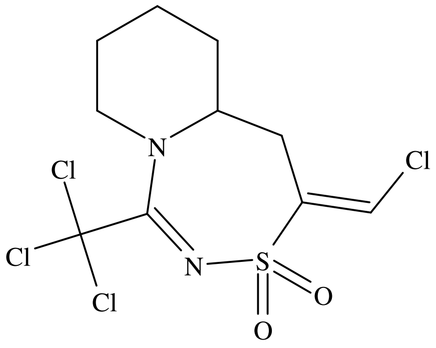


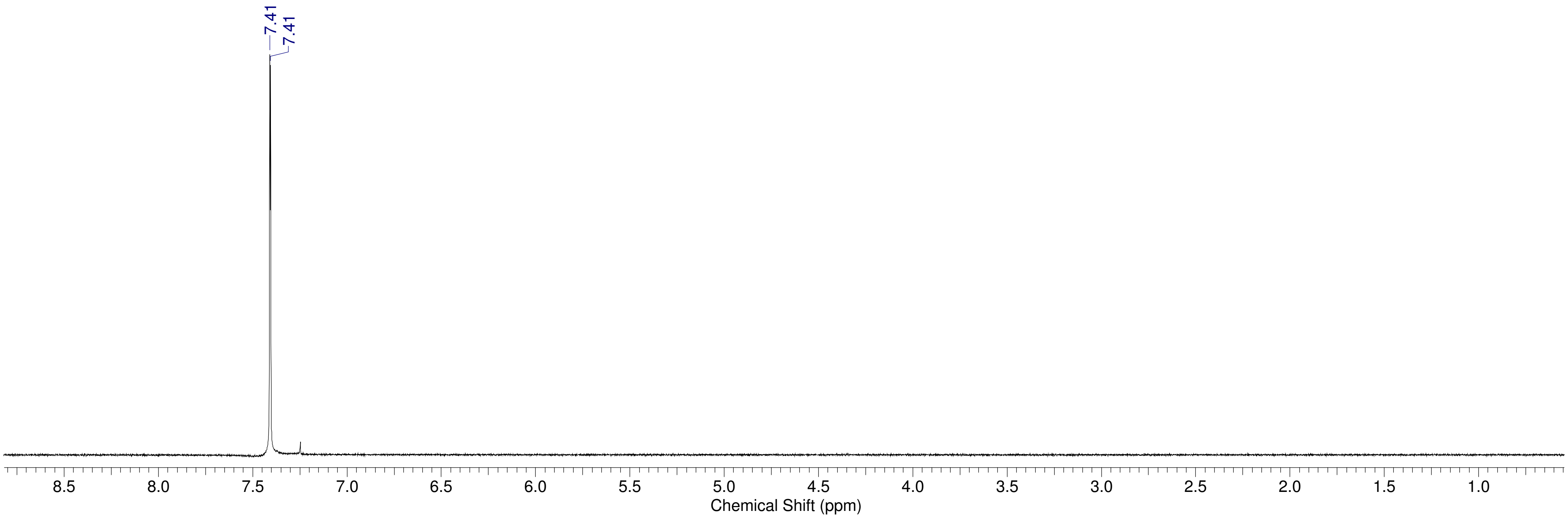

COSY

**24** $^1H,^1H$-COSY NMR

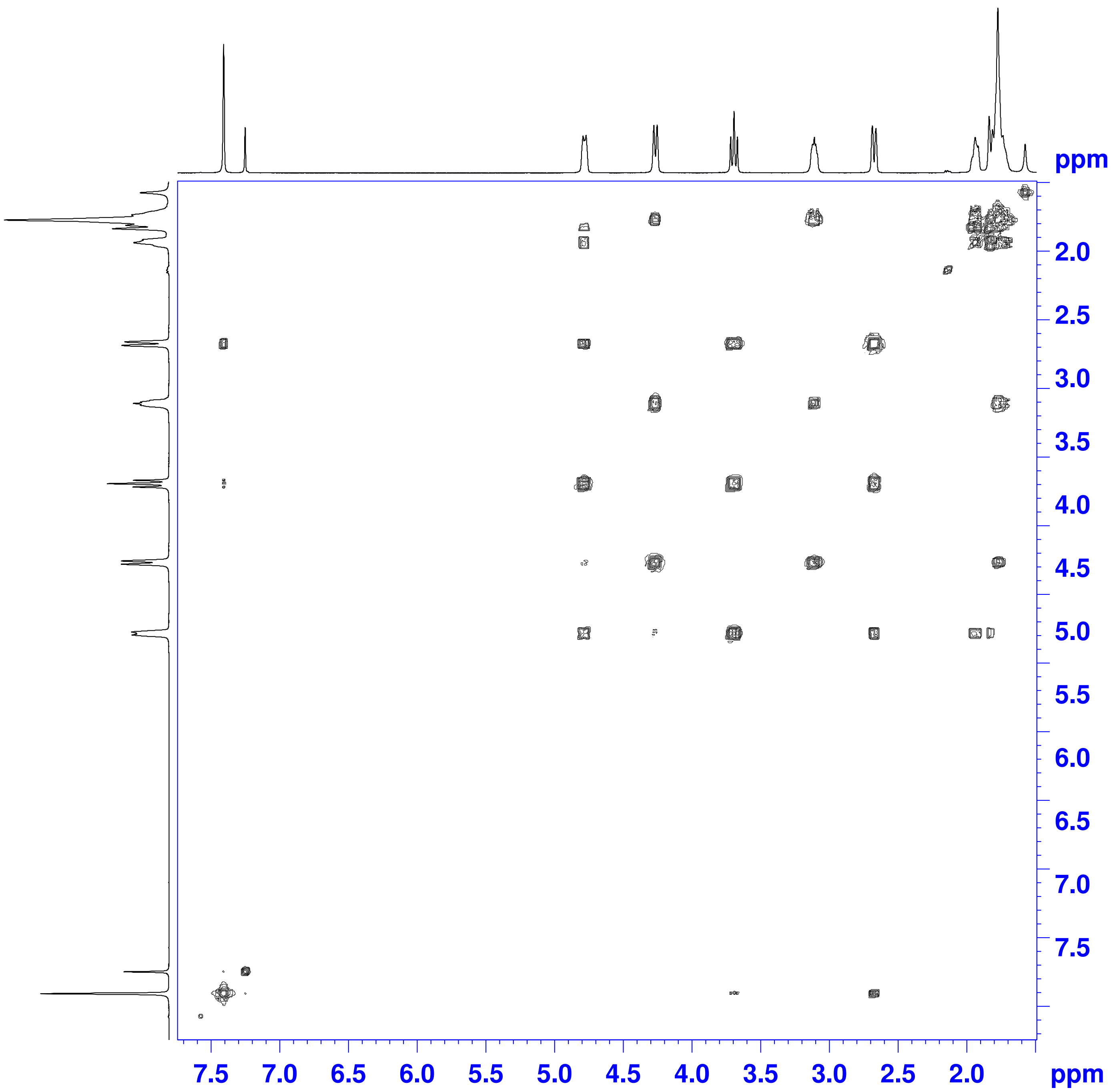


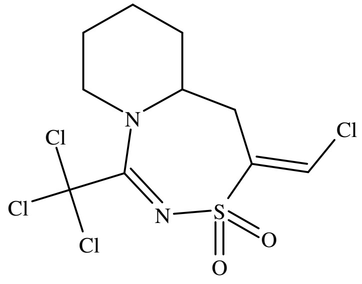

**25** $^1$H-NMR ($CDCl_3$, 600 MHz)

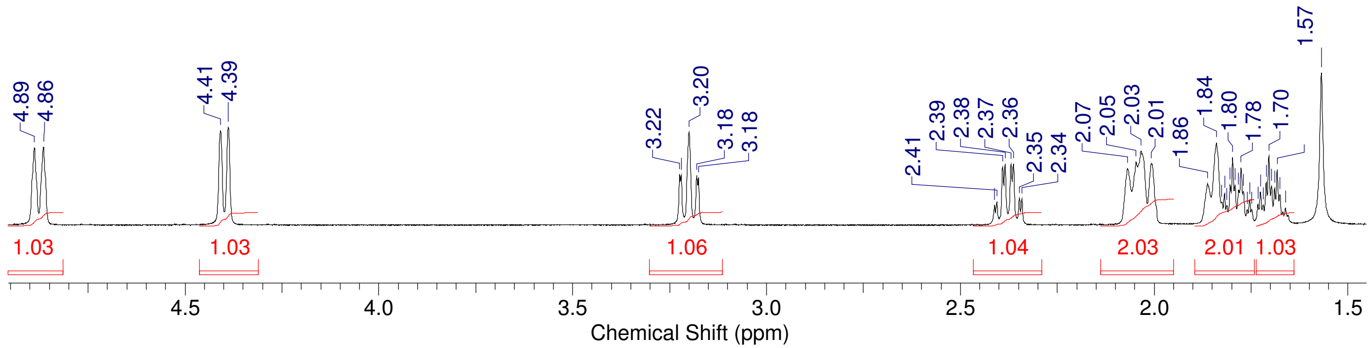

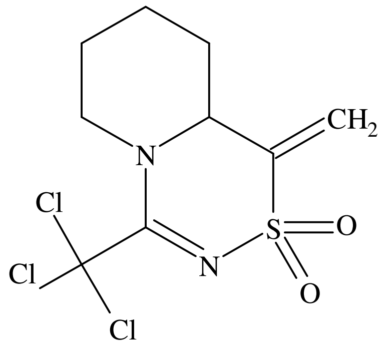


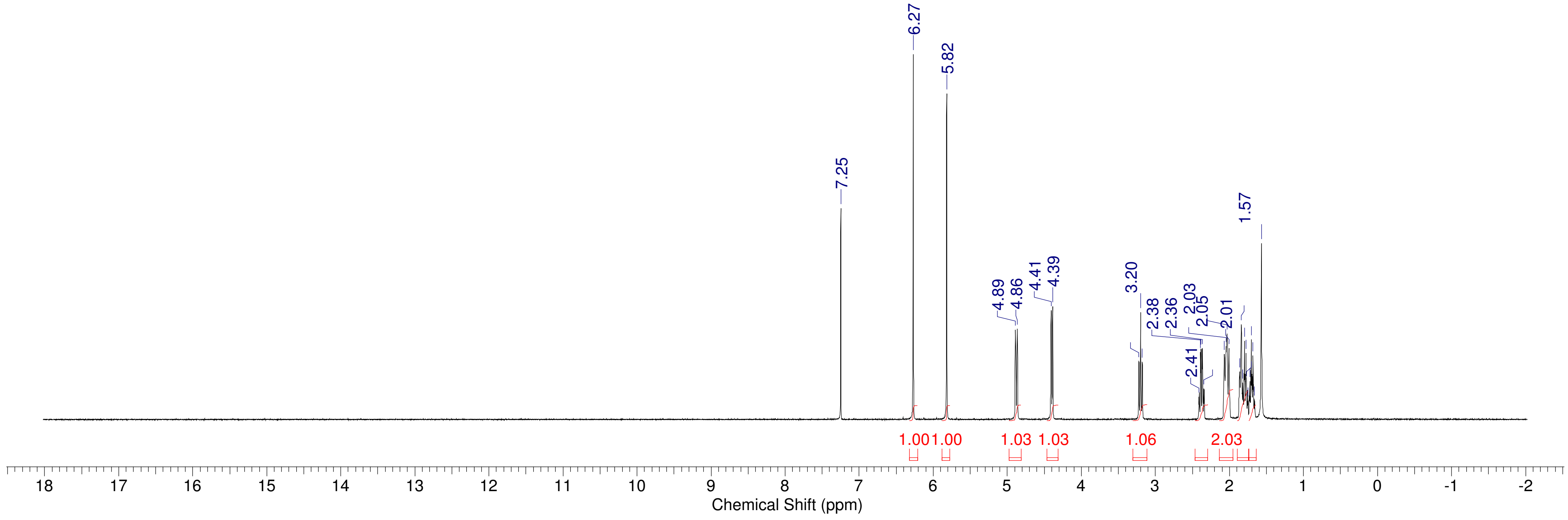

**25** $^{13}$C-NMR (CDCl$_3$, 100 MHz)

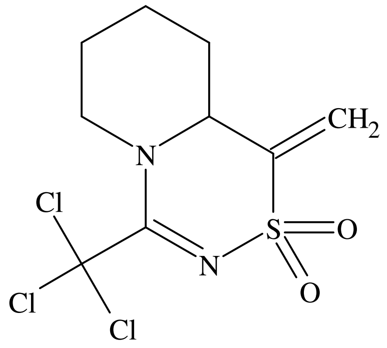

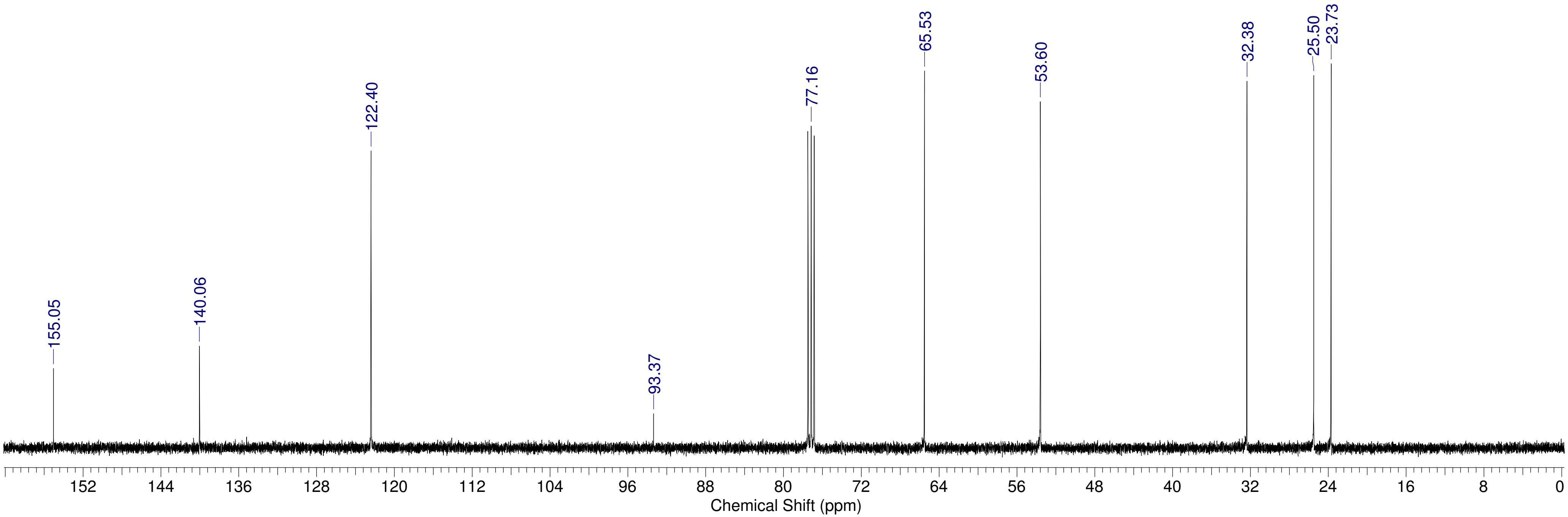

**26** $^1$H-NMR ($CDCl_3$, 600 MHz)

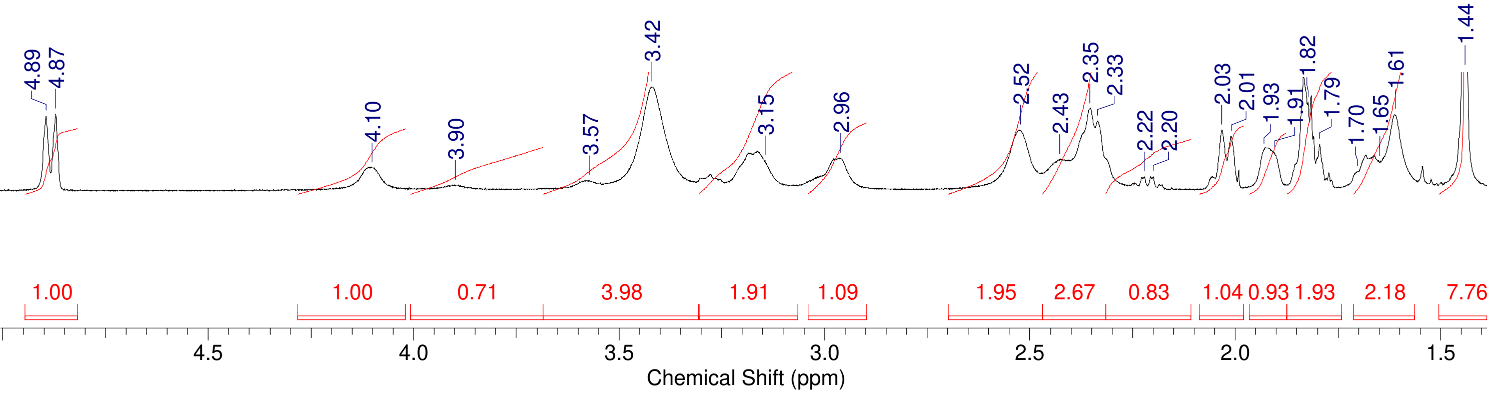


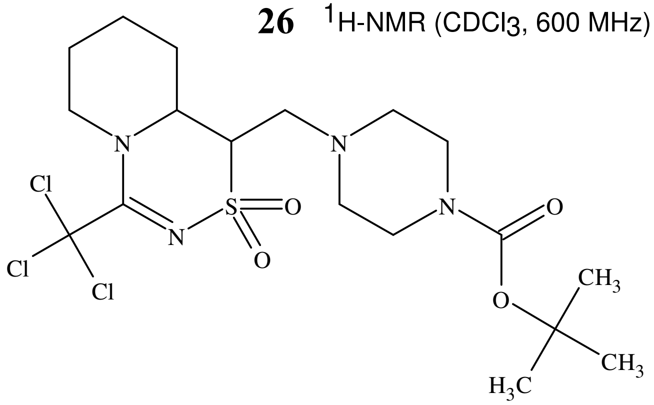


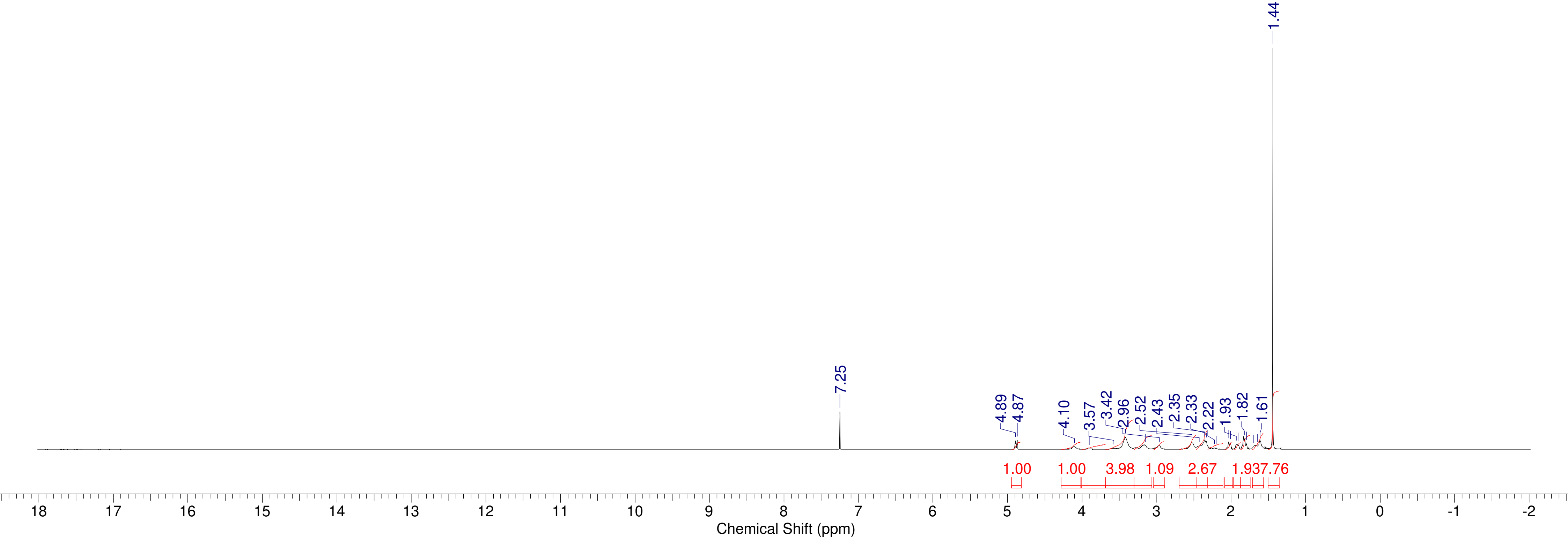

**26** $^{13}$C-NMR ($CDCl_3$, 100 MHz)

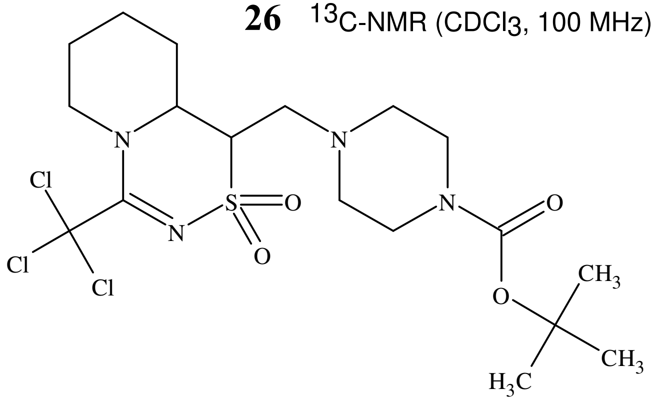


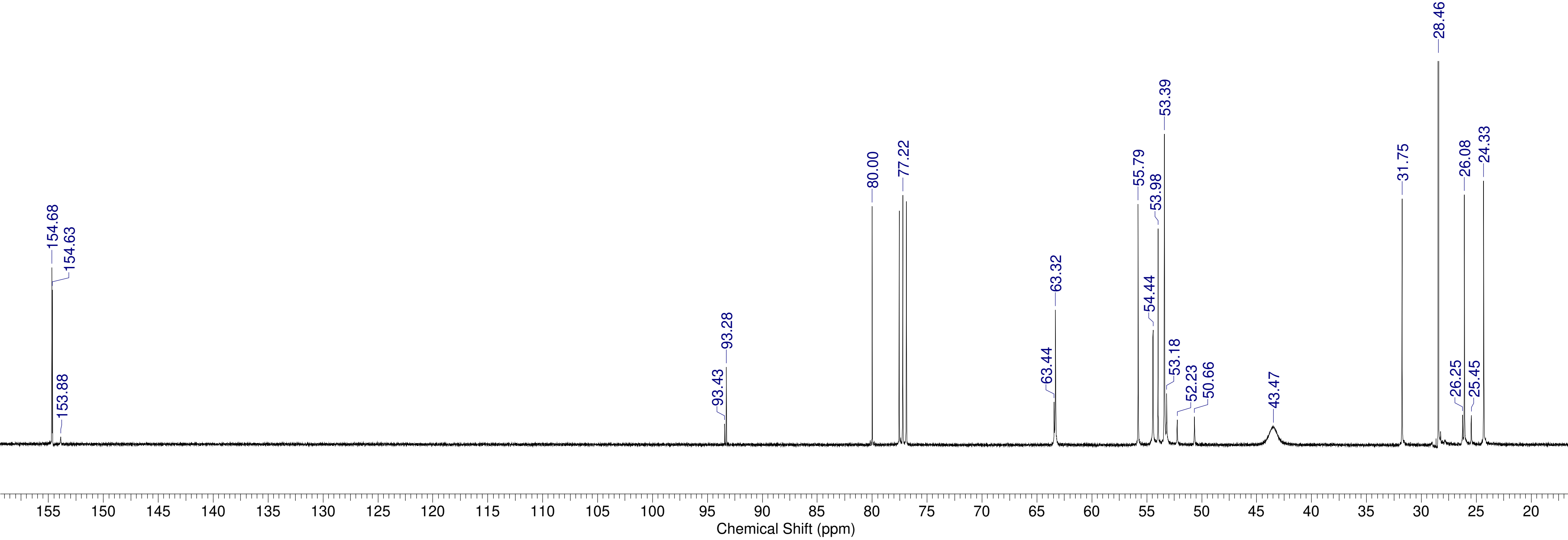

**27** $^1$H-NMR (DMSO-d6, 400 MHz)

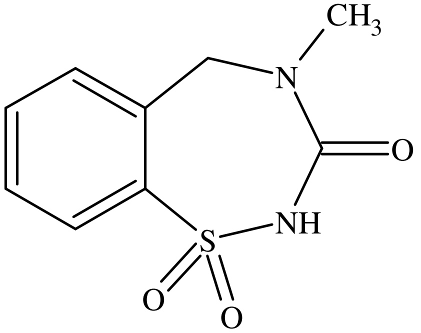

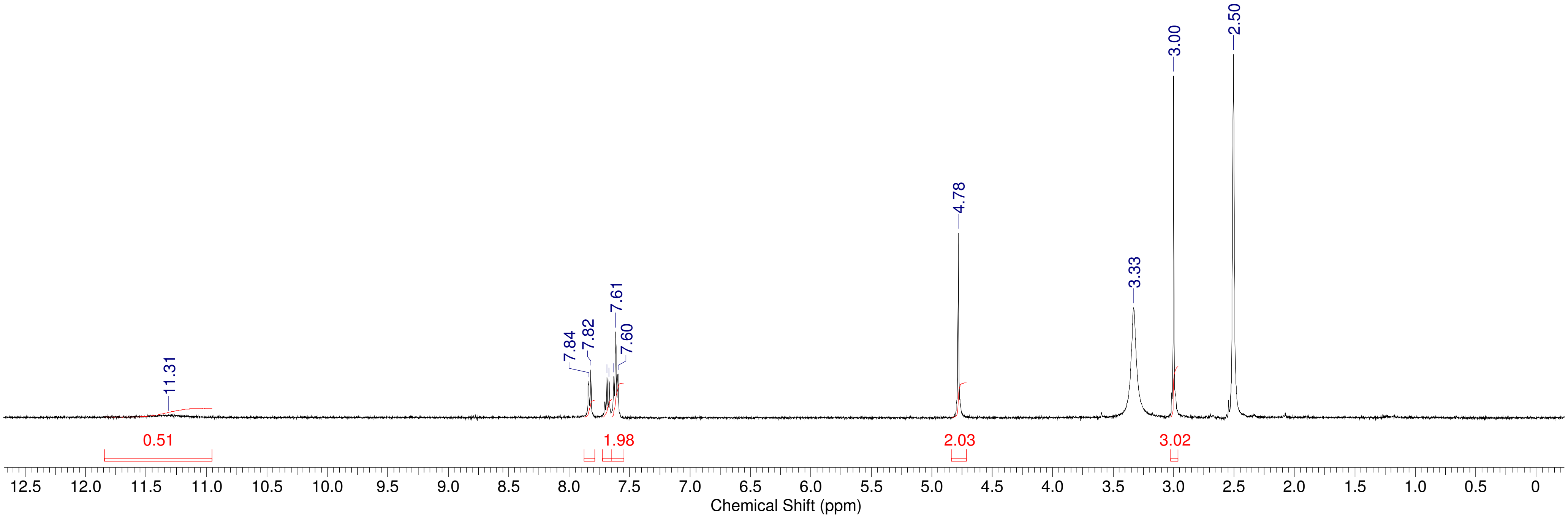

**27** $^{13}C$-NMR (DMSO-d6, 125 MHz)

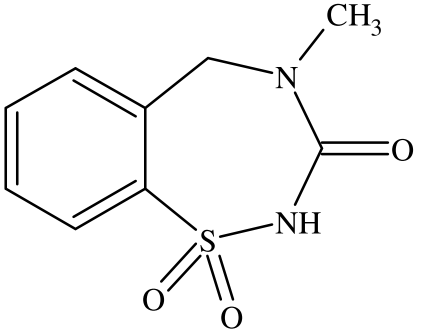


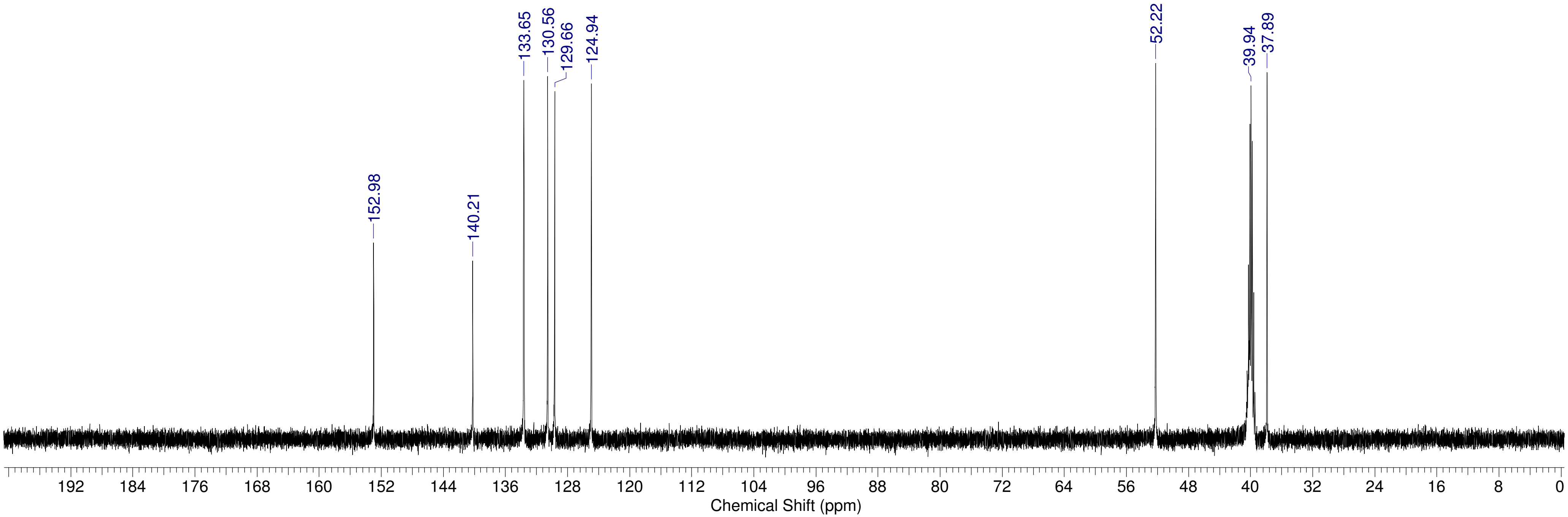

## X-Ray diffraction studies

Crystal data for **4a**: $C_6H_8Cl_4N_2O_2S$, M= 314.02, orthorhombic, space group *Pbca*, $a$ = 11.1206(4), $b$ = 11.3982(4), $c$ = 17.8640(6)Å, V = 2264.35(14)Å$^3$, Z = 8, $d_c$ = 1.842, μ 1.209 mm$^{-1}$, F(000) 1264, crystal size ca. 0.13 x 0.15 x 0.33mm. All crystallographic measurements were performed at ambient temperature on a Bruker Smart Apex II diffractometer operating in the ω scans mode. The intensity data were collected within the $\theta_{max} \leq 26.37°$ using Mo-$K_\alpha$ radiation (λ = 0.71078 Å). The intensities of 29154 reflections were collected (2305 unique reflections, $R_{merg}$ = 0.042). The structure were solved by direct methods and refined by the full-matrix least-squares technique in the anisotropic approximation for non-hydrogen atoms using the Crystals program package [2].
In Structure $CCl_3$ group is disordered over two positions A and B with occupancies 0.60 and 0.40 respectively. All CH hydrogen atoms were placed at calculated positions and refined as 'riding' model. Convergence was obtained at R1 = 0.0397 and wR = 0.0416 for 1775 observed reflections with I ≥ 3σ(I), GOF = 1.1278; R1 = 0.0556 and wR = 0.0508 for 2298 independent reflections, 145 parameters in refinement, the largest and minimal peaks in the final difference map 0.94 and –0.48 e/Å$^3$. Any request to the CCDC for these materials should quote the full literature citation and reference number 2004894. The molecular structure of compound **4a** was shown on fig. 1.

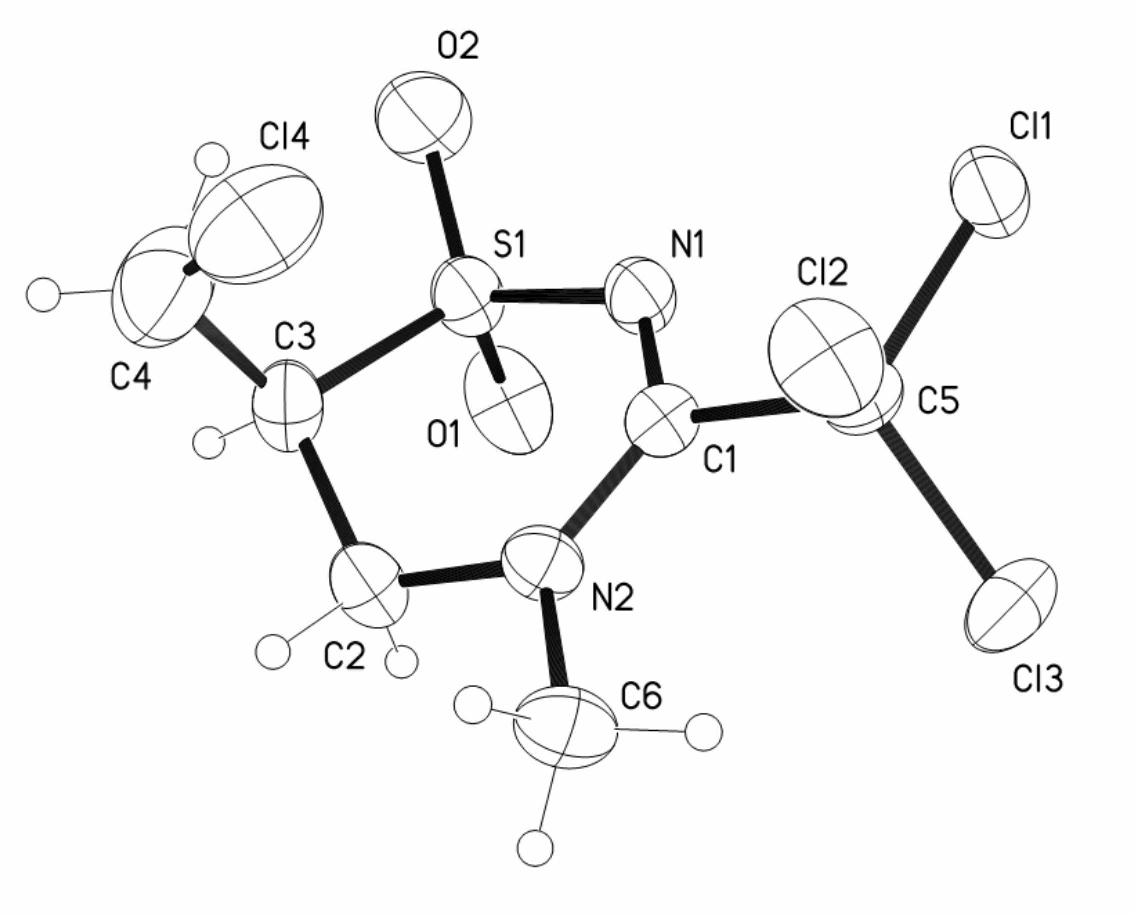


Fig. 1. Molecular structure of compound **4a** including thermal displacement ellipses with 50% probability.

Crystal data for **4b**: $C_5H_6Cl_4N_2O_2S$, M= 299.99, monoclinic, space group *C2/c*, $a$ = 20.549(4), $b$ = 10.2811(18), $c$ = 10.2910(16)Å, $\beta$ = 101.262(8)°, V = 2132.3(6)Å$^3$, Z = 8, $d_c$ = 1.869, μ 1.279 mm$^{-1}$, F(000) 1200, crystal size ca. 0.18 x 0.18 x 0.46mm. All crystallographic measurements were performed at 173K on a Bruker Smart Apex II diffractometer operating in the ω scans mode. The intensity data were collected within the $\theta_{max} \leq 26.43°$ using Mo-$K_\alpha$ radiation (λ = 0.71078 Å). The intensities of 9839 reflections were collected (2191 unique reflections, $R_{merg}$ = 0.055). The structure

were solved by direct methods and refined by the full-matrix least-squares technique in the anisotropic approximation for non-hydrogen atoms using the Crystals program package [2]. All CH hydrogen atoms were placed at calculated positions and refined as 'riding' model. Convergence was obtained at R1 = 0.1210 and wR = 0.1077 for 1741 observed reflections with I ≥ 3σ(I), GOF = 0.9745; R1 = 0.1337 and wR = 0.1145 for 2182 independent reflections, 131 parameters in refinement, the largest and minimal peaks in the final difference map 1.39 and –1.53 e/Å$^3$. Any request to the CCDC for these materials should quote the full literature citation and reference number 2004895. The molecular structure of compound **4b** was shown on fig. 2

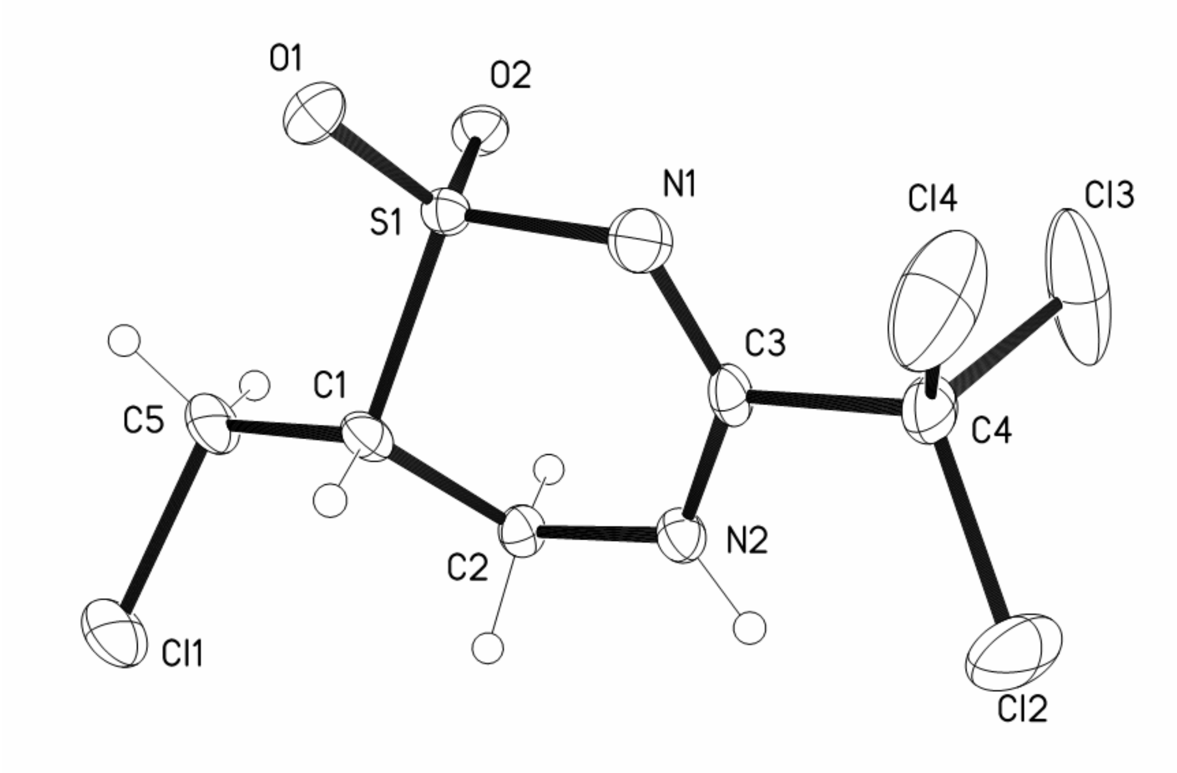


Fig. 2. Molecular structure of compound **4b** including thermal displacement ellipses with 50% probability.

Crystal data for compound **4d**: $C_{11}H_{10}Cl_4N_2O_2S$ x $CHCl_3$, M = 495.44, hexagonal, space group *P6$_5$*, *a* = 16.3287(13), *c* = 10.7866(10)Å, V = 2490.7(5)Å$^3$, Z = 6, d$_c$ = 1.982 g·cm$^{-3}$, μ = 1.331 mm$^{-1}$, F(000) = 1488, crystal size ca. 0.07 × 0.07 × 0.55 mm. All crystallographic measurements were performed at 173K on a Bruker Smart Apex II diffractometer operating in the ω scans mode. The intensity data of 16014 reflection were collected within the range of 1.44 ≤ θ ≤ 26.33° using Mo-K$_α$ radiation (λ = 0.71078 Å, 3403 unique reflections, R$_{merg}$ = 0.0785). The structure were solved by direct methods and refined by the full-matrix least-squares technique in the anisotropic approximation for non-hydrogen atoms using the Bruker SHELXTL program package [1]. The solvate $CHCl_3$ molecule could not be modeled satisfactorily thus SQEESE [3] routine in the PlLATON [4], [5] software were applied for correction of the data. All CH hydrogen atoms were placed at calculated positions and refined as 'riding' model. Convergence was obtained at R1 = 0.0553 and wR2 = 0.1186 for 2580 observed reflections with I ≥ 2σ(I), R1 = 0. 0742 and wR2 = 0.1272, GOF = 0.974 for 3403 independent reflections, 185 parameters, the Flack parameter is -0.08(9), the largest and minimal peaks in the final difference map 0.38 and –0.31 e/Å$^3$. Full crystallographic details have been deposited at Cambridge Crystallographic Data Centre (CCDC). Any request to the CCDC for these materials should quote the full literature citation and reference number CCDC 2004896. The molecular structure of compound **4d** was shown on fig. 3.

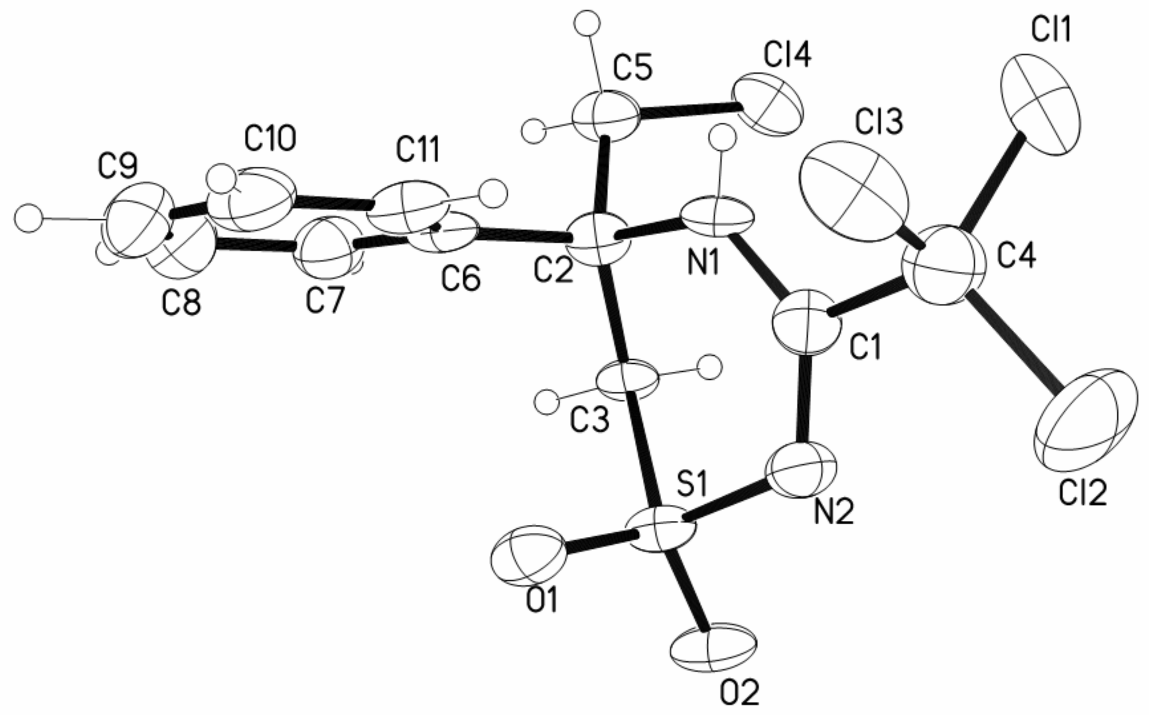


Fig. 3. Molecular structure of compound **4d** without $CHCl_3$ molecule including thermal displacement ellipses with 50% probability.

Crystal data for compound **7**: $C_9H_{12}Cl_4N_2O_2S$ , M = 354.07, monoclinic, space group $P2_1/n$, $a$ = 9.1111(10), $b$ = 13.6390(14), $c$ = 11.6642(14)Å, $\beta$ = 109.114(2)° V = 1369.6(3)$Å^3$, Z = 4, $d_c$ = 1.717 g·cm$^{-3}$, $\mu$ = 1.010 mm$^{-1}$, F(000) = 720, crystal size ca. 0.25 × 0.31 × 0.33 mm. All crystallographic measurements were performed at ambient temperature on a Bruker Smart Apex II diffractometer operating in the ω scans mode. The intensity data were collected within the range of 2.4 ≤ θ ≤ 28.5° using Mo-$K_\alpha$ radiation (λ = 0.71078 Å). The intensities of 24708 reflections were collected (3447 unique reflections, $R_{merg}$ = 0.0378). The structure were solved by direct methods and refined by the full-matrix least-squares technique in the anisotropic approximation for non-hydrogen atoms using the Bruker SHELXTL program package [1]. In Structure $CH_2Cl$ group is disordered over two positions A and B with occupancies 0.82 and 0.18 respectively. All CH hydrogen atoms were placed at calculated positions and refined as 'riding' model. Convergence was obtained at R1 = 0.0334 and wR2 = 0.0815 for 586 observed reflections with I ≥ 2σ(I), R1 = 0. 0412 and wR2 = 0.0871, GOF = 1.038 for 2974 independent reflections, 188 parameters, the largest and minimal peaks in the final difference map 0.70 and –0.43 e/$Å^3$. Full crystallographic details have been deposited at Cambridge Crystallographic Data Centre (CCDC). Any request to the CCDC for these materials should quote the full literature citation and reference number CCDC 2004897. The molecular structure of compound **7** was shown on fig. 4.

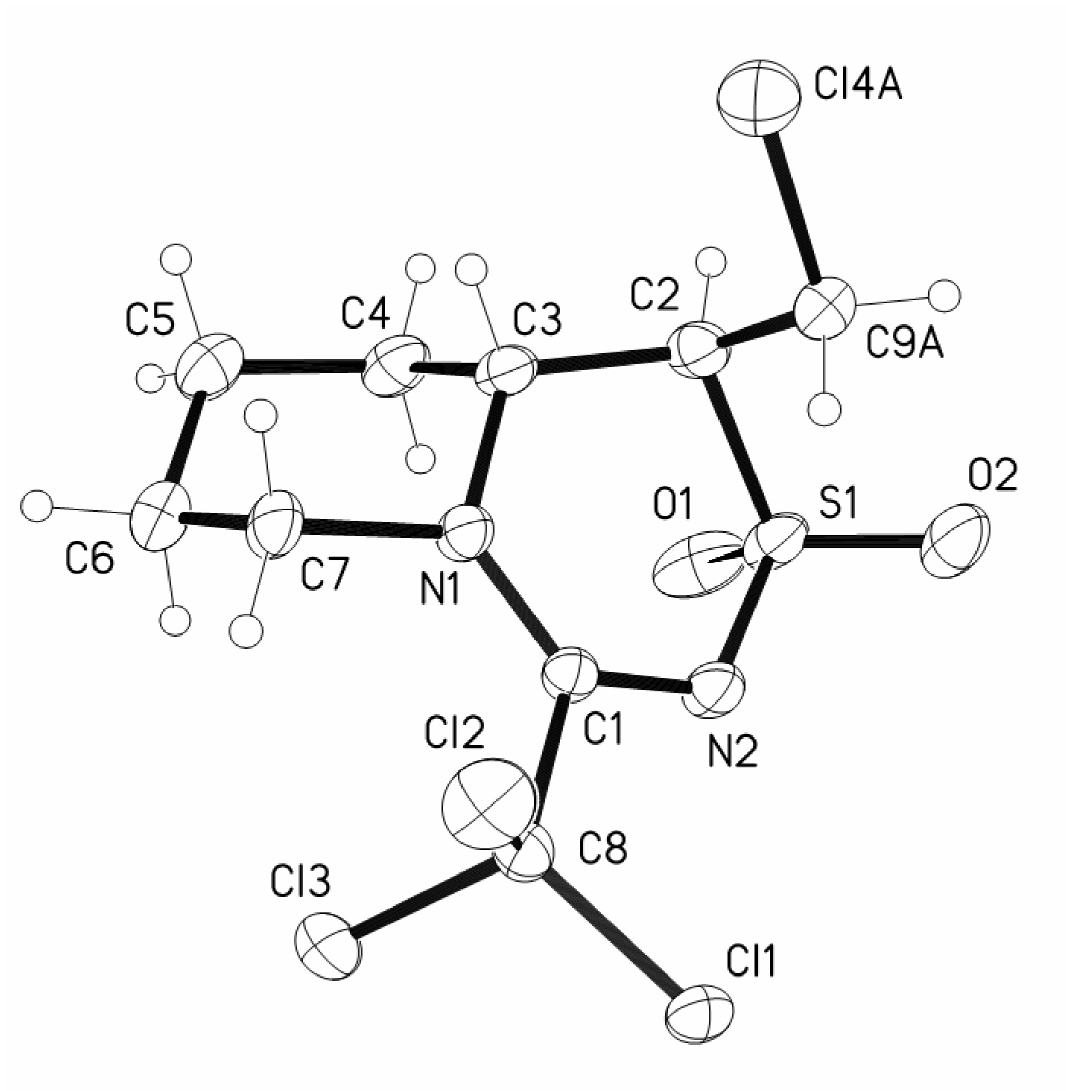


Fig. 4. Molecular structure of compound **7** including thermal displacement ellipses with 50% probability.

Crystal data for **10**: $C_9H_{12}Cl_4N_2O_2S$, M= 354.07, monoclinic, space group $P2_1/n$, *a* = 6.9003(8), *b* = 9.9339(13), *c* = 20.181(3)Å, *β* = 99.395(4)°, V = 1364.8(3)Å$^3$, Z = 4, $d_c$ = 1.723, μ 1.014 mm$^{-1}$, F(000) 720, crystal size ca. 0.13 x 0.15 x 0.33mm. All crystallographic measurements were performed at 173K on a Bruker Smart Apex II diffractometer operating in the ω scans mode. The intensity data were collected within the $\theta_{max} \leq 26.04°$ using Mo-$K_\alpha$ radiation (λ = 0.71078 Å). The intensities of 7422 reflections were collected (2647 unique reflections, $R_{merg}$ = 0.0447). The structure were solved by direct methods and refined by the full-matrix least-squares technique in the anisotropic approximation for non-hydrogen atoms using the Bruker SHELXTL program package [1]. In structure **10** chlorine atoms of the $CCl_3$ group is disordered over two positions A and B with occupancies 0.60 and 0.40 respectively. All CH hydrogen atoms were placed at calculated positions and refined as 'riding' model. Convergence was obtained at R1 = 0.0598 and wR2 = 0.1468 for 1971 observed reflections with I ≥ 2σ(I); R1 = 0.0854 and wR2 = 0.1577, GOF = 1.058 for 2647 independent reflections, 195 parameters, the largest and minimal peaks in the final difference map 0.57 and –0.33 e/Å$^3$. Any request to the CCDC for these materials should quote the full literature citation and reference number 2004898. The molecular structure of compound **10** was shown on fig. 5.

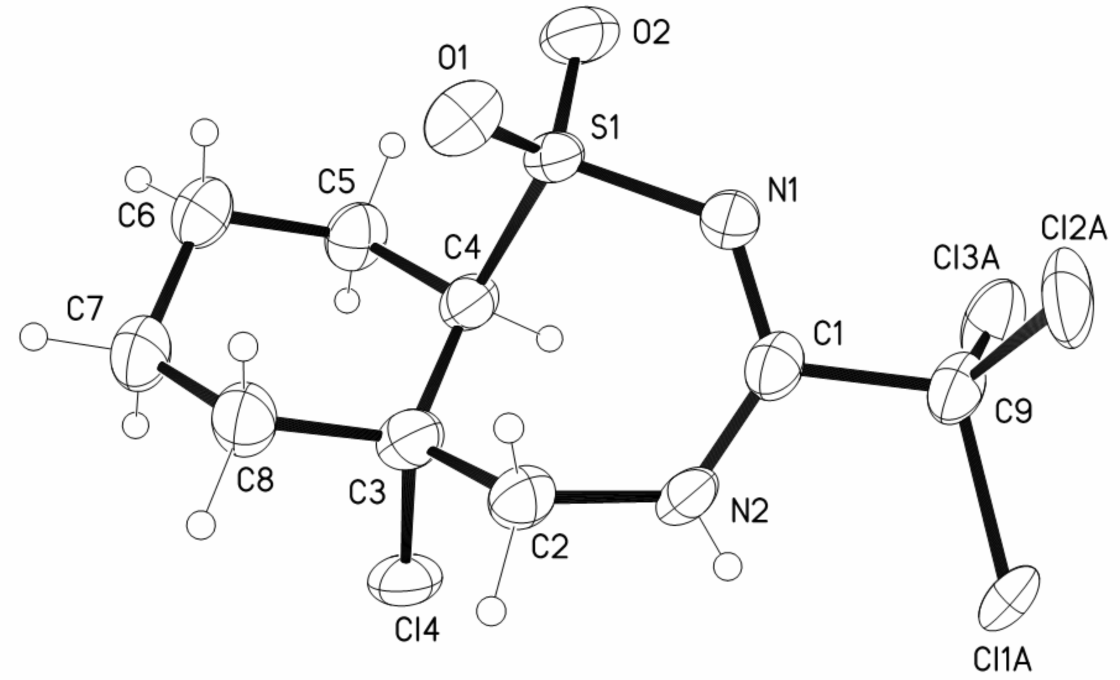


Fig. 5. Molecular structure of compound **10** including thermal displacement ellipses with 50% probability.

Crystal data for **12**: $C_7H_{10}Cl_4N_2O_2S$, M= 328.03, orthorhombic, space group *Pbca*, *a* = 12.680(3), *b* = 11.655(3), *c* = 16.678(4)Å, V = 2464.6(11)Å$^3$, Z = 8, $d_c$ = 1.768, μ 1.115 mm$^{-1}$, F(000) 1328, crystal size ca. 0.22 x 0.26 x 0.38mm. All crystallographic measurements were performed at ambient temperature on a Bruker Smart Apex II diffractometer operating in the ω scans mode. The intensity data were collected within the $\theta_{max} \leq 26.5°$ using Mo-$K_\alpha$ radiation (λ = 0.71078 Å). The intensities of 23484 reflections were collected (2544 unique reflections, $R_{merg}$ = 0.1289). The structure were solved by direct methods and refined by the full-matrix least-squares technique in the anisotropic approximation for non-hydrogen atoms using the Bruker SHELXTL program package[1]. All CH hydrogen atoms were placed at calculated positions and refined as 'riding' model. Convergence was obtained at R1 = 0.0537 and wR2 = 0.1190 for 1473 observed reflections with I ≥ 2σ(I), R1 = 0. 1063 and wR2 = 0.1438, GOF = 0.989 for 2544 independent reflections, 147 parameters, the largest and minimal peaks in the final difference map 0.38 and –0.41 e/Å$^3$. Full crystallographic details have been deposited at Cambridge Crystallographic Data Centre (CCDC). Any request to the CCDC for these materials should quote the full literature citation and reference number CCDC 2004905. Any request to the CCDC for these materials should quote the full literature citation and reference number 2005693. The molecular structure of compound [12] was shown on fig. 6.

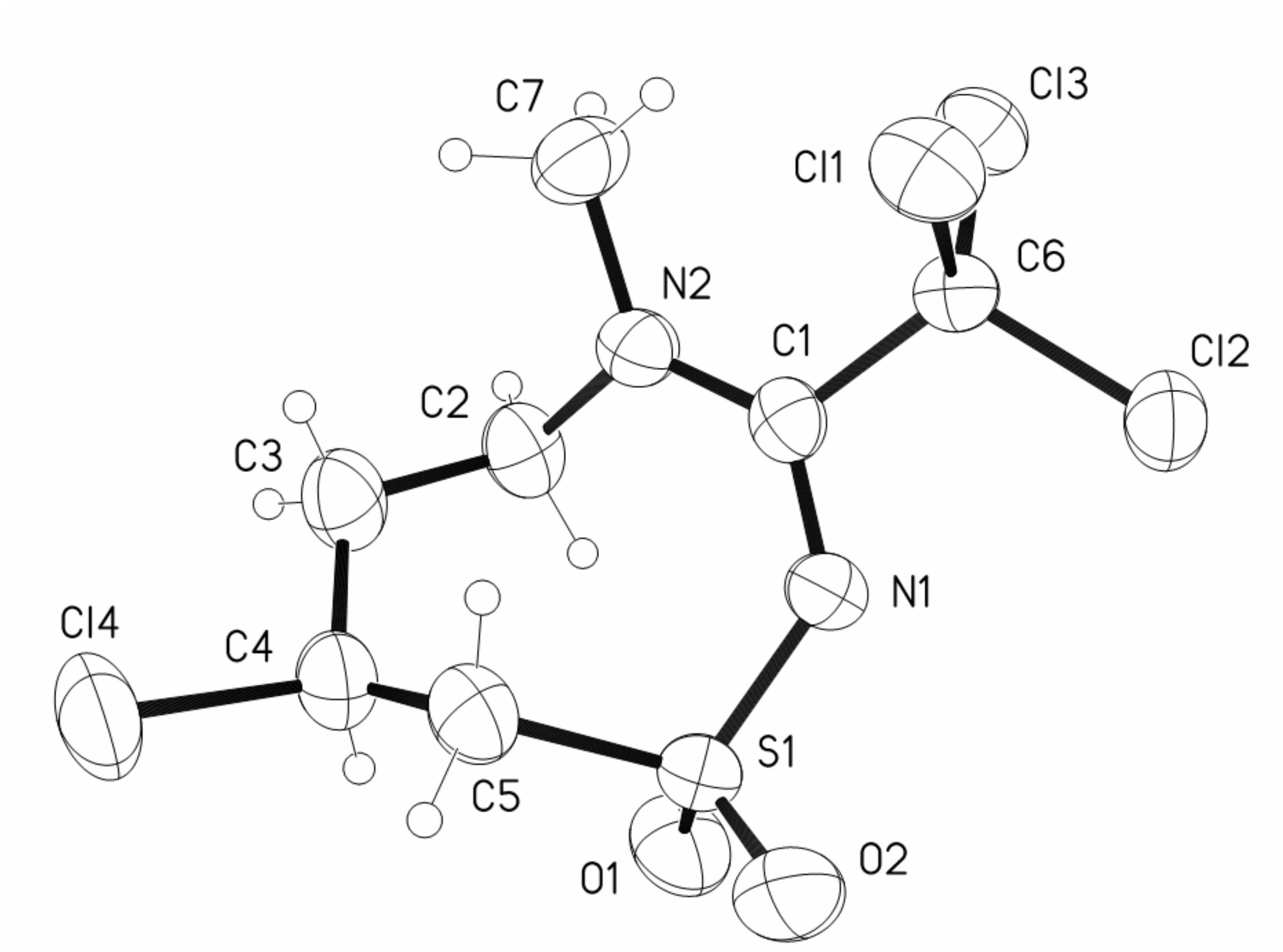


Fig. 6. Molecular structure of compound **12** including thermal displacement ellipses with 50% probability.

Crystal data for compound **14** $C_{10}H_{14}Cl_4N_2O_2S$ x $0.5(C_6H_6)$, M = 407.15, monoclinic, space group *P2₁/c*,, $a$ = 12.715(5), $b$ = 9.002(4), $c$ = 15.998(7)Å, $\beta$ = 110.07(3)°, V = 1720.0(13)Å$^3$, Z = 4, $d_c$ = 1.572 g·cm$^{-3}$, $\mu$ = 0.816 mm$^{-1}$, F(000) = 836, crystal size ca. 0.11 × 0.17 × 0.29 mm. All crystallographic measurements were performed at ambient temperature on a Bruker Smart Apex II diffractometer operating in the ω scans mode. The intensity data were collected within the range of 2.72 ≤ θ ≤ 26.37° using Mo-$K_\alpha$ radiation (λ = 0.71078 Å). The intensities of 17230 reflections were collected (3567 unique reflections, $R_{merg}$ = 0.0663). The structure were solved by direct methods and refined by the full-matrix least-squares technique in the anisotropic approximation for non-hydrogen atoms using the Bruker SHELXTL program package [1]. All CH hydrogen atoms were placed at calculated positions and refined as 'riding' model. Convergence was obtained at R1 = 0.0450 and wR2 = 0.0978 for 2526 observed reflections with I ≥ 2σ(I), R1 = 0. 0744 and wR2 = 0.1121, GOF = 1.011 for 3567 independent reflections, 199 parameters, the largest and minimal peaks in the final difference map 0.29 and –0.24 e/Å$^3$. Full crystallographic details have been deposited at Cambridge Crystallographic Data Centre (CCDC). Any request to the CCDC for these materials should quote the full literature citation and reference number CCDC 2004899. The molecular structure of compound **14** was shown on fig. 7

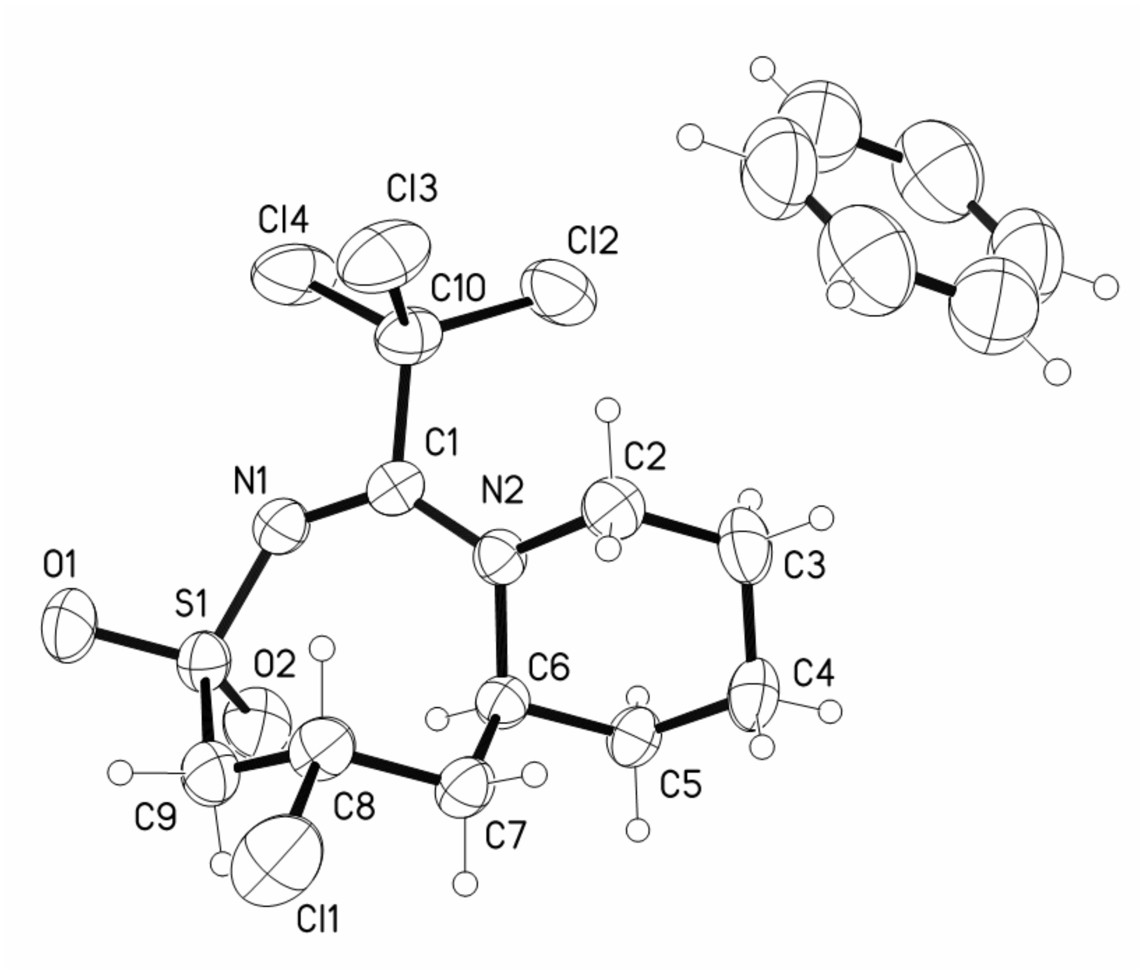


Fig. 7. Molecular structure of compound **14** including thermal displacement ellipses with 50% probability.

Crystal data for **17a**: $C_{10}H_9Cl_3N_2O_2S$, M= 327.62, monoclinic, space group *P2₁/c*, *a* = 13.8306(7), *b* = 7.7667(4), *c* = 12.7797(7)Å, $\beta$ = 110.031(4)°, V = 1289.73(12)Å$^3$, Z = 4, $d_c$ = 1. 687, μ 0.866 mm$^{-1}$, F(000) 664. All crystallographic measurements were performed at ambient temperature on a Bruker Smart Apex II diffractometer operating in the ω scans mode. The intensity data were collected within the $\theta_{max} \leq 27.25°$ using Mo-$K_\alpha$ radiation (λ = 0.71078 Å). The intensities of 10216 reflections were collected (2869 unique reflections, $R_{merg}$ = 0.042). The structure were solved by direct methods (SHELXS 86) and refined by the full-matrix least-squares technique in the anisotropic approximation for non-hydrogen atoms using the Crystals program package [2]. All CH hydrogen atoms were placed at calculated positions and refined as 'riding' model. Convergence was obtained at R1 = 0.0466 and wR = 0.0613 for 1747 observed reflections with I ≥ 3σ(I), GOF = 1.029; R1 = 0.0735 and wR = 0.0882 for 2860 independent reflections, 163 parameters in refinement, the largest and minimal peaks in the final difference map 0.56 and –0.64 e/Å$^3$. Any request to the CCDC for these materials should quote the full literature citation and reference number 2004900. The molecular structure of compound **17a** was shown on fig. 8.

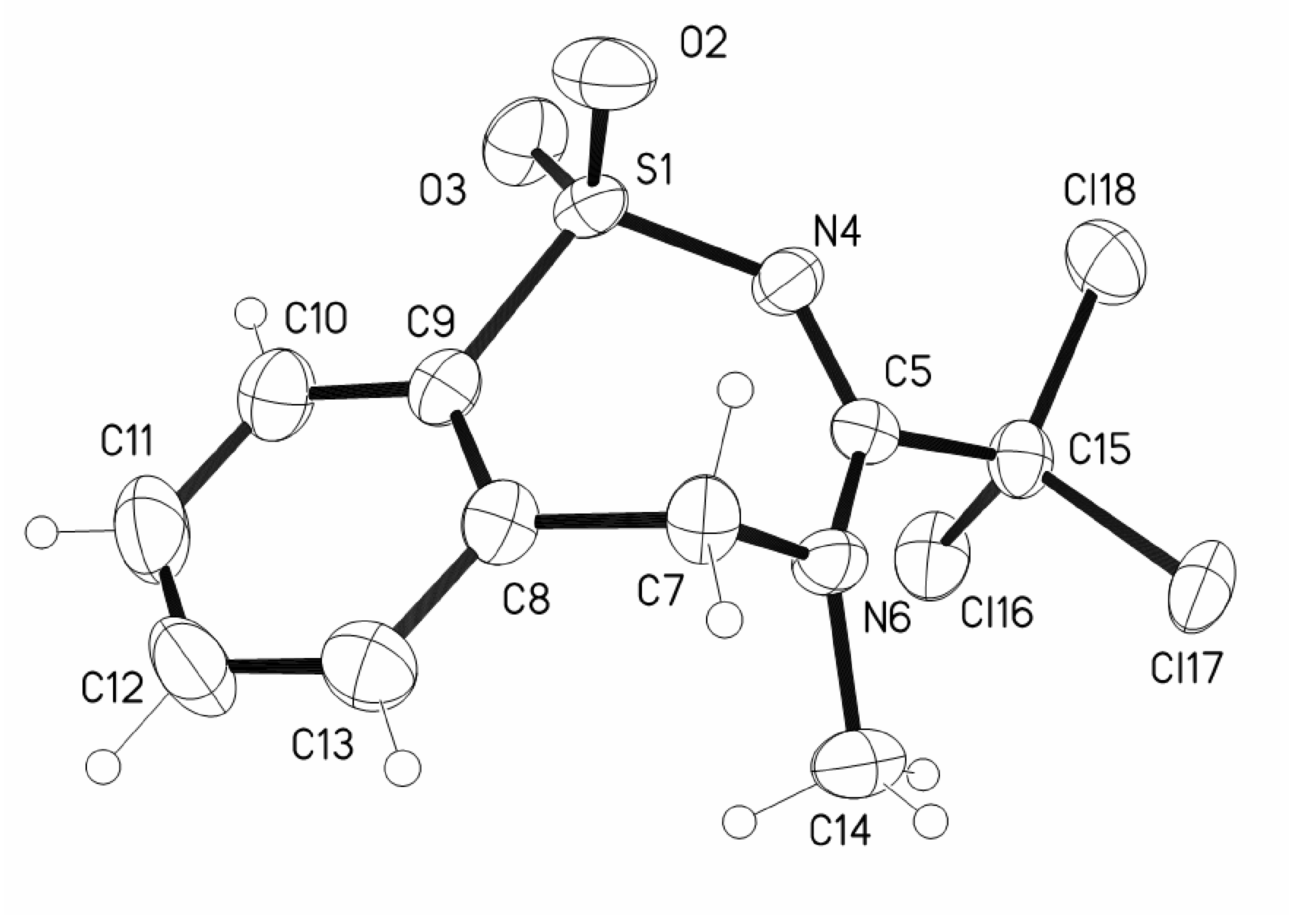


Fig. 8. Molecular structure of compound **17a** including thermal displacement ellipses with 50% probability.

Crystal data for **17b**: $C_{12}H_{13}Cl_3N_2O_2S$, M= 355.67, orthorhombic, space group $P2_12_12_1$, $a$ = 8.7561(2), $b$ = 13.0940(4), $c$ = 13.1625(4)Å, V = 1509.11(7)$Å^3$, Z = 4, $d_c$ = 1. 565, μ 0.746 $mm^{-1}$, F(000) 728. All crystallographic measurements were performed at ambient temperature on a Bruker Smart Apex II diffractometer operating in the ω scans mode. The intensity data were collected within the $\theta_{max} \leq 26.2°$ using Mo-$K_\alpha$ radiation (λ = 0.71078 Å). The intensities of 10918 reflections were collected (3018 unique reflections, $R_{merg}$ = 0.0301). The structure were solved by direct methods (SHELXS 86) and refined by the full-matrix least-squares technique in the anisotropic approximation for non-hydrogen atoms using the Crystals program package[2].

All CH hydrogen atoms were placed at calculated positions and refined as 'riding' model. Convergence was obtained at R1 = 0.0302 and wR = 0.0323 for 2570 observed reflections with I ≥ 3σ(I), GOF = 1.036; R1 = 0.0381 and wR = 0.0395 for 3007 independent reflections, 181 parameters in refinement, the largest and minimal peaks in the final difference map 0.26 and –0.23 e/$Å^3$. Any request to the CCDC for these materials should quote the full literature citation and reference number 2004901. The molecular structure of compound **17b** was shown on fig. 9.

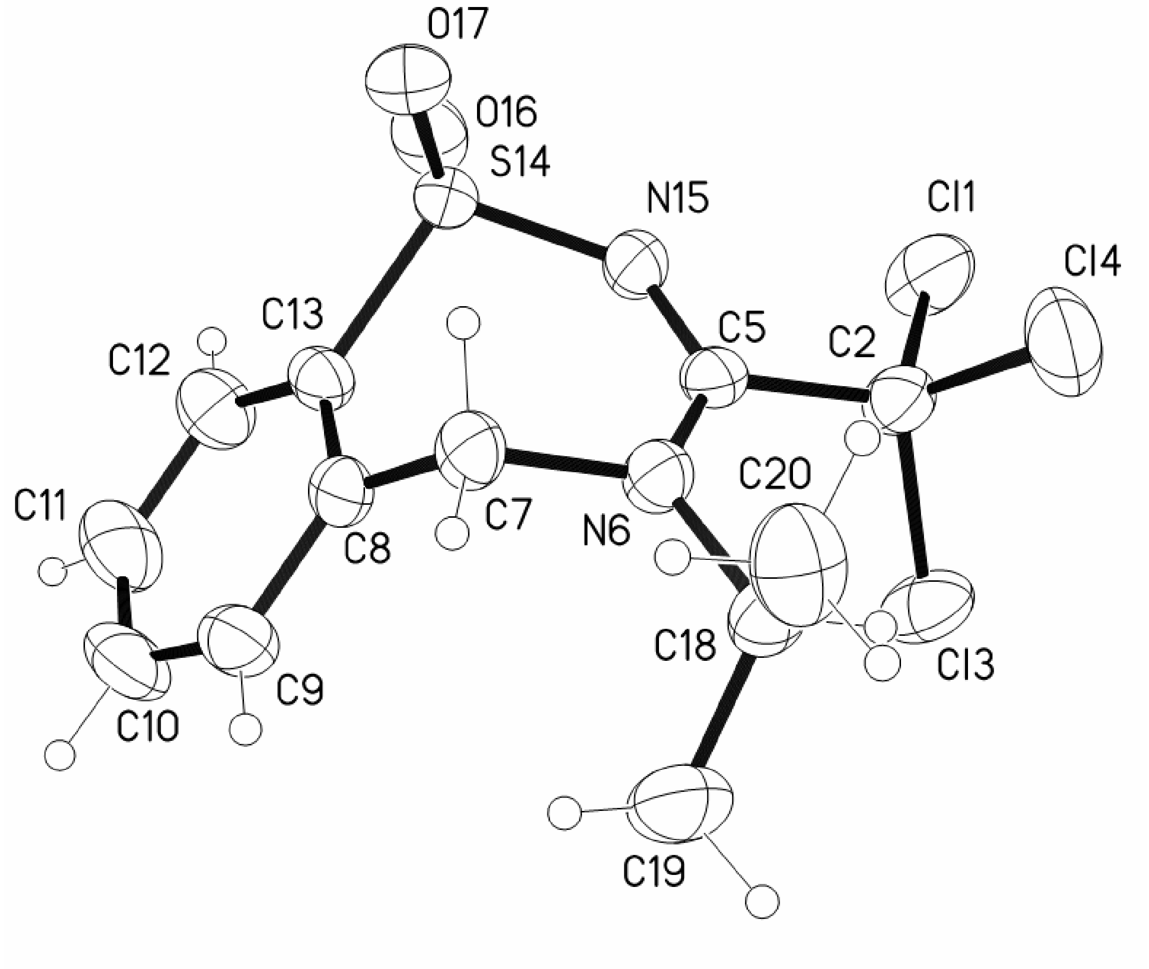


Fig. 9. Molecular structure of compound **17b** including thermal displacement ellipses with 50% probability.

Crystal data for **18**: $C_9H_7Cl_3N_2O_2S$, M= 313.59, triclinic, space group *P-1*, *a* = 6.5309(2), *b* = 9.5601(3), *c* = 10.0586(3)Å, $\alpha$ = 85.558(2), $\beta$ = 72.016(2), $\gamma$ = 84.417(2) °, V = 593.76(3)Å$^3$, Z = 2, $d_c$ = 1.754, μ 0.936 mm$^{-1}$, F(000) 316. All crystallographic measurements were performed at ambient temperature on a Bruker Smart Apex II diffractometer operating in the ω scans mode. The intensity data were collected within the $\theta_{max} \leq 26.57°$ using Mo-K$_\alpha$ radiation (λ = 0.71078 Å). The intensities of 6515 reflections were collected (2450 unique reflections, $R_{merg}$ = 0.021). The structure were solved by direct methods and refined by the full-matrix least-squares technique in the anisotropic approximation for non-hydrogen atoms using the Crystals program package [2]. All CH hydrogen atoms were placed at calculated positions and refined as 'riding' model. Convergence was obtained at R1 = 0.0296 and wR = 0.0337 for 2028 observed reflections with I ≥ 3σ(I), GOF = 0.9745; R1 = 0.0376 and wR = 0.0464 for 2441 independent reflections, 154 parameters in refinement, the largest and minimal peaks in the final difference map 0.29 and –0.27 e/Å$^3$. Any request to the CCDC for these materials should quote the full literature citation and reference number 2004902. The molecular structure of compound **18** was shown on fig. 10

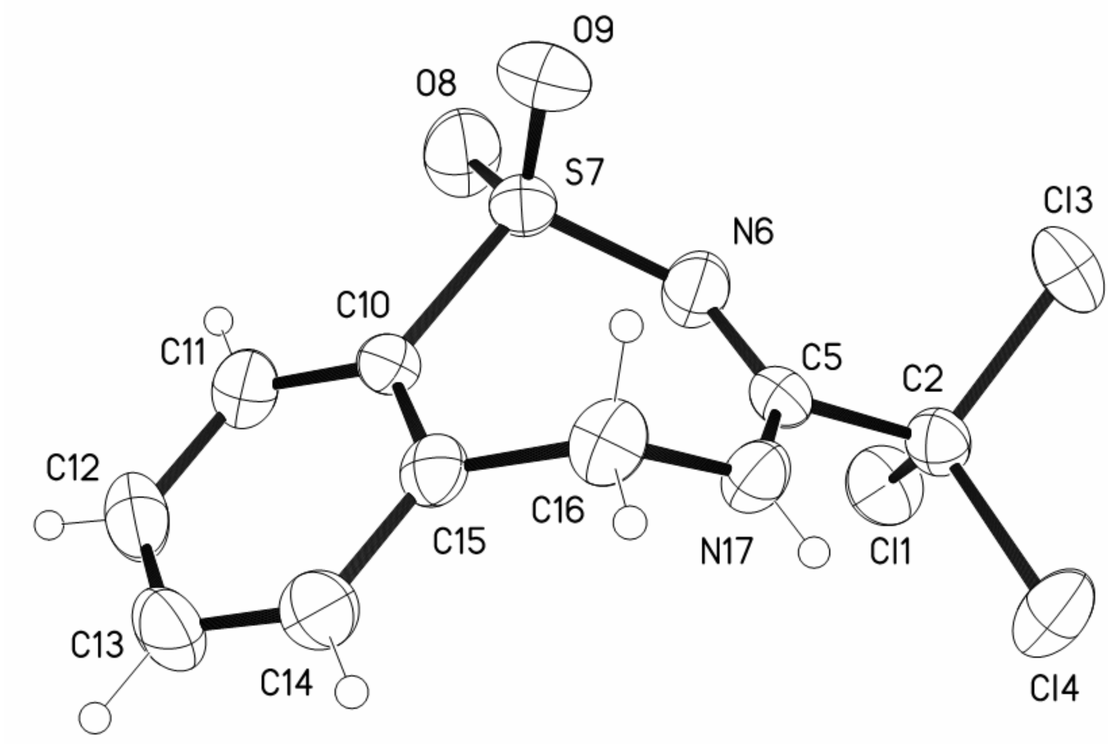


Fig. 10. Molecular structure of compound **18** including thermal displacement ellipses with 50% probability.

Crystal data for compound **19**: $C_{10}H_9Cl_3N_2O_2S$, M = 327.60, tetragonal, space group $P4_12_12$, $a$ = 9.4170(13), $c$ = 29.969(6)Å, V = 2657.6(9)Å$^3$, Z = 8, $d_c$ = 1.638 g·cm$^{-3}$, $\mu$ = 0.840 mm$^{-1}$, F(000) = 1328, crystal size ca. 0.1 × 0.25 × 0.42 mm. All crystallographic measurements were performed at ambient temperature on a Bruker Smart Apex II diffractometer operating in the ω scans mode. The intensity data were collected within the range of 2.27 ≤ θ ≤ 28.31° using Mo-$K_\alpha$ radiation (λ = 0.71078 Å). The intensities of 14747 reflections were collected (3291 unique reflections, $R_{merg}$ = 0.0360). The structure were solved by direct methods and refined by the full-matrix least-squares technique in the anisotropic approximation for most non-hydrogen atoms using the Bruker SHELXTL program package [1]. The chlorine atoms of $CCl_3$ group are disordered over tree position A B and C with multiplicity 0.859, 0.085 and 0.055 respectively, Cl atoms of position B and C with low occupancies where refined isotropically with geometric restraints for C-Cl distances. All CH hydrogen atoms were placed at calculated positions and refined as 'riding' model. Convergence was obtained at R1 = 0.0453 and wR2 = 0.0942 for 2618 observed reflections with I ≥ 2σ(I), R1 = 0. 0638 and wR2 = 0.1026, GOF = 1.039 for 3291 independent reflections, 194parameters, 7 restraints, the Flack parameter is -0.05(4), the largest and minimal peaks in the final difference map 0.40 and –0.40 e/Å$^3$. Full crystallographic details have been deposited at Cambridge Crystallographic Data Centre (CCDC). Any request to the CCDC for these materials should quote the full literature citation and reference number CCDC 2004903. The molecular structure of compound **19** was shown on fig. 11

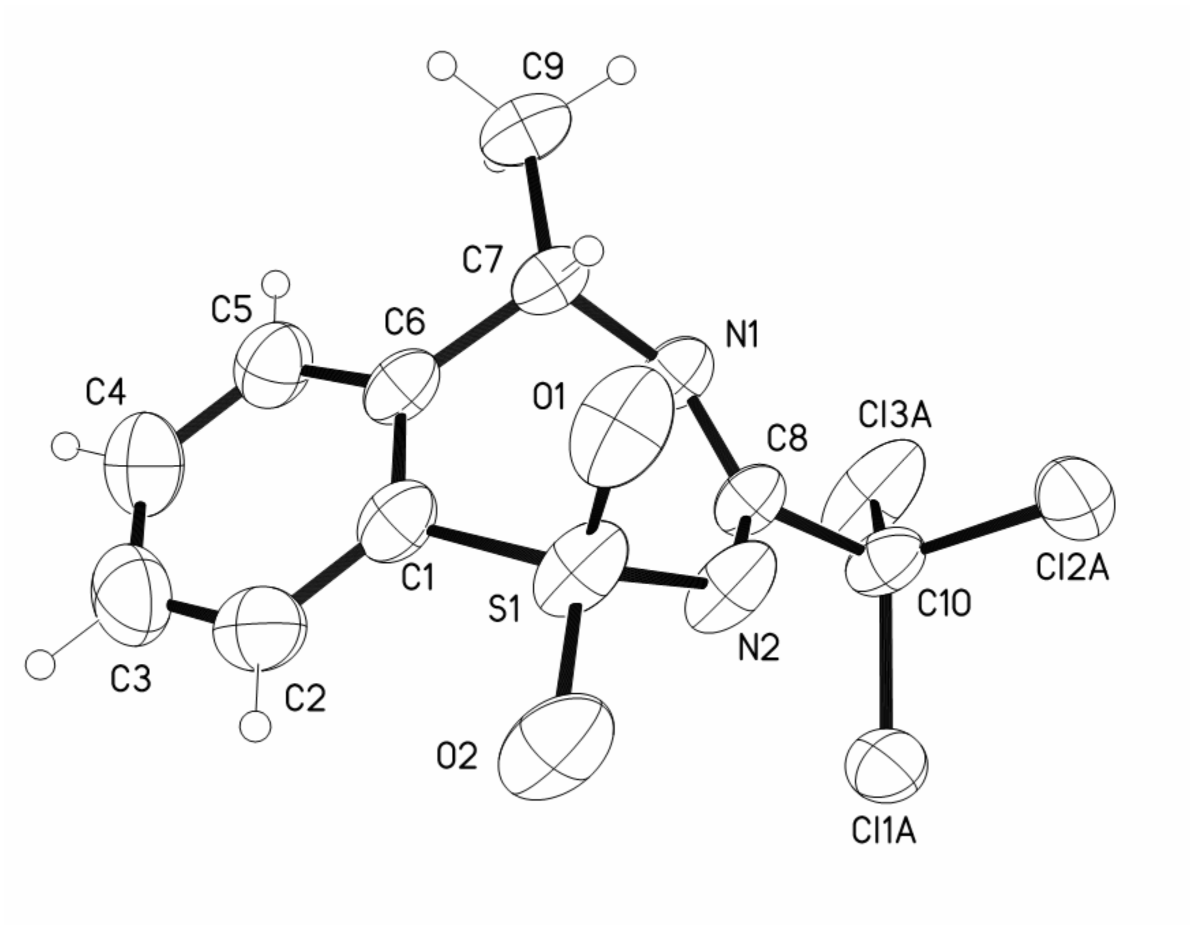


Fig. 11. Molecular structure of compound **19** including thermal displacement ellipses with 50% probability.

Crystal data for compound **21**: $C_6H_6Cl_4N_2O_2S$, M = 311.99, triclinic, space group *P-1*, *a* = 6.9878(11), *b* = 8.2771(11), *c* = 10.510(3)Å, *α* = 101.682(6), *β* = 96.598(7) *γ* = 107.503(5) °, V = 557.58(18) Å$^3$, Z = 2, $d_c$ = 1.858 g·cm$^{-3}$, μ = 1.227 mm$^{-1}$, F(000) = 312, crystal size ca. 0.10 × 0.28 × 0.48 mm. All crystallographic measurements were performed at 173K on a Bruker Smart Apex II diffractometer operating in the ω scans mode. The intensity data were collected within the range of 2.0 ≤ θ ≤ 28.4° using Mo-$K_\alpha$ radiation (λ = 0.71078 Å). The intensities of 7909 reflections were collected (2782 unique reflections, $R_{merg}$ = 0.0251). The structure were solved by direct methods and refined by the full-matrix least-squares technique in the anisotropic approximation for non-hydrogen atoms using the Bruker SHELXTL program package [1]. All CH hydrogen atoms were placed at calculated positions and refined as 'riding' model. Convergence was obtained at R1 = 0.0241 and wR2 = 0.0635 for 2493 observed reflections with I ≥ 2σ(I), R1 = 0. 0283 and wR2 = 0.0662, GOF = 1.042 for 2782 independent reflections, 137 parameters, the largest and minimal peaks in the final difference map 0.59 and –0.33 e/Å$^3$. Full crystallographic details have been deposited at Cambridge Crystallographic Data Centre (CCDC). Any request to the CCDC for these materials should quote the full literature citation and reference number CCDC 2004904. The molecular structure of compound **21** was shown on fig. 12.

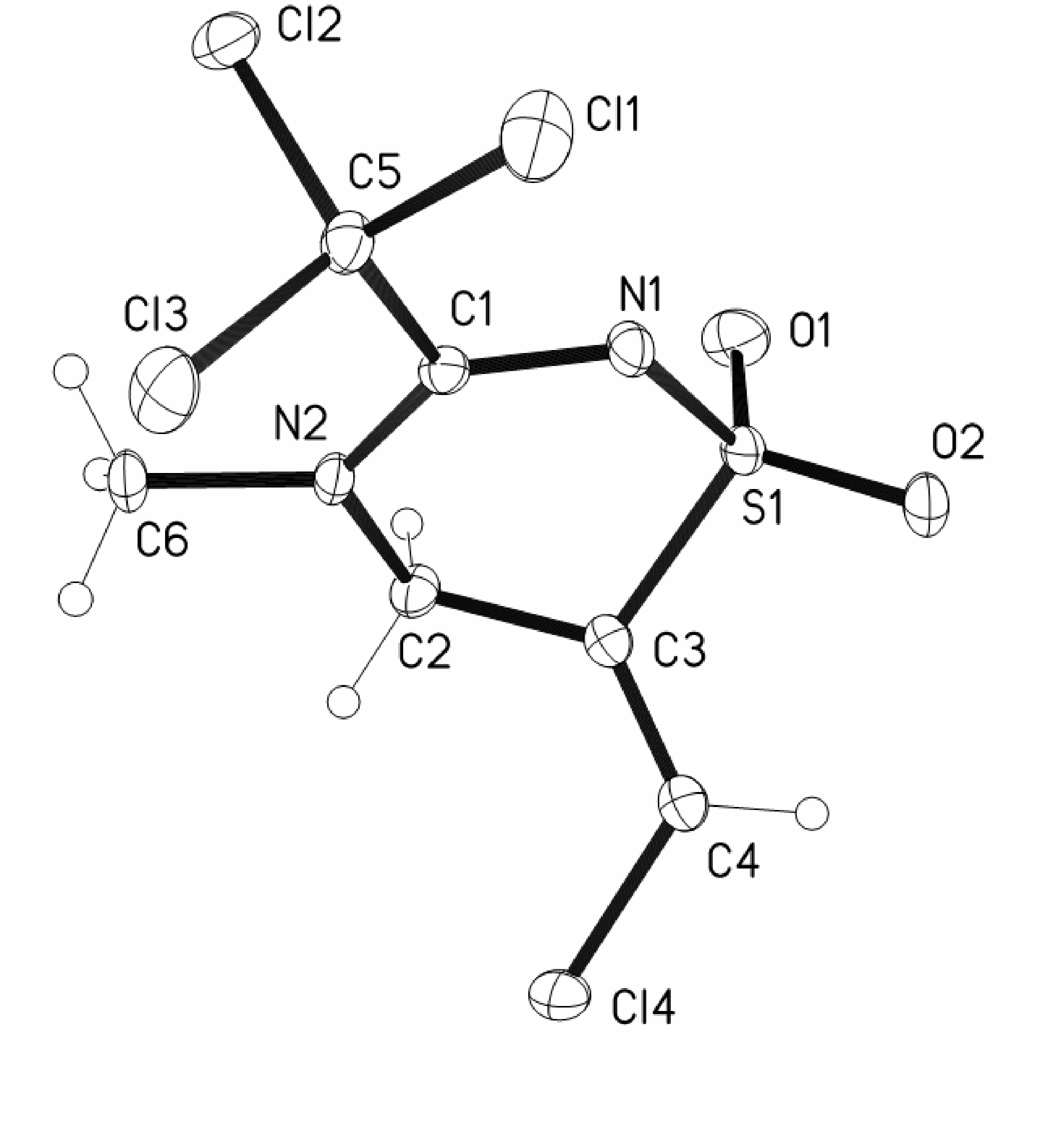


Fig. 12. Molecular structure of compound **21** including thermal displacement ellipses with 50% probability.

Crystal data for compound **23**: $C_{10}H_{12}C_{14}N_2O_2S$ x $C_6H_6$, M = 444.18, monoclinic, space group *P2₁/c*, *a* = 8.1755(2), *b* 9.8158(2), *c* = 24.3428(6)Å, *β* = 99.4711(18)°, V = 1926.86(8)Å$^3$, Z = 4, $d_c$ = 1.531 g·cm$^{-3}$, μ = 0.736 mm$^{-1}$, F(000) = 912, crystal size ca. 0.09 × 0.26 × 0.36 mm. All crystallographic measurements were performed at 173K on a Bruker Smart Apex II diffractometer operating in the ω scans mode. The intensity data were collected within the range of 2.2 ≤ θ ≤ 26.3° using Mo-$K_\alpha$ radiation (λ = 0.71078 Å). The intensities of 18756 reflections were collected (3923 unique reflections, $R_{merg}$ = 0.0472). The structure were solved by direct methods and refined by the full-matrix least-squares technique in the anisotropic approximation for non-hydrogen atoms using the Bruker SHELXTL program package [1]. All CH hydrogen atoms were placed at calculated positions and refined as 'riding' model. Convergence was obtained at R1 = 0.0412 and wR2 = 0.0943 for 3076 observed reflections with I ≥ 2σ(I), R1 = 0. 0589 and wR2 = 0.1029, GOF = 1.032 for 3926 independent reflections, 226 parameters, the largest and minimal peaks in the final difference map 0.40 and –0.42 e/Å$^3$. Full crystallographic details have been deposited at Cambridge Crystallographic Data Centre (CCDC). Any request to the CCDC for these materials should quote the full literature citation and reference number CCDC 2004905. The molecular structure of compound **23** was shown on fig. 13

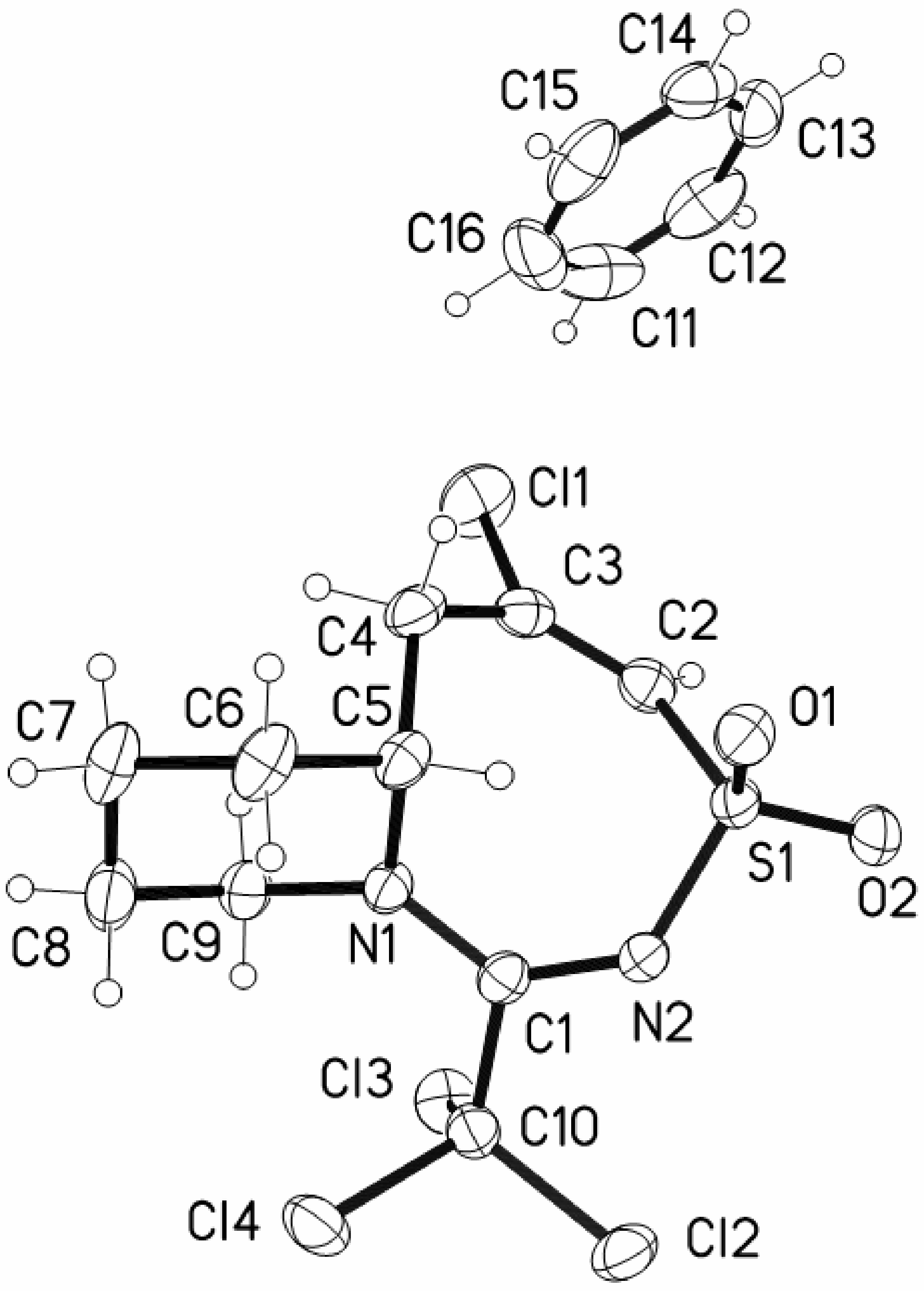


Fig. 13. Molecular structure of compound **23** including thermal displacement ellipses with 50% probability.

Crystal data for **27**: $C_9H_{10}N_2O_3S$, M= 226.26, monoclinic, space group *P2₁/c*, *a* = 9.3347(3), *b* = 12.9492(4), *c* = 8.2690(3)Å, *β* = 107.269(2)º, V = 954.47(6)Å$^3$, Z = 4, $d_c$ = 1. 574, μ 0.326 mm$^{-1}$, F(000) 472. All crystallographic measurements were performed at ambient temperature on a Bruker Smart Apex II diffractometer operating in the ω scans mode. The intensity data were collected within the $\theta_{max} \leq 26.36°$ using Mo-$K_\alpha$ radiation (λ = 0.71078 Å). The intensities of 11397 reflections were collected (1953 unique reflections, $R_{merg}$ = 0.029). The structure were solved by direct methods (SHELXS 86) and refined by the full-matrix least-squares technique in the anisotropic approximation for non-hydrogen atoms using the Crystals program package [2]. All CH hydrogen atoms were placed at calculated positions and refined as 'riding' model. Convergence was

obtained at R1 = 0.0315 and wR = 0.0327 for 1602 observed reflections with $I \geq 3\sigma(I)$, GOF = 1.110; R1 = 0.0397 and wR = 0.0379 for 1946 independent reflections, 163 parameters in refinement, the largest and minimal peaks in the final difference map 0.43 and –0.28 e/Å$^3$. Any request to the CCDC for these materials should quote the full literature citation and reference number 2004906. The molecular structure of compound **27** was shown on fig. 14.

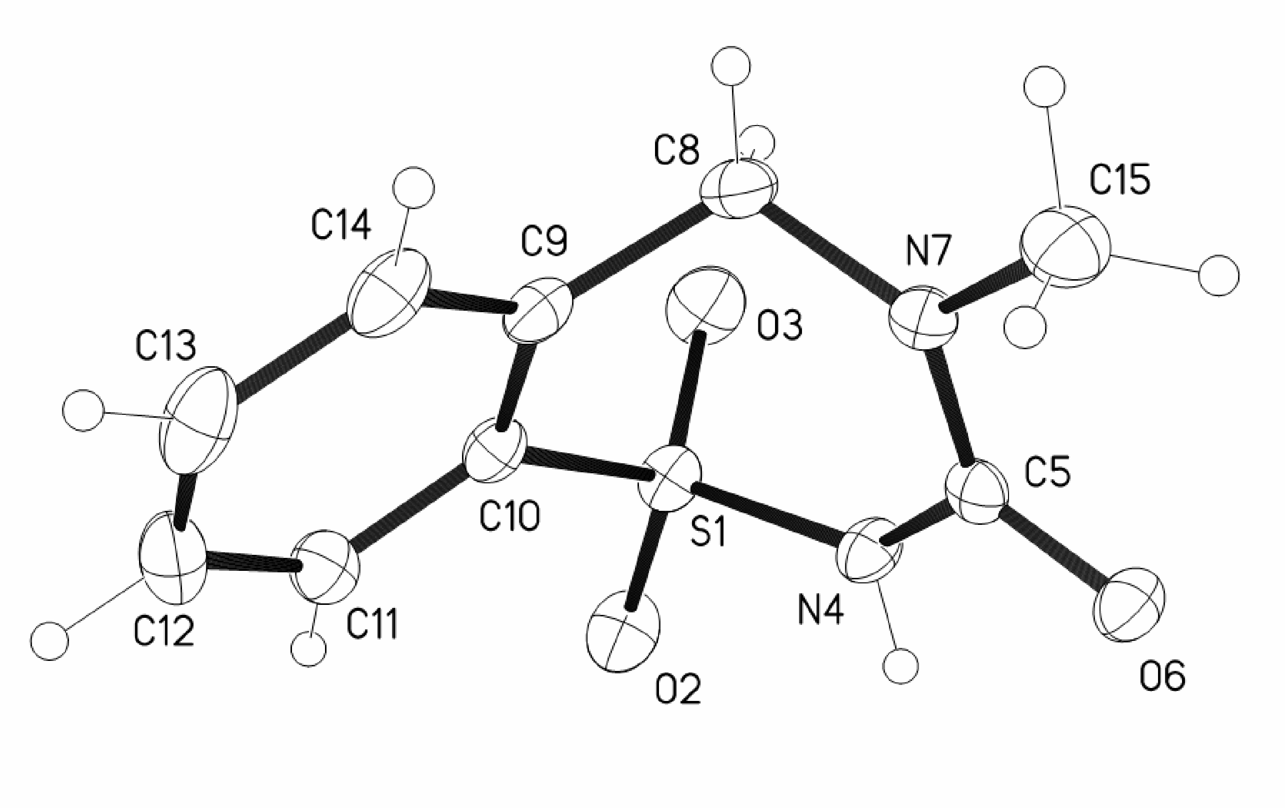


Fig. 14. Molecular structure of compound **27** including thermal displacement ellipses with 50% probability.

## References


[1] Sheldrick, G.M. A short history of SHELX. *Acta Cryst., Sect. A,* **2008**, *64*, 112-122.

[2] Betteridge P.W.; Carruthers J.R.; Cooper R.I.; Prout, K.; Watkin, D.J. CRYSTALS version 12: software for guided crystal structure analysis. *J. Appl. Cryst.* **2003**, *36*, 1487.

[3] Spek A.L. Single-crystal structure validation with the program PLATON. *J. Appl. Cryst*. **2003**, *36*, 7-13.

[4] van der Sluis, P; Spek, A.L. BYPASS: an effective method for the refinement of crystal structures containing disordered solvent regions. *Acta Cryst*., *Sect. A* **1990**, *46*, 194-201.

[5] Spek, A.L. *PLATON, a multipurpose crystallographic tool*; Utrecht University, Utrecht, The Netherlands, 1998.
